# Controlled Synthesis of Oligomers Containing Main‐Chain B(sp^2^)‐B(sp^2^) Bonds

**DOI:** 10.1002/chem.202103366

**Published:** 2021-10-11

**Authors:** Fabian Schorr, Nils Schopper, Nicolas Riensch, Felipe Fantuzzi, Marco Neder, Rian D. Dewhurst, Torsten Thiess, Tobias Brückner, Kai Hammond, Holger Helten, Maik Finze, Holger Braunschweig

**Affiliations:** ^1^ Institute for Inorganic Chemistry Julius-Maximilians-Universität Würzburg Am Hubland 97074 Würzburg Germany; ^2^ Institute for Sustainable Chemistry & Catalysis with Boron Julius-Maximilians-Universität Würzburg Am Hubland 97074 Würzburg Germany; ^3^ Institute for Physical and Theoretical Chemistry Julius-Maximilians-Universität Würzburg Emil-Fischer-Straße 42 97074 Würzburg Germany

**Keywords:** boron, catenation, diborane, hydroboration, oligomerization

## Abstract

A number of novel alkynyl‐functionalized diarylbis(dimethylamino)diboranes(4) are prepared by salt metathesis, and the appended alkynyl groups are subjected to hydroboration. Their reactions with monohydroboranes lead to discrete boryl‐appended diborane(4) species, while dihydroboranes induce their catenation to oligomeric species, the first known examples of well‐characterized macromolecular species with B−B bonds. The oligomeric species were found to comprise up to ten repeat units and are soluble in common organic solvents. Some of the oligomeric species have good air stability and all were characterized by NMR and vibrational spectroscopy and size‐exclusion chromatography techniques.

## Graphical abstract

**Novel alkynyl‐functionalized diarylbis(dimethylamino)diboranes(4)** were prepared and reacted with mono‐ and dihydroboranes, leading to hydroboration of the alkyne groups. The products are discrete boryl‐appended diborane(4) species and oligomeric species, and are the first known examples of well‐characterized macromolecular species with boron‐boron bonds. The oligomeric products were found to comprise up to ten repeat units, and are soluble in common organic solvents.

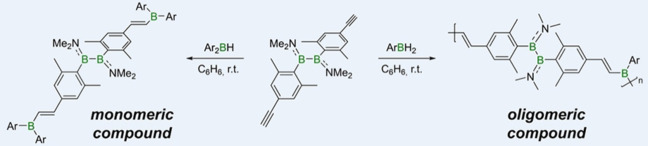

## Introduction

Functional polymers are a target of intense research interest due to their potential implementation in advanced electronics and many other fields.^[1]^ One potentially very interesting way to imbue functionality into a polymer is to incorporate tricoordinate, *sp* ^2^‐hybridized boron atoms, the empty *p* orbitals of which can accept electrons and/or donor units and thus act as sites for electronic, structural and even photophysical perturbation. However, due to the inherent synthetic difficulties and instability of the products – either real or imagined – successful syntheses of boron‐containing polymers remain rare.

**Figure 1:**
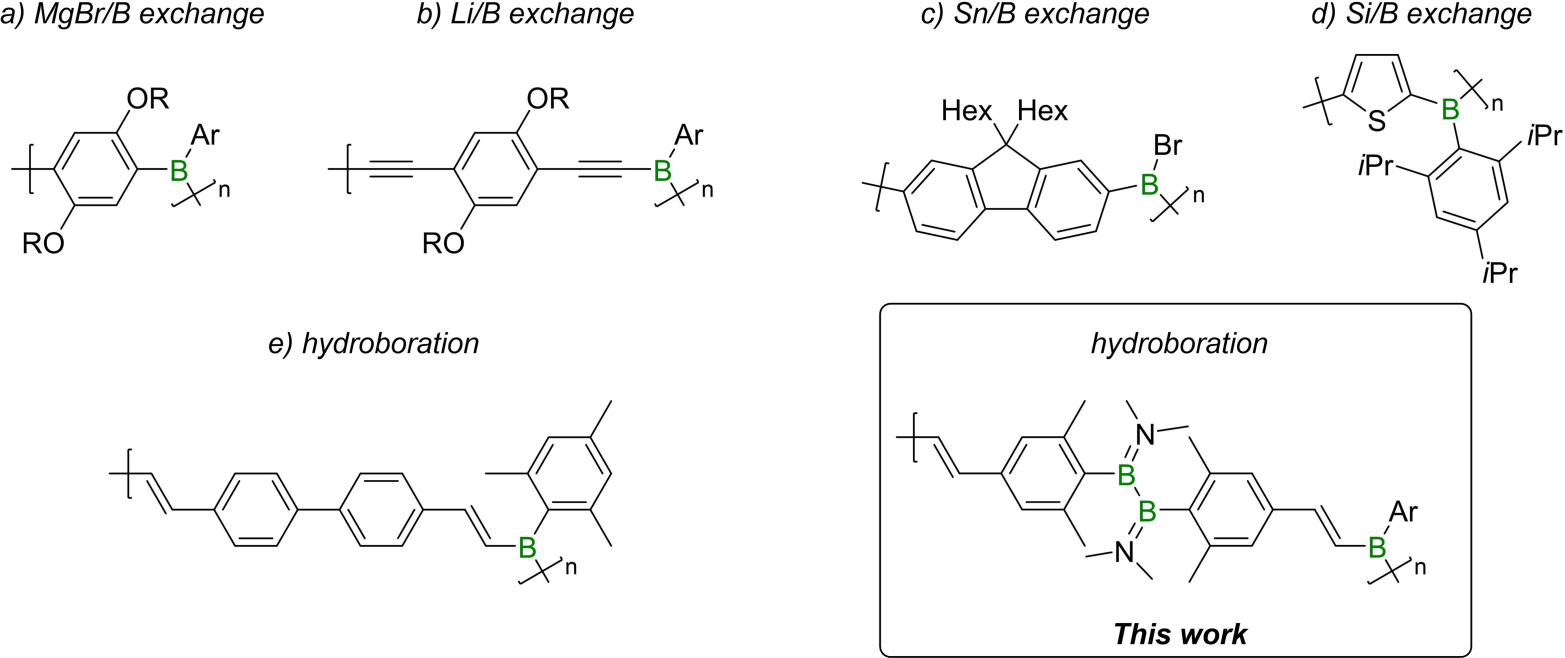
Examples of catenated organoborane species prepared by different element/boron exchange reactions or hydroboration protocols (R=C_12_H_25_ (*n*‐dodecyl); Ar=Mes (2,4,6‐trimethylphenyl)).

Chujo and coworkers performed pioneering work in the field of organoborane macromolecules in the 1990s,[^2^, ^3^, ^4^] leading to the incorporation of trivalent boron centers into organic chains by several different methods (Figure 1a,b). Initially, metallated aromatics, including Grignard and organolithium reagents, were reacted with alkoxyboranes to form catenated species via polycondensation reactions.^[2]^ While this method provided a number of novel materials, the high reactivity of the organometallic reagents, which must be generated in situ, was a distinct disadvantage. A more controllable system was later introduced by Jäkle et al. based on organotin compounds in the place of s‐block organyl metallates,^[5]^ leading to catenation under mild conditions with diverse substitution patterns (Figure 1c). In 2017, Helten and coworkers reported the use of less toxic organosilanes in a catalytic catenation process based on silicon/boron exchange (Figure 1d).^[6]^

In earlier work, the group of Chujo also reported a successful polymerization of organoboranes by hydroboration.^[3]^ Several poly(organoboranes) with good air and moisture stability were thereby prepared by combining sterically demanding aryldihydroboranes with diynes (Figure 1e).^[4]^

Although oligo‐ and polymeric boron‐containing species are now numerous and this field is growing rapidly, including even systems with carborane clusters in the main chain,^[7]^ macromolecular species containing electron‐precise boron‐boron bonds are currently unknown. Such a compound, effectively a poly(organyldiborane(4)), would likely open fascinating research avenues based on the inherent properties of diboranes(4),^[8]^ such as Lewis‐base addition to the vacant p(B) orbitals, redox chemistry to populate the empty π(B−B) orbitals, and exploiting the lability of the B−B bond. In this work we sought to redress this absence of poly(organyldiborane(4))‐type materials, and, inspired by Chujo's hydroboration polymerization work,^[4]^ we prepared a number of doubly alkynyl‐appended diboranes(4) based on the relatively stable 1,2‐diaryldiaminodiborane(4) scaffold as precursors for hydroboration‐based catenation. Herein we present the reactions of these alkynyl‐appended diboranes(4) with monohydroboranes, leading to discrete model bis(boryl)diboranes(4), and with dihydroboranes, providing the first examples of oligomeric species containing electron‐precise B−B bonds. The oligomeric species were found to comprise up to ten repeat units and were characterized by NMR, vibrational spectroscopy and size‐exclusion chromatography techniques.

## Results and Discussion

### Synthesis of alkynyl‐appended diboranes(4)

**Scheme 1:**
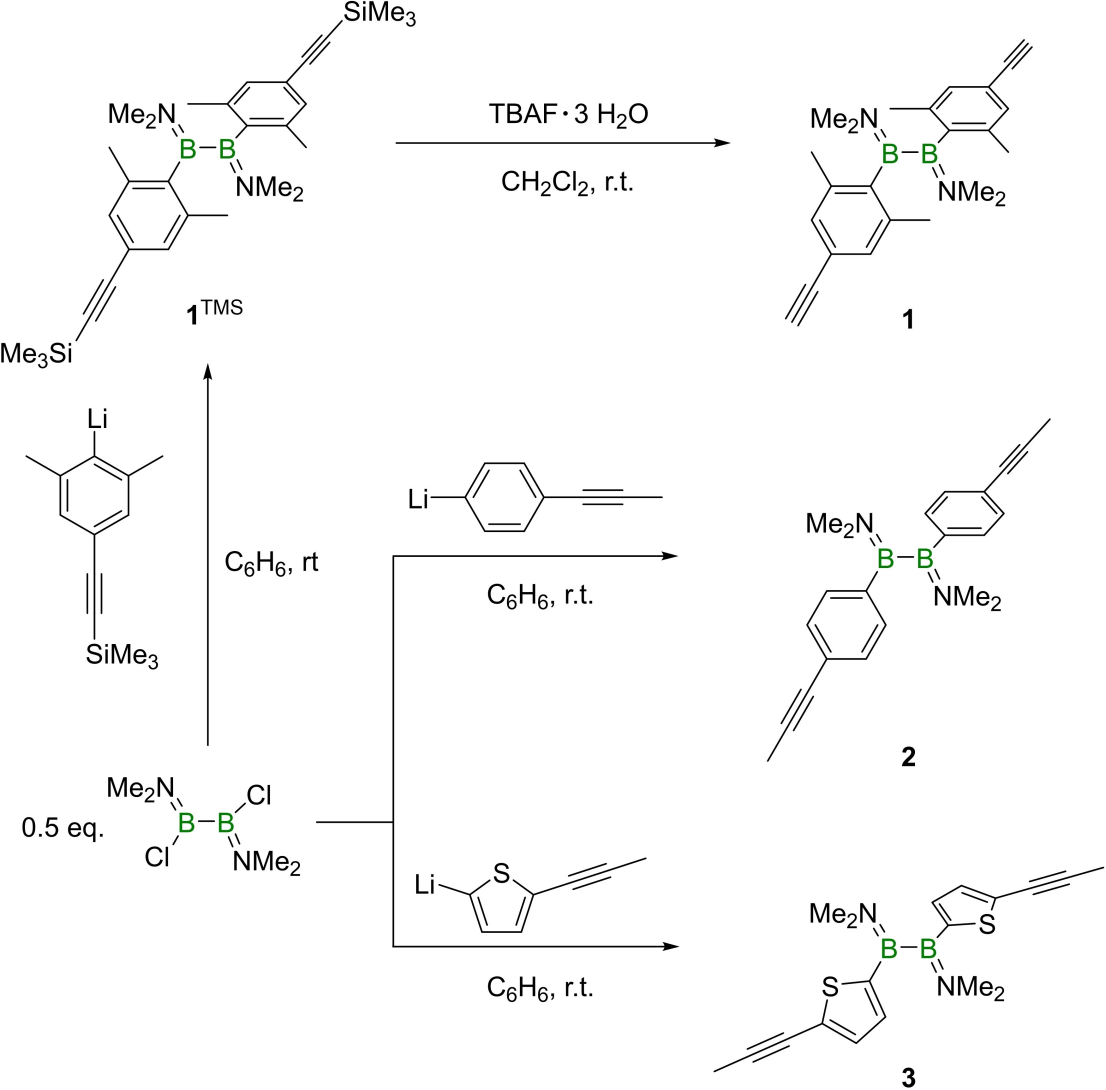
Synthetic approach to the alkynyl‐functionalized diboranes(4) **1**–**3**.

A typical salt‐elimination protocol based on combination of 1,2‐dichloro‐1,2‐bis(dimethylamino)diborane(4) with aryllithium reagents was used to prepare the alkynyl‐appended diboranes(4) **1** ^TMS^, **2**, and **3**.^[9]^ Due to the air and moisture stability of **1** ^TMS^, a subsequent fluorodesilylation step was employed to prepare terminal alkynyl species **1** (Scheme 1). The ^11^B NMR spectra of **1** and **2** exhibit signals at 49.0 ppm and 48.5 ppm, respectively, consistent with NMR data of previously reported 1,2‐diarylbis(dimethylamino)diboranes(4).^[9]^ The ^11^B NMR resonance of **3** (43.1 ppm) is significantly upfield of those of **1** and **2**, however, this was corroborated by the independent synthesis of 1,2‐dithieno‐1,2‐bis(dimethylamino)diborane(4) **4**, the ^11^B NMR resonance of which appeared at 44.1 ppm.

**Figure 2:**
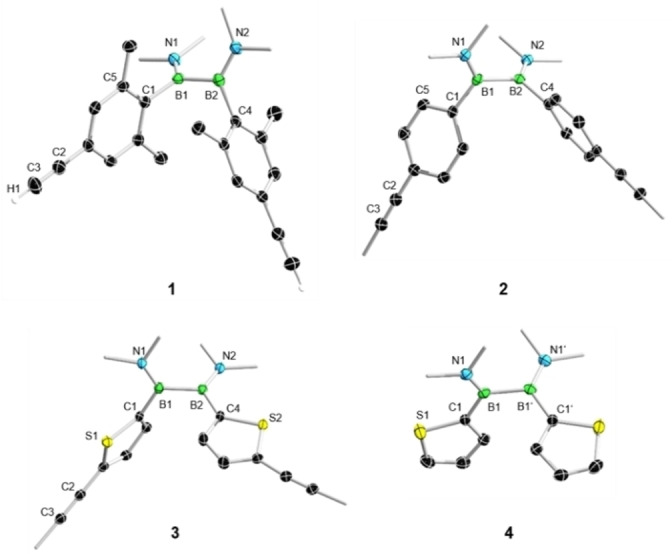
Crystallographically derived molecular structures of **1**–**4**. Thermal ellipsoids are drawn at the 50 % probability level. Ellipsoids on terminal methyl groups and selected hydrogen atoms have been omitted for clarity. Selected bond lengths (Å) and torsion angles (°) for **1**: B1‐B2 1.716(3), B1‐C1 1.593(2), C2‐C3 1.186(3); C5‐C1‐B1‐N1 100.8(2). **2**: B1‐B2 1.713(3), B1‐C1 1.591(3), C2‐C3 1.194(3); C5‐C1‐B1‐N1 68.9(3). **3**: B1‐B2 1.723(2), B1‐C1 1.584(2), C2‐C3 1.193(2); S1‐C1‐B1‐N1 48.5(2), S2‐C4‐B2‐N2 1.2(2). **4**: B1‐B2 1.721(3), B1‐C1 1.569(2); S1‐C1‐B1‐N1 2.9(2).

The solid‐state structures of compounds **1**–**4** were determined by single‐crystal X‐ray diffraction analysis, revealing a notable difference between the aryl‐ and heteroaryl‐substituted derivatives (Figure 2). While the aryl rings are twisted with respect to the B‐N plane in **1** and **2** (torsion angles: **1**: C5‐C1‐B1‐N1 100.8(2)°; **2**: C5‐C1‐B1‐N1 68.9(3)°), the thienyl groups of **4** are effectively coplanar with their attached B‐N planes (torsion angle: S1‐C1‐B1‐N1 2.9(2)°). Interestingly, the two thiophene rings of **3** reveal two markedly different torsion angles (S1‐C1‐B1‐N1 48.5(2)°; S2‐C4‐B2‐N2 1.2(2)°), presumably due to packing effects in the solid state.

### Hydroboration of alkynyl‐appended diboranes(4) with a monohydroborane

**Scheme 2:**
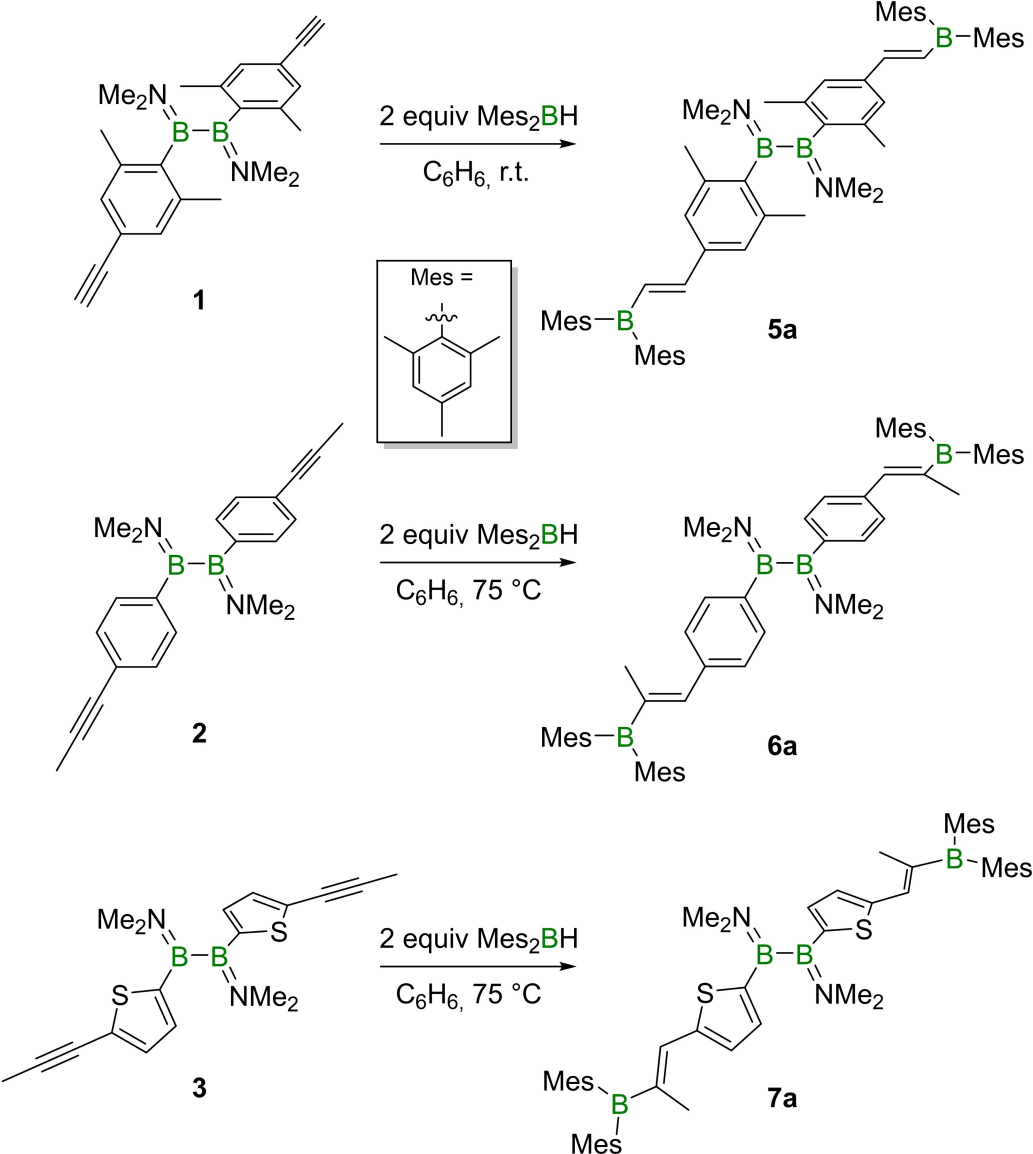
Synthesis of small‐molecule model compounds **5** **a**, **6** **a**, and **7** **a**.

With the alkynyl‐appended diboranes(4) **1**–**3** in hand, their propensity to undergo hydroboration was tested by their respective combination with two equivalents of dimesitylborane (Mes_2_BH) as shown in Scheme 2. While compound **1** reacts readily, conversion of **2** and **3** was only detected at elevated temperatures (75 °C), unsurprising given the generally lower reactivity of internal alkynes relative to terminal analogs. Each of the three diboryl‐appended diboranes(4) (**5** **a**, **6** **a**, and **7** **a**; Scheme 2) exhibit two broad ^11^B NMR resonances.

While the ^11^B NMR signals of the central B−B unit remain nearly unchanged (**5** **a** and **6** **a**: ca. 50 ppm, **7** **a**: 44.4 ppm), the resonances of the boryl groups (all ca. 75 ppm) were detected in the typical region for boranes bound to three sp^2^‐hybridized organyl groups.^[10]^

**Figure 3:**
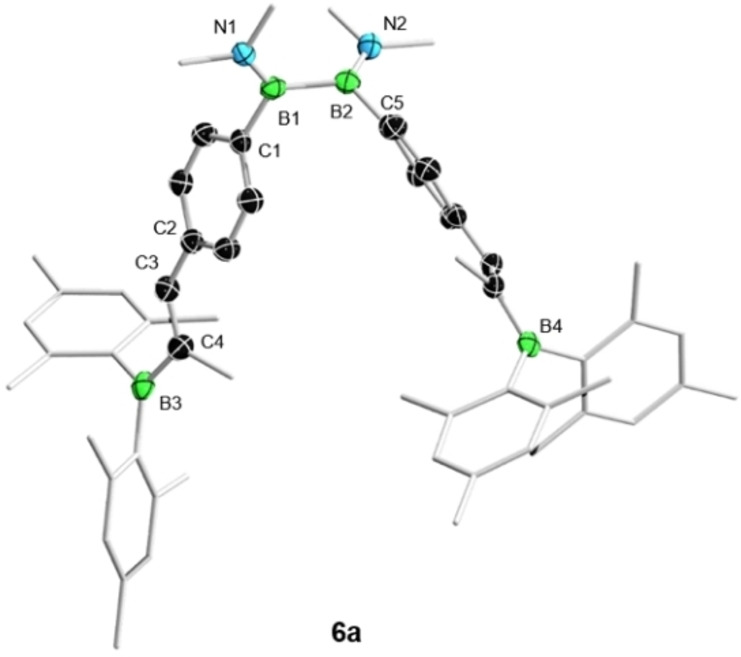
Crystallographically derived molecular structure of **6** **a**. Thermal ellipsoids are drawn at the 50 % probability level. Ellipsoids on methyl groups, selected rings, and hydrogen atoms have been omitted for clarity. Selected bond lengths (Å) and torsion angles (°) for **6** **a**: B1‐B2 1.716(4), B1‐C1 1.584(4), B3‐C4 1.560(4), C3‐C4 1.355(3); C2‐C3‐C4‐B3 170.5(2).

The Cahn, Ingold, and Prelog rules state that **6** **a** and **7** **a** should be denoted (*Z*) isomers, however, for ease of discussion these diboranes(4) will be referred to as *trans* isomers in the following alluding to their geometrical similarity with **5** **a**.^[11]^ Indeed, only this *trans* isomer is formed in **5** **a**, **6** **a** and **7** **a** (Scheme 2). The solid‐state structure of **6** **a** (Figure 3) confirmed its *trans* geometry, and that the central B−B unit (B−B 1.716(4) Å) remains unaffected by the hydroboration reaction (precursor **2**: B−B 1.713(3) Å).

**Figure 4:**
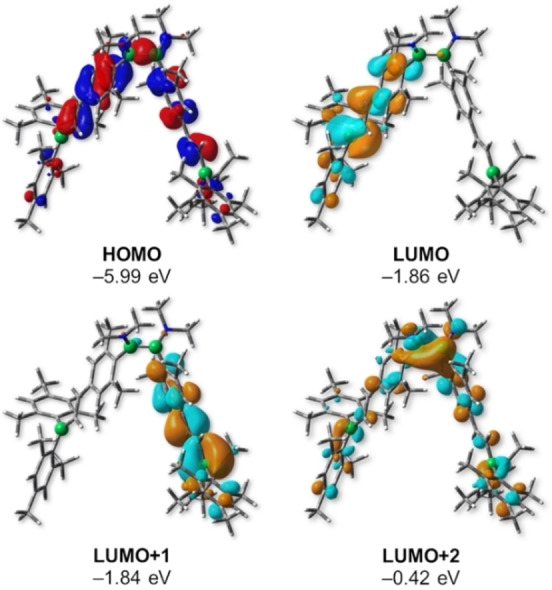
Frontier molecular orbitals (Kohn‐Sham) of model compound **5** **a** calculated at the PBE0‐D3(BJ)/def2‐SVP level of theory. Orbital energies are in eV. Isovalues: 0.03 a.u.

Calculations within the Kohn‐Sham density functional theory (DFT) at the PBE0‐D3(BJ)/def2‐SVP level were conducted for small‐molecule models **5** **a**, **6** **a**, and **7** **a**. The frontier molecular orbitals of **5** **a** are shown in Figure 4, while the corresponding orbitals of **6** **a** and **7** **a** can be found in the Supporting Information. The HOMO of **5** **a** indicates electron density at the B−B bond and the alkene and aryl spacer groups. The LUMO and LUMO+1 are nearly degenerate orbitals, respectively located on each half of the molecule. They are predominantly located at the boryl groups, with smaller contributions from the alkenyl and aryl spacer groups. These MOs lie ca. 1.5 eV lower in energy than LUMO+2, which in turn exhibits the π(B−B) contribution typical of diborane(4) compounds.

Consequently, the calculated HOMO‐LUMO gap of **5** **a** is 4.13 eV, around 2 eV smaller than those calculated for dialkynyldiboranes(4).^[12]^ The smaller HOMO‐LUMO gaps of the boryl‐appended diboranes(4) relative to the non‐borylated precursors are underlined by the significance differences in absoprtion maxima between precursor **1** (λ_max_ 263 nm) and borylated compounds **5** **a** (λ_max_ 348) and **6** **a** (λ_max_ 336). These differences likely reflect the lower energy of the LUMOs of the borylated species, which are dominated by the empty p orbitals of the boryl groups.

### Oligomerization of alkynyl‐appended diboranes(4) by hydroboration with a dihydroborane

**Scheme 3:**
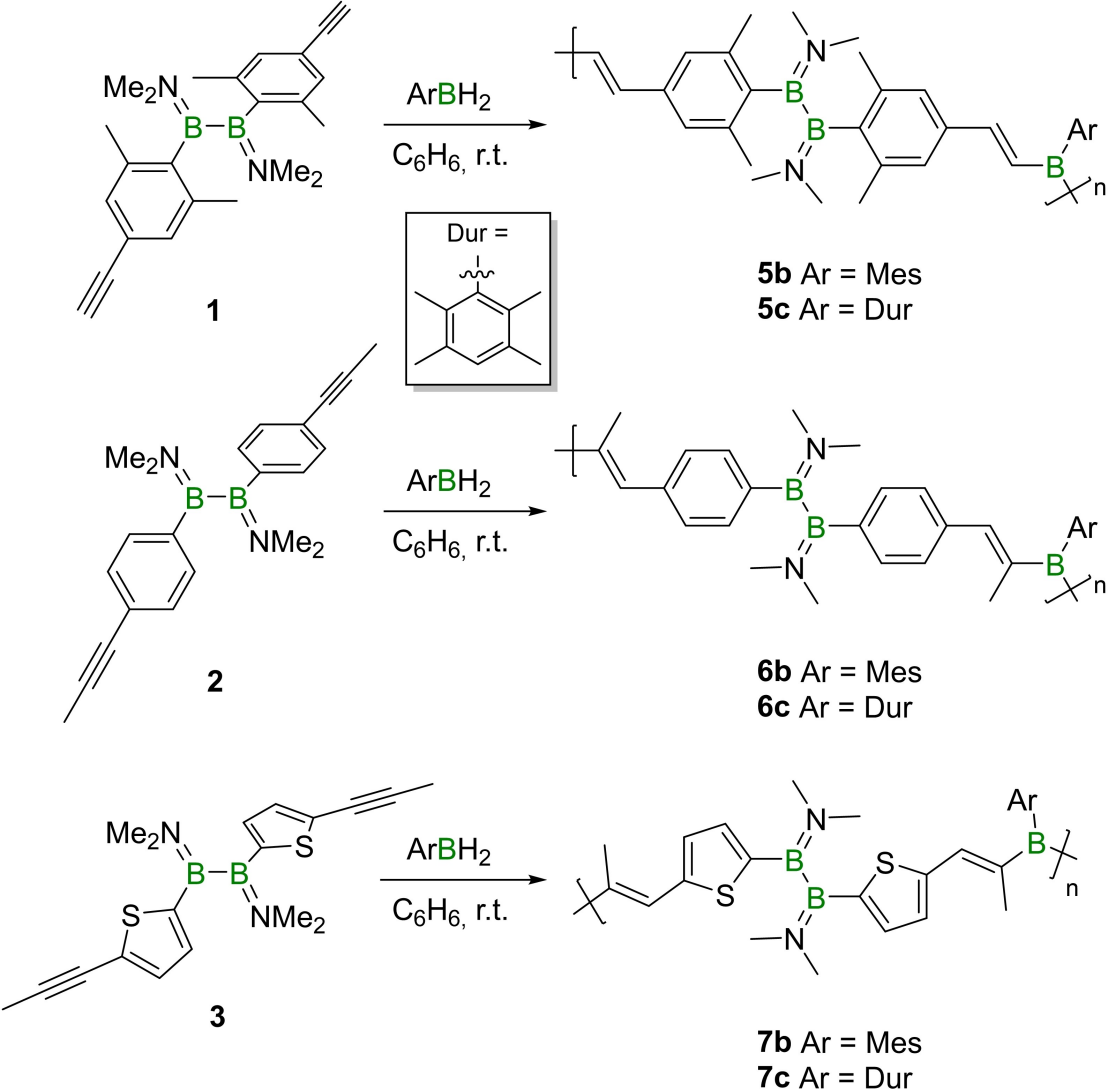
Synthesis of catenated species **5** **b,c**, **6** **b,c** and **7** **b,c**.

In order to investigate the possibility of hydroborylative oligomerization (Scheme 3), the precursors **1**–**3** were separately reacted with mesitylborane (MesBH_2_) and durylborane (DurBH_2_). In the reactions of **1** with MesBH_2_ and DurBH_2_, after a short conversion time (1 h), no appreciable ^11^B NMR resonances could be detected, however, the ^1^H NMR spectroscopic signals showed significant broadening.

The chemical shifts of these resonances were found to be in line with their counterparts in the ^1^H NMR spectrum of model compound **5** **a**, for instance, the signals corresponding to the protons of the dimethylamino group (**5** **a**: 3.09, 2.52 ppm, **5** **b**: 3.14, 2.57 ppm). Colorless solids were obtained from these reactions by removal of the benzene solvent under reduced pressure, washing with hexane, and drying. Further confirmation of the successful hydroboration reactions was provided by vibrational spectroscopy. Clear C=C stretching bands for **5** **b** (IR: 1587 cm^−1^; Raman: 1595 cm^−1^) and **5** **c** (IR: 1586 cm^−1^; Raman: 1595 cm^−1^) were found to have nearly identical frequencies to those of model compound **5** **a** (IR: 1585 cm^−1^; Raman: 1593 cm^−1^), as well as to a comparable poly(organylborane) reported by Chujo (IR: 1589 cm^−1^; Figure 1e).^4^ The absence of C−C triple bond stretches is also notable in these compounds.

In order to determine the chain length and molecular weight distribution of presumed oligomers **5** **b**,**c**, size‐exclusion chromatography (SEC) was performed with toluene as internal standard and using anhydrous solvent.

**Figure 5:**
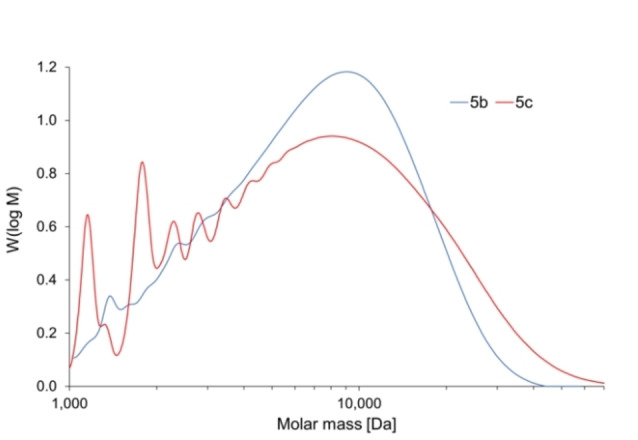
Molecular weight distribution of **5** **b** (blue) and **5** **c** (red); Detection via refractive index signal.

The molecular weight data for **5** **b,c** shown in Figure 5 and Table 1 reflect the successful construction of the target oligomers, indicating that these samples comprise ca. ten and nine repeat units, respectively. Remarkably, **5** **b,c** can be stored indefinitely in ambient air without noticeable decomposition as confirmed by ^1^H NMR spectroscopy.

**Table 1:** SEC data^[a]^ for compounds **5** **b,c**, **6** **b,c** and **7** **b,c**.

	M_n_ [Da]	M_w_ [Da]	PDI	DP_n_
**5** **b**	4824	8551	1.78	10
**5** **c**	4400	9644	2.19	9
**6** **b**	1041	2458	2.37	2
**6** **c**	1048	2080	1.98	2
**7** **b**	1473	2960	2.01	3
**7** **c**	603	884	1.47	1
**6** **c^[b]^**	1330	2281	1.71	3
**7** **b^[b]^**	1517	3239	2.14	3

[a] In THF, vs. polystyrene standards. [b] Data from TM‐catalyzed oligomerization.

Subsequently, compounds **2** and **3** were each treated in separate reactions with MesBH_2_ and DurBH_2_ similarly to the reactions described above. In each reaction a new doublet ^11^B NMR resonance was detected at 41.5 ppm with a ^1^ *J*_B‐H_ coupling constant of 109.3 Hz, suggesting the formation of a small‐molecule byproduct. This byproduct could be separated from the hydroborated oligomeric species by washing the crude products with hexane, providing **6** **b,c** and **7** **b,c**, respectively. Similarly to the case of **5** **a**‐**c**, while they showed no detectable ^11^B NMR spectroscopic signals, the chemical shifts of the ^1^H NMR spectroscopic signals of **6** **b,c** and **7** **b,c** were found to be very similar to the resonances of their small‐molecule counterparts **6** **a** and **7** **a**, respectively. The successful hydroboration processes were again underlined by data from vibrational spectroscopy.

All four compounds (**6** **b,c** and **7** **b,c**) exhibited signals in the typical region for C=C stretching vibrations. (e. g. **6** **b** IR: 1587 cm^−1^; Raman: 1595 cm^−1^).^[13]^

In contrast to **5** **a,b**, however, the SEC data for **6** **b,c** and **7** **b,c** indicate far less complete catenation (Table 1). The phenylene‐containing systems **6** **b**,**c** were found to comprise only two repetition units, respectively, while the thienyl‐containing species **7** **b,c** were found to comprise three and one repeat units. Interestingly, while all of the other oligomeric species were found to be colorless or very pale orange, **7** **b,c** presented as green solids.

**Scheme 4:**
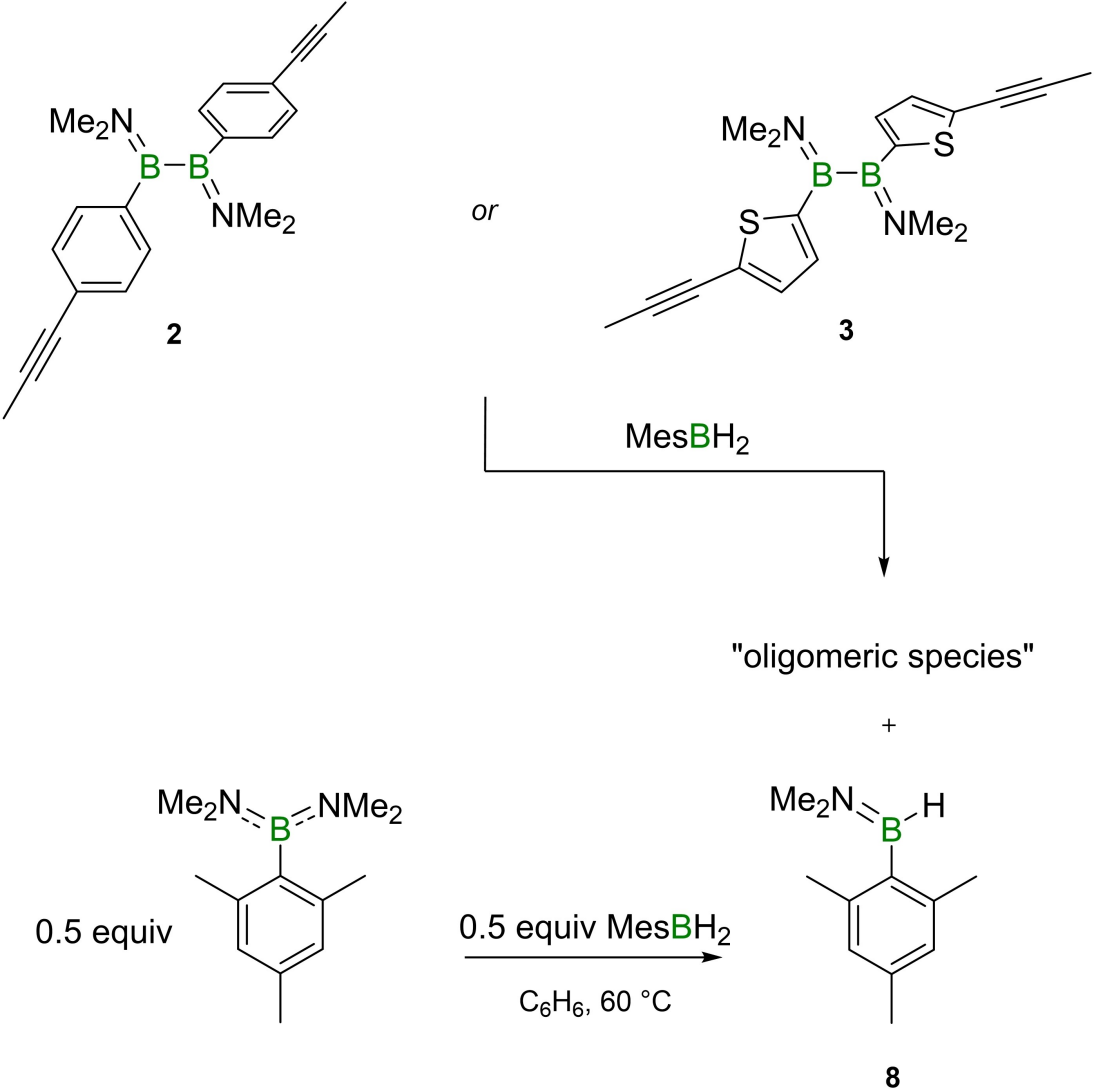
Side reaction in the oligomerization of **2** and **3**, and selective synthesis of byproduct **8**.

The results in Table 1, combined with the observation of a small‐molecule byproduct in the reactions of dihydroboranes with **2** and **3** (but not **1**), suggest that the extent of the polymerization process is strongly impacted by a side reaction. Fortuitously, we were able to isolate and characterize one of these side products, which was identified as dimethylaminomesitylborane HB(NMe_2_)(Mes) (**8**; Scheme 4). Its ^11^B, ^1^H and ^13^C NMR spectroscopic data are in accordance with comparable aminoarylboranes.^[14]^ In order to provide conclusive proof for the identity of this byproduct, **8** was independently synthesized by a comproportionation reaction of MesBH_2_ with MesB(NMe_2_)_2_. Similar σ‐bond metathesis reactions of arylboranes with aminoarylboranes are known from the literature,^[15]^ and it is thus highly likely that the byproduct found in the reaction with DurBH_2_ is the analogous dimethylaminodurylborane HB(Dur)(NMe_2_).

### Exploration of transition‐metal‐catalyzed hydroboration for polymerization

**Scheme 5:**
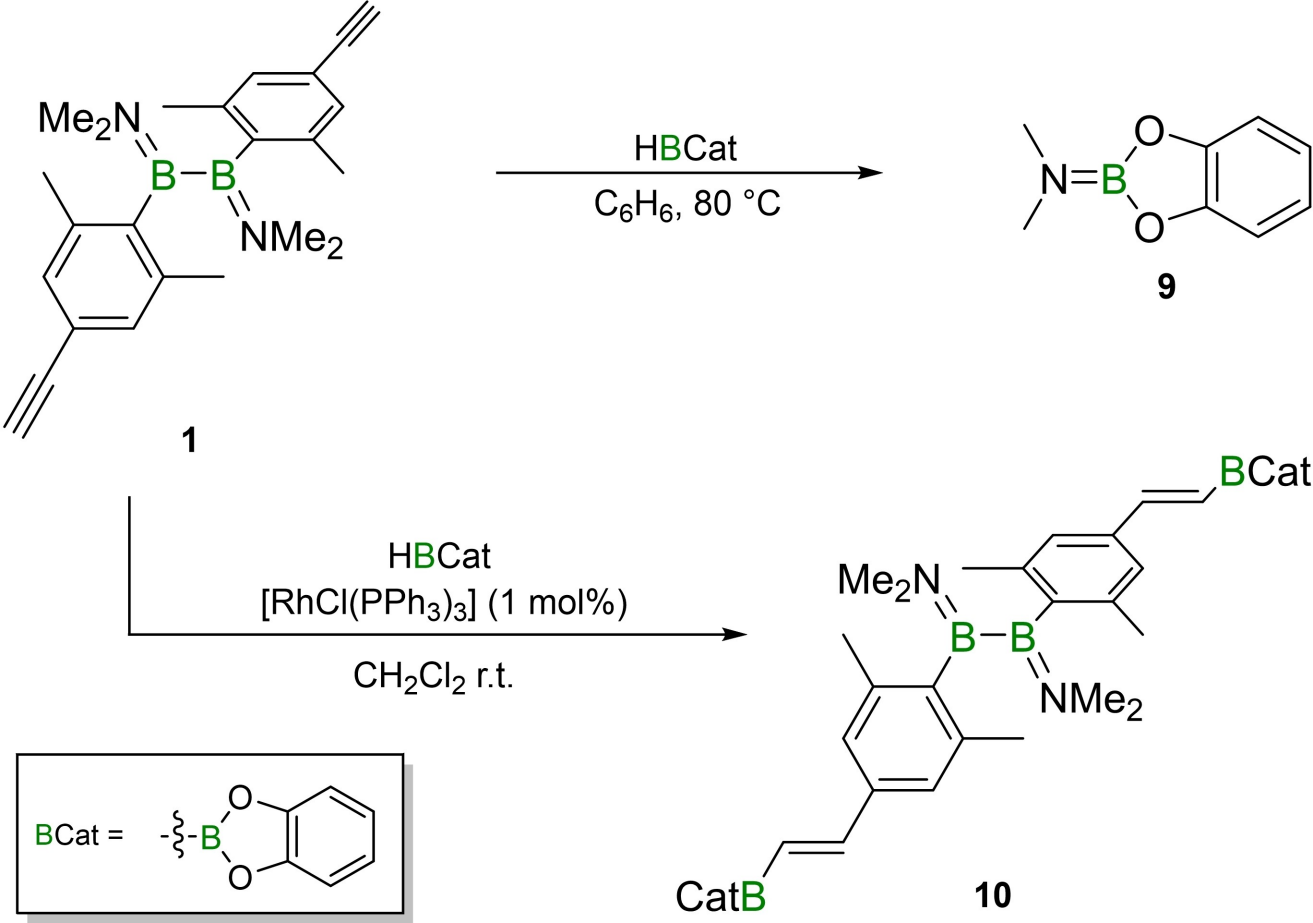
Reaction of **1** with catecholborane in the absence and presence of Wilkinson's catalyst.

Given the difficulties apparent in the polymerization reactions of **1**–**3**, we looked to other model systems for guidance. Diborane **1** was found to be inert to the presence of catecholborane (HBCat) at ambient temperature, while heating to 80 °C resulted only in the isolation of dimethylaminocatecholborane **9** (Scheme 4; see Supporting Information for spectroscopic and crystallographic data). Alternatively, the use of Wilkinson's catalyst ([RhCl(PPh_3_)_3_]; 1 mol%)^[16]^ in this reaction results in clean two‐fold hydroboration of **1** at ambient temperature, providing **10** in 84 % yield (Scheme 5). The ^11^B NMR spectrum of **10** exhibits a resonance resulting from the B_2_ unit at 50.0 ppm and another signal at 32.0 ppm for the BCat groups. The ^1^H NMR spectrum reveals two doublet resonances with a coupling constant of 18.5 Hz, indicating the selective synthesis of the *trans* isomer. Data from vibrational spectroscopy confirmed the formation of the borylated alkene moieties, with typical C=C stretching bands (**10** IR: 1619 cm^−1^, 1548 cm^−1^; Raman: 1621 cm^−1^, 1598 cm^−1^).^[13]^

Prompted by the effective promotion of the hydroboration by a transition metal catalyst, we repeated the reactions of **2** and **3** with both dihydroboranes in the presence of various loadings of Wilkinson's catalyst and at different temperatures (−78 °C, 0 °C, r.t.). However, no significant improvement was observed in the extent of polymerization in these reactions (see last two entries Table 1), as the side reaction still occurs.

## Conclusion

Herein we present the synthesis of a number of alkyne‐substituted 1,2‐di(hetero)arylbis(dimethylamino)diboranes(4) and demonstrate the hydroboration of their alkyne moieties to prepare small‐molecule model compounds containing four boron atoms. The three synthesized alkynyl‐appended diboranes(4) were then oligomerized by treatment with an aryldihydroborane, leading to the first organoborane oligomers containing electron precise B−B bonds, a number of which exhibited good stability towards air and moisture.

### X‐ray Crystallography

Deposition Numbers 2098158 (for **1**), 2098159 (for **2**), 2098160 (for **3**), 2098161 (for **4**), 2098162 (for **6a**), 2098163 (for **9**) contain the supplementary crystallographic data for this paper. These data are provided free of charge by the joint Cambridge Crystallographic Data Centre and Fachinformationszentrum Karlsruhe Access Structures service.”

## Conflict of interest

The authors declare no conflict of interest.

## Supporting information

As a service to our authors and readers, this journal provides supporting information supplied by the authors. Such materials are peer reviewed and may be re‐organized for online delivery, but are not copy‐edited or typeset. Technical support issues arising from supporting information (other than missing files) should be addressed to the authors.

Supporting Information
