# Supplementary material for: Controlled Synthesis of Oligomers Containing Main‐Chain B(sp2)‐B(sp2) Bonds
Source: Chemistry. 2021 Oct 11;27(64):16043–8. doi: 10.1002/chem.202103366 (PMC9292976; doi:10.1002/chem.202103366)
Supplement: Supplementary file 1 — Supporting Information [file CHEM-27-16043-s001.pdf]

Chemistry–A European Journal

Supporting Information

Controlled Synthesis of Oligomers Containing Main-Chain B(sp^2)-B(sp^2) Bonds

Fabian Schorr, Nils Schopper, Nicolas Riensch, Felipe Fantuzzi, Marco Neder, Rian D. Dewhurst, Torsten Thiess, Tobias Brückner, Kai Hammond, Holger Helten, Maik Finze, and Holger Braunschweig*

Contents

Methods and materials....2

Synthetic procedures 3

NMR spectra of isolated compounds 10

IR spectra....47

Raman spectra 62

Size exclusion chromatography (SEC)....75

UV-vis spectra....83

X-ray crystallographic data 87

Computational details....91

Cartesian coordinates....93

References 111

Methods and materials

All manipulations were performed either under an atmosphere of dry argon or *in vacuo* using standard Schlenk line or glovebox techniques. Deuterated solvents were dried over molecular sieves and degassed by three freeze-pump-thaw cycles prior to use. All other solvents were distilled and degassed from appropriate drying agents. Both deuterated and non-deuterated solvents were stored under argon over activated 4 Å molecular sieves. NMR spectra were acquired either on a Bruker Avance 500 or a Bruker Avance 400 NMR spectrometer. Chemical shifts (δ) are reported in ppm and internally referenced to the carbon nuclei ($^{13}C\{^1H\}$) or residual protons (^1H) of the solvent. Heteronuclei NMR spectra are referenced to external standards (^{11}B: $BF_3{\cdot}OEt_2$, ^{29}Si: $Si(CH_3)_4$). Solid-state IR spectra were recorded on a Bruker FT-IR spectrometer ALPHA II inside a glovebox. Raman spectra were recorded at room temperature with a MultiRAM FT-Raman spectrometer using the 1064 nm excitation line of an Nd/YAG laser on crystalline samples contained in melting point capillaries in the region of 3500–100 cm^{-1}. Microanalyses (C, H, N, S) were performed on an Elementar vario MICRO cube elemental analyser. High-resolution mass spectrometry (HRMS) data were obtained from a Thermo Scientific Exactive Plus spectrometer. SEC chromatograms were recorded on an Agilent 1260 Infinity II Series, equipped with one SDV precolumn of 8x50 mm and three SDV linear columns of 8x300 mm in THF with a flow rate of 1 mL min^{-1} and toluene as internal standard at 25 °C, calibrated against polystyrene standards. Detection was carried out via UV signal (l = 254 and 280 nm) and refractive index (RI), respectively. Evaluation of the chromatograms was performed by using WinGPC software. UV-vis spectra were measured on a METTLER TOLEDO UV-vis-Excellence UV5 spectrophotometer inside a glovebox.

Unless otherwise noted, solvents and reagents were purchased from Sigma-Aldrich, Fisher Scientific or Alfa Aesar. $B_2Cl_2(NMe_2)_2$,[1] ((4-bromo-3,5-dimethylphenyl)ethynyl)trimethylsilane,[2] 1-bromo-4-(propyn-1-yl)benzene,[3] mesityleneborane[4] and dimesityleneborane[5] were synthesized by following literature procedures. 2-bromo-5-(propyn-1-yl)thiophene[3] and bis(dimethylamino)mesityleneborane[6] were synthesized according to literature procedures.

Synthetic procedures

$B_2(C_6H_2$-2,6-Me_2-4-$(CCSiMe_3))_2(NMe_2)_2$, 1^{TMS}

A solution of *t*-butyllithium (1.9 M, 14.9 mL, 28.4 mmol, 4.2 equiv) in pentane was added dropwise at 0 °C to a solution of ((4-bromo-3,5-dimethylphenyl)ethynyl)trimethylsilane (4.00 g, 14.2 mmol, 2.1 equiv.) in 200 mL of hexane. The resulting solution was warmed up and stirred for 16 h at ambient temperature prior to removing of all volatiles. Benzene (200 mL) was then added, the resulting orange suspension was cooled to 5 °C and a solution of bis(dimethylamino)dichlorodiborane(4) (1.22 g, 6.76 mmol) dissolved in benzene (50 mL) were added dropwise. The resulting suspension was heated at 70 °C for 3 h with stirring prior to removal of all volatiles. The obtained residue was suspended in 30 mL of hexane and filtered through a 2 cm silica column. After evaporation of all volatiles **1^{TMS}** (2.88 g, 5.61 mmol, 83% yield) was obtained as a pale yellow solid. ^1H NMR (500 MHz, CD_2Cl_2, 243 K): δ = 6.91 (s, 2H, $C^{Ar}H$), 6.73 (s, 2H, $C^{Ar}H$), 3.10 (s, 6H, $N(CH_3)_2$), 2.54 (s, 6H, $N(CH_3)_2$), 2.15 (s, 6H, $C^{Ar}CH_3$), 1.30 (s, 6H, $C^{Ar}CH_3$), 0.19 (s, 18H, $Si(CH_3)_3$) ppm. $^{13}C\{^1H\}$ NMR (126 MHz, $CDCl_3$, 243 K): δ = 147.9 (BC^{Ar}), 138.0 ($C^{Ar}CH_3$), 137.3 ($C^{Ar}CH_3$), 129.4 ($C^{Ar}H$), 128.6 ($C^{Ar}H$), 119.4 ($C^{Ar}C_q$), 106.2 ($C^{Ar}C_q$), 92.1 (SiC_q), 44.5 ($N(CH_3)_2$), 40.3 ($N(CH_3)_2$), 22.3 ($C^{Ar}CH_3$), 20.1 ($C^{Ar}CH_3$), 0.1 ($Si(CH_3)_3$) ppm. ^{11}B NMR (160 MHz, CD_2Cl_2, 297 K): δ = 49.3 (s) ppm. ^{29}Si NMR (99 MHz, $CDCl_3$, 297 K): δ = –18.4 (s) ppm. Solid-state IR: ν(C≡C) = 2148 cm^{-1}. HRMS LIFDI for $[C_{30}H_{46}B_2N_2Si_2]^+$ = $[M + H]^+$: calcd. 512.3380; found 512.3384.

$B_2(C_6H_2$-2,6-Me_2-4-$(CCH))_2(NMe_2)_2$, 1

To a solution of **1^{TMS}** (3.03 g, 5.92 mmol) in 125 mL of dichloromethane tetra-*n*-butylammonium fluoride trihydrate (4.67 g, 14.8 mmol, 2.5 equiv) was added. The solution was stirred for 2 d at ambient temperature without an inert gas atmosphere. The mixture was washed with three portions of water (each 75 mL). The organic layer was separated, dried over sodium sulfate and filtered prior to evaporation of all volatiles. The obtained residue was dissolved in refluxing hexane (20 mL) and stored at –70 °C overnight. The precipitate was filtered off and washed with cold pentane. Drying *in vacuo* yielded **1** (1.22 g, 3.32 mmol, 56% yield) as an off-white solid. Colorless single crystals were obtained by slow evaporation of a concentrated hexane solution at –30 °C. ^1H NMR (500 MHz, $CDCl_3$, 243 K): δ = 6.98 (s, 2H, $C^{Ar}H$), 6.81 (s, 2H, $C^{Ar}H$), 3.13 (s, 6H, $N(CH_3)_2$), 3.00 (s, 2H, C_qH), 2.57 (s, 6H, $N(CH_3)_2$), 2.17 (s, 6H, $C^{Ar}CH_3$), 1.32 (s, 6H, $C^{Ar}CH_3$) ppm. $^{13}C\{^1H\}$ NMR (126 MHz, $CDCl_3$, 243 K): δ = 148.1 (BC^{Ar}), 138.1 ($C^{Ar}CH_3$), 137.5 ($C^{Ar}CH_3$), 129.6 ($C^{Ar}H$), 128.8 ($C^{Ar}H$), 118.4 ($C^{Ar}C_q$), 84.8 ($C^{Ar}C_q$), 75.5 (C_qH), 44.5 ($N(CH_3)_2$), 40.5 ($N(CH_3)_2$), 22.3 ($C^{Ar}CH_3$), 20.1 ($C^{Ar}CH_3$) ppm. ^{11}B NMR (160 MHz, CD_2Cl_2, 243 K): δ = 49.0 (s) ppm. Solid-state IR: ν(C≡C–H) = 3280 cm^{-1}; ν(C≡C) = 2099 cm^{-1}. Solid-state Raman: ν(C≡C) = 2101 cm^{-1}. Elemental analysis for $[C_{24}H_{30}B_2N_2]$ (M_w = 368.26): calcd. C 78.30, H 8.21, N 7.61%; found C 78.40, H 8.65, N 7.27%. HRMS ASAP for $[C_{24}H_{31}B_2N_2]^+$ = $[M + H]^+$: calcd. 369.2668; found 369.2671.

$B_2(C_6H_4$-4-CCMe$)_2(NMe_2)_2$, 2

A solution of *t*-butyllithium (1.9 M, 5.53 mL, 10.5 mmol, 4.3 equiv) in pentane was added dropwise at 5 °C to a solution of 1-bromo-4-(propyn-1-yl)benzene (1.00 g, 5.13 mmol, 2.1 equiv.) in benzene (80 mL). The resulting solution was warmed to ambient temperature and stirred for 3 h. The resulting orange suspension was cooled to 5 °C and a solution of bis(dimethylamino)dichlorodiborane(4) (441 mg, 2.44 mmol) dissolved in benzene (10 mL) were added dropwise. The resulting suspension was warmed up and stirred at ambient temperature for 16 h prior to removal of all volatiles. The obtained residue was suspended in hexane (30 mL) and filtered through a 2 cm silica column. The resulting colorless solution was stored at –30 °C. The precipitate was filtered off and washed with cold pentane. Drying *in vacuo* yielded **2** (572 mg, 1.68 mmol, 69% yield) as a colorless solid. Colorless single crystals were obtained by slow evaporation of a concentrated hexane solution at –30 °C. ^{1}H NMR (500 MHz, CD_2Cl_2, 297 K): δ = 7.25 (d, ^3J = 8.07 Hz, 2H, $C^{Ar}H$), 7.06 (d, ^3J = 8.22 Hz, 2H, $C^{Ar}H$), 3.04 (s, 6H, $N(CH_3)_2$), 2.74 (s, 6H, $N(CH_3)_2$), 2.03 (s, 6H, C_qCH_3) ppm. $^{13}C\{^1H\}$ NMR (126 MHz, CD_2Cl_2, 297 K): δ = 145.9 (BC^{Ar}), 131.4 (C^{Ar}H), 130.8 (C^{Ar}H), 122.2 ($C^{Ar}C_q$), 85.5 (C_qCH_3), 80.4 ($C^{Ar}C_q$), 45.0 ($N(CH_3)_2$), 40.2 ($N(CH_3)_2$), 4.4 (C_qCH_3) ppm. ^{11}B NMR (160 MHz, CD_2Cl_2, 297 K): δ = 48.5 (s) ppm. Solid-state IR: *ν(C≡C) not detected.* Solid-state Raman: ν(C≡C) = 2255 cm^{-1}, 2219 cm^{-1}. Elemental analysis for [$C_{22}H_{26}B_2N_2$] (M_w = 340.08): calcd. C 77.70, H 7.71, N 8.24%; found C 76.66, H 7.84, N 7.94%. HRMS ASAP for $[C_{22}H_{26}B_2N_2]^+$ = $[M]^+$: calcd. 340.2277; found 340.2275.

B_2(2-C_4H_2S-5-(CCMe)$)_2(NMe_2)_2$, 3

A solution of *n*-butyllithium (2.5 M, 2.08 mL, 5.20 mmol, 2.3 equiv) in hexane was added dropwise at 5 °C to a solution of 2-bromo-5-(propyn-1-yl)thiophene (1.00 g, 4.75 mmol, 2.1 equiv.) in benzene (80 mL). The resulting solution was warmed up and stirred for 3 h at ambient temperature. The resulting yellow suspension was cooled to 5 °C and a solution of bis(dimethylamino)dichlorodiborane(4) (408 mg, 2.26 mmol) dissolved in benzene (10 mL) were added dropwise. The resulting suspension was warmed to ambient temperature and stirred for 2 h prior to removal of all volatiles. The obtained residue was suspended in hexane (30 mL) and filtered through a 2 cm silica column. The resulting yellow solution was stored at –30 °C. The precipitate was filtered off and washed with cold pentane. Drying *in vacuo* yielded **3** (620 mg, 1.76 mmol, 78% yield) as a colorless solid. Colorless single crystals were obtained by slow evaporation of a concentrated hexane solution at –30 °C. ^{1}H NMR (500 MHz, $CDCl_3$, 297 K): δ = 7.10 (d, ^3J = 3.54 Hz, 2H, $C^{Ar}H$), 6.98 (d, ^3J = 3.54 Hz, 2H, $C^{Ar}H$), 3.07 (s, 6H, $N(CH_3)_2$), 2.94 (s, 6H, $N(CH_3)_2$), 2.07 (s, 6H, C_qCH_3) ppm. $^{13}C\{^1H\}$ NMR (126 MHz, $CDCl_3$, 297 K): δ = 145.6 (BC^{Ar}), 134.9 (C^{Ar}H), 131.8 (C^{Ar}H), 128.2 ($C^{Ar}C_q$), 91.1 (C_qCH_3), 73.5 ($C^{Ar}C_q$), 45.6 ($N(CH_3)_2$), 41.0 ($N(CH_3)_2$), 4.9 (C_qCH_3) ppm.^{11}B NMR (160 MHz, $CDCl_3$, 297 K): δ = 43.1 (s) ppm. Solid-state IR: *ν(C≡C) not detected.* Solid-state Raman: ν(C≡C) = 2230 cm^{-1}. Elemental analysis for [$C_{18}H_{22}B_2N_2S_2$] (M_w = 352.13): calcd. C 61.40, H 6.30, N 7.96%; found C 61.14, H 6.39, N 7.75%. HRMS ASAP for $[C_{18}H_{23}B_2N_2S_2]^+$ = $[M + H]^+$: calcd. 353.1483; found 353.1475.

$B_2(C_4H_3S)_2(NMe_2)_2$, 4

A solution of *n*-butyllithium (2.5 M, 1.53 mL, 3.82 mmol, 2.3 equiv) in hexane was added dropwise at 5 °C to a solution of 2-bromothiophene (595 mg, 3.65 mmol, 2.2 equiv.) in benzene (30 mL). The resulting solution was warmed to ambient temperature and stirred for 3 h. The resulting colorless suspension was cooled to 5 °C and a solution of bis(dimethylamino)dichlorodiborane(4) (300 mg, 1.66 mmol) dissolved in benzene (5 mL) were added dropwise. The resulting suspension was warmed to ambient temperature and stirred for 2 h prior to removal of all volatiles. The obtained residue was suspended in hexane (30 mL) and filtered through a 2 cm silica column. The resulting yellow solution was stored at –30 °C and **4** was obtained as colorless solid and washed with cold pentane. Drying *in vacuo* yielded **10** (384 mg, 1.39 mmol, 84% yield) as a colorless solid. Colorless single crystals were obtained by slow evaporation of a concentrated hexane solution at –30 °C. ^{1}H NMR (500 MHz, CD_2Cl_2, 297 K): δ = 7.38 (dd, ^{4}J = 0.95 Hz, ^{3}J = 4.75 Hz, 2H, $C^{Ar}H$), 7.33 (dd, ^{4}J = 0.95 Hz, ^{3}J = 3.40 Hz, 2H, $C^{Ar}H$), 7.02 (dd, ^{3}J = 3.40 Hz, ^{3}J = 4.75 Hz, 2H, $C^{Ar}H$), 2.93 (s, 6H, $N(CH_3)_2$), 2.75 (s, 6H, $N(CH_3)_2$) ppm. $^{13}C\{^{1}H\}$ NMR (126 MHz, CD_2Cl_2, 297 K): δ = 144.5 (BC^{Ar}), 135.4 ($C^{Ar}H$), 130.0 ($C^{Ar}H$), 128.0 ($C^{Ar}H$), 45.7 ($N(CH_3)_2$), 40.9 ($N(CH_3)_2$) ppm. ^{11}B NMR (160 MHz, CD_2Cl_2, 297 K): δ = 44.1 (s) ppm. Elemental analysis for [$C_{12}H_{18}B_2N_2S_2$] (M_w = 276.03): calcd. C 52.22, H 6.57, N 10.15%; found C 52.90, H 6.71, N 10.15%. HRMS ASAP for $[C_{12}H_{18}B_2N_2S_2]^+$ = $[M]^+$: calcd. 276.1092; found 276.1093.

$B_2(C_6H_2$-2,6-Me_2-4-$(CHCHBMes_2))_2(NMe_2)_2$, 5a

Dimesitylborane (273 mg, 1.09 mmol, 2 equiv.) dissolved in benzene (5 mL) was added to a solution of **1** (200 mg, 543 µmol) in benzene (5 mL). The colorless solution was stirred for 2 h at ambient temperature prior to removal all volatiles. The obtained residue was suspended in hexane and filtered. The colorless residue was washed with cold pentane. Drying *in vacuo* yielded **5a** (336 mg, 387 µmol, 71% yield) as a colorless solid. ^{1}H NMR (500 MHz, CD_2Cl_2, 233 K): δ = 7.22 (d, ^{3}J = 17.67 Hz, 2H, BCH), 7.01 (s, 2H, $C^{Ar}H$), 6.97 (d, ^{3}J = 17.66 Hz, 2H, $C^{Ar}CH$), 6.86 (s, 2H, $C^{Ar}H$), 6.79 (s, 8H, $C^{Ar}H$), 3.09 (s, 6H, $N(CH_3)_2$), 2.52 (s, 6H, $N(CH_3)_2$), 2.25 (s, 12H, $C^{Ar}CH_3$), 2.17 (s, 6H, $C^{Ar}CH_3$), 2.13 (s, 24H, $C^{Ar}CH_3$), 1.41 (s, 6H, $C^{Ar}CH_3$) ppm. $^{13}C\{^{1}H\}$ NMR (126 MHz, CD_2Cl_2, 233 K): δ = 154.7 ($C^{Ar}CH$), 150.2 (BC^{Ar}), 142.1 (BC^{Ar}), 140.2 ($C^{Ar}CH_3$), 138.1 ($C^{Ar}CH_3$), 138.1 ($C^{Ar}CH_3$),138.0 ($C^{Ar}CH_3$), 134.7 (CHC_q), 134.4 (BCH), 127.8 ($C^{Ar}H$), 125.7 ($C^{Ar}H$), 125.6 ($C^{Ar}H$), 44.2 ($N(CH_3)_2$), 40.1 ($N(CH_3)_2$), 23.0 ($C^{Ar}CH_3$), 22.2 ($C^{Ar}CH_3$), 21.0 ($C^{Ar}CH_3$), 20.1 ($C^{Ar}CH_3$) ppm. ^{11}B NMR (129 MHz, $CDCl_3$, 297 K): δ = 73.1 (br s, *B*), 50.2 (s, B_2) ppm. Solid-state IR: ν(C=C) = 1585 cm^{-1}. Solid-state Raman: ν(C=C) = 1593 cm^{-1}. Elemental analysis for [$C_{60}H_{76}B_4N_2$] (M_w = 868.52): calcd. C 82.98, H 8.82, N 3.23%; found C 82.59, H 8.86, N 3.20%. HRMS LIFDI for $[C_{60}H_{76}B_4N_2]^+$ = $[M]^+$: calcd. 868.6375; found 868.6375.

Oligomerization product of 1, 5b

Mesitylborane (65.0 mg, 489 µmol) dissolved in benzene (2 mL) was added dropwise to a solution of **1** (180 mg, 489 µmol) in benzene (5 mL). The colorless solution was stirred for 1 h at ambient temperature prior to removal of all volatiles. The obtained residue was suspended in hexane and filtered. The colorless residue was washed with hexane. Drying *in vacuo* yielded **5b** (193 mg, 79% yield) as a colorless solid. ^{1}H NMR (500 MHz, CD_2Cl_2, 297 K): δ = 7.30 - 6.60 (m, 10H, $C^{Ar}H$, C*H*), 3.14 (br s, 6H, $N(CH_3)_2$), 2.57 (br s, 6H, $N(CH_3)_2$), 2.40 - 1.10 (m, 21H, $C^{Ar}CH_3$) ppm. ^{11}B NMR: *not detected*. Solid-state IR: ν(C=C) = 1587 cm^{-1}. Solid-state Raman: ν(C=C) = 1595 cm^{-1}.

Oligomerization product of 1, **5c**

Durylborane (175 mg, 1.20 mmol) dissolved in benzene (2 mL) was added dropwise to a solution of **1** (442 mg, 1.20 mmol) in benzene (5 mL). The colorless solution was stirred for 1 h at ambient temperature prior to removal of all volatiles. The obtained residue was suspended in hexane and filtered. The colorless residue was washed with hexane. Drying *in vacuo* yielded **5c** (582 mg, 94% yield) as a colorless solid. ^{1}H NMR (500 MHz, CD_2Cl_2, 297 K): δ = 7.30 - 6.40 (m, 9H, $C^{Ar}H$, C*H*), 3.16 (br s, 6H, $N(CH_3)_2$), 2.59 (br s, 6H, $N(CH_3)_2$), 2.40 - 1.10 (m, 24H, $C^{Ar}CH_3$) ppm. ^{11}B NMR: *not detected*. Solid-state IR: ν(C=C) = 1586 cm^{-1}. Solid-state Raman: ν(C=C) = 1595 cm^{-1}.

$B_2(C_6H_4$-4-$(CHCMeBMes_2))_2(NMe_2)_2$, 6a

Dimesitylborane (295 mg, 1.18 mmol, 2 equiv.) dissolved in benzene (5 mL) was added to a solution of **2** (200 mg, 588 µmol) in benzene (5 mL). The colorless solution was stirred for 5 h at 75 °C prior to removal of all volatiles. The obtained residue was suspended in hexane, filtered and the resulting colorless solution was stored at –30 °C. The precipitate was filtered off and washed with cold pentane. Drying *in vacuo* yielded **6a** (302 mg, 359 µmol, 61% yield) as a colorless solid. Colorless single crystals were obtained by slow evaporation of a concentrated hexane solution at –30 °C. ^{1}H NMR (500 MHz, $CDCl_3$, 297 K): δ = 7.32 (d, ^{3}J = 7.94 Hz, 2H, $C^{Ar}H$), 7.18 (d, ^{3}J = 8.02 Hz, 2H, $C^{Ar}H$), 6.94 (d, ^{4}J = 1.50 Hz, 2H, $C^{Ar}CH$), 6.79 (s, 8H, $C^{Ar}H$), 3.04 (s, 6H, $N(CH_3)_2$), 2.75 (s, 6H, $N(CH_3)_2$), 2.28 (s, 12H, $C^{Ar}CH_3$), 2.16 (s, 24H, $C^{Ar}CH_3$), 2.12 (d, ^{4}J = 1.53 Hz, 6H, C_qCH_3) ppm. $^{13}C\{^{1}H\}$ NMR (126 MHz, $CDCl_3$, 297 K): δ = 149.2 (BC_q), 145.9 (BC^{Ar}), 145.4 (C_qCH), 142.3 (BC^{Ar}), 140.6 ($C^{Ar}CH_3$), 138.2 ($C^{Ar}CH_3$), 136.5 (C_qCH), 131.2 ($C^{Ar}H$),129.1 ($C^{Ar}H$), 128.2 ($C^{Ar}H$), 44.9 ($N(CH_3)_2$), 40.2 ($N(CH_3)_2$), 23.0 ($C^{Ar}CH_3$), 21.3 ($C^{Ar}CH_3$), 18.1 (C_qCH_3) ppm.^{11}B NMR (129 MHz, C_6D_6, 297 K): δ = 75.9 (br s, *B*), 49.0 (s, B_2) ppm. Solid-state IR: ν(C=C) = 1580 cm^{-1}. Solid-state Raman: ν(C=C) = 1598 cm^{-1}, 1583 cm^{-1}. Elemental analysis for [$C_{58}H_{72}B_4N_2$] (M_w = 840.47): calcd. C 82.89, H 8.64, N 3.33%; found C 82.59, H 8.74, N 3.07%. HRMS LIFDI for $[C_{58}H_{72}B_4N_2]^+$ = $[M]^+$: calcd. 840.6062; found 840.6056.

Oligomerization product of 2, **6b**

Mesitylborane (78.0 mg, 588 µmol) dissolved in benzene (2 mL) was added dropwise to a solution of **2** (200 mg, 588 µmol) in benzene (5 mL). The colorless solution was stirred for 1 h at ambient temperature

prior to removal of all volatiles. The obtained residue was suspended in hexane and filtered. The pale orange residue was washed with hexane. Drying *in vacuo* yielded **6b** (83.0 mg, 30% yield) as a pale orange solid. ^{1}H NMR (400 MHz, CD_2Cl_2, 297 K): δ = 7.60 - 7.00 (m, 10H, $C^{Ar}H$, CH), 6.80 (br s, 2H, $C^{Ar}H$), 3.06 (br s, 6H, $N(CH_3)_2$), 2.77 (br s, 6H, $N(CH_3)_2$), 2.30 - 2.00 (m, 15H, C_qCH_3, $C^{Ar}CH_3$) ppm. ^{11}B NMR: *not detected*. Solid-state IR: ν(C=C) = 1580 cm^{-1}. Solid-state Raman: ν(C=C) = 1599 cm^{-1}.

Oligomerization product of 2, 6c

Durylborane (86.0 mg, 588 µmol) dissolved in benzene (2 mL) was added dropwise to a solution of **2** (200 mg, 588 µmol) in benzene (5 mL). The colorless solution was stirred for 1 h at ambient temperature prior to removing of all volatiles. The obtained residue was suspended in hexane and filtered. The pale orange residue was washed with hexane. Drying *in vacuo* yielded **6c** (187 mg, 65% yield) as a pale orange solid. ^{1}H NMR (400 MHz, CD_2Cl_2, 297 K): δ = 7.50 – 6.70 (m, 11H, $C^{Ar}H$, CH_2), 3.05 (br s, 6H, $N(CH_3)_2$), 2.76 (br s, 6H, $N(CH_3)_2$), 2.30 – 1.70 (m, 18H, C_qCH_3, $C^{Ar}CH_3$) ppm. ^{11}B NMR: *not detected*. Solid-state IR: ν(C=C) = 1580 cm^{-1}. Solid-state Raman: ν(C=C) = 1598 cm^{-1}.

$B_2(2\text{-}C_4H_2S\text{-}5\text{-}(CHCMeBMes_2))_2(NMe_2)_2$, 7a

Dimesitylborane (285 mg, 1.14 mmol, 2 equiv.) dissolved in benzene (5 mL) was added to a solution of **3** (200 mg, 568 µmol) in benzene (5 mL). The yellow solution was stirred for 3 h at 75 °C prior to removal of all volatiles. The obtained residue was suspended in hexane, filtered and the resulting yellow solution was stored at –30 °C. The precipitate was filtered off and washed with cold pentane. Drying *in vacuo* yielded **7** (356 mg, 418 µmol, 74% yield) as a pale yellow solid. ^{1}H NMR (500 MHz, C_6D_6, 297 K): δ = 7.63 (m, 2H, $C^{Ar}CH$), 7.19 (d, ^{3}J = 3.65 Hz, 2H, $C^{Ar}H$), 6.94 (d, ^{3}J = 3.65 Hz, 2H, $C^{Ar}H$), 6.78 (s, 8H, $C^{Ar}H$), 2.82 (s, 6H, $N(CH_3)_2$), 2.69 (s, 6H, $N(CH_3)_2$), 2.43 (d, ^{4}J = 1.35 Hz, 6H, C_qCH_3), 2.26 (s, 24H, $C^{Ar}CH_3$), 2.17 (s, 12H, $C^{Ar}CH_3$) ppm. $^{13}C\{^{1}H\}$ NMR (126 MHz, C_6D_6, 297 K): δ = 148.4 (BC^{Ar}), 147.2 (BC_q), 146.8 (C^{Ar}CH), 142.7 (BC^{Ar}), 140.8 ($C^{Ar}CH_3$), 139.2 (C^{Ar}CH), 138.5 ($C^{Ar}CH_3$), 136.0 (C^{Ar}H), 132.8 (C^{Ar}H), 128.8 (C^{Ar}H), 45.3 ($N(CH_3)_2$), 40.9 ($N(CH_3)_2$), 23.3 ($C^{Ar}CH_3$), 21.3 ($C^{Ar}CH_3$), 18.9 (C_qCH_3) ppm. ^{11}B NMR (160 MHz, C_6D_6, 297 K): δ = 75.1 (br s, *B*), 44.4 (br s, B_2) ppm. Solid-state IR: ν(C=C) = 1564 cm^{-1}. Solid-state Raman: ν(C=C) = 1567 cm^{-1}. Elemental analysis for [$C_{54}H_{68}B_4N_2S_2$] (M_w = 852.52): calcd. C 76.08, H 8.04, N 3.29%; found C 75.84, H 7.80, N 3.11%. HRMS LIFDI for $[C_{54}H_{68}B_4N_2S_2]^+ = [M]^+$: calcd. 852.5191; found 852.5191.

Oligomerization product of 3, 7b

Mesitylborane (75.0 mg, 568 µmol) dissolved in benzene (2 mL) was added dropwise to a solution of **3** (200 mg, 568 µmol) in benzene (5 mL). The yellow solution was stirred for 1 h at ambient temperature prior to removing of all volatiles. The obtained residue was suspended in hexane and filtered. The green residue was washed with hexane. Drying *in vacuo* yielded **7b** (120 mg, 44% yield) as a green solid. ^{1}H NMR (400 MHz, $CDCl_3$, 297 K): δ = 7.20 - 6.60 (m, 8H, $C^{Ar}H$, CH), 3.14 (br s, 6H, $N(CH_3)_2$), 2.57 (br

s, 6H, N(CH_3)$_2$), 2.40 - 1.70 (m, 15H, C$_q$CH_3, C^{Ar}CH_3) ppm. ^{11}B NMR: *not detected*. Solid-state IR: ν(C=C) = 1563 cm^{-1}. Solid-state Raman: ν(C=C) = 1568 cm^{-1}.

Oligomerization product of 3, 7c

Durylborane (104 mg, 710 µmol) dissolved in benzene (2 mL) was added dropwise to a solution of **3** (250 mg, 710 µmol) in benzene (5 mL). The yellow solution was stirred for 1 h at ambient temperature prior to removing of all volatiles. The obtained residue was suspended in hexane and filtered. The green residue was washed with hexane. Drying *in vacuo* yielded **7c** (200 mg, 56% yield) as a green solid. ^{1}H NMR (400 MHz, CD_2Cl_2, 297 K): δ = 7.40 - 6.50 (m, 8H, C^{Ar}*H*, C*H*), 3.20 - 2.70 (m, 12H, N(CH_3)$_2$), 2.40 - 1.70 (m, 18H, C$_q$CH_3, C^{Ar}CH_3) ppm. ^{11}B NMR: *not detected*. Solid-state IR: ν(C=C) = 1564 cm^{-1}. Solid-state Raman: ν(C=C) = 1568 cm^{-1}.

HBMes(NMe$_2$), 8

Bis(dimethylamino)mesitylborane (20.0 mg, 91.0 µmol) was added to a solution of mesitylborane (12.2 mg, 91.0 µmol) in benzene (1 mL). The colorless solution was stirred for 1 h at 60 °C prior to removal of all volatiles. Without further purification **8** (28.0 mg, 160 µmol, 88% yield) was obtained as a colorless liquid. ^{1}H NMR (500 MHz, C_6D_6, 297 K): δ = 6.83 (m, 2H, C^{Ar}*H*), 5.41 (m, 1H, B*H*), 2.76 (s, 3H, N(CH_3)$_2$), 2.36 (s, 3H, N(CH_3)$_2$), 2.25 (s, 6H, C^{Ar}CH_3), 2.23 (s, 3H, C^{Ar}CH_3) ppm. ^{13}C{^{1}H} NMR (126 MHz, C_6D_6, 297 K): δ = 139.0 (*C*^{Ar}CH$_3$), 136.8 (*C*^{Ar}CH$_3$), 127.5 (*C*^{Ar}H), 44.6 (N(*C*H$_3$)$_2$), 38.6 (N(*C*H$_3$)$_2$), 21.7 (C^{Ar}*C*H$_3$), 21.3 (C^{Ar}*C*H$_3$) ppm. ^{11}B NMR (160 MHz, C_6D_6, 297 K): δ = 41.5 (d, $^1J_{B\text{-}H}$ = 109.3 Hz) ppm. Solid-state IR: ν(B–H) = 2445 cm^{-1}. HRMS ASAP for $[C_{11}H_{19}BN]^+$ = $[M + H]^+$: calcd. 176.1605; found 176.1600.

(Me$_2$N)BCat, 9

Catecholborane (13.0 mg, 109 µmol, 2 equiv.) was added to a solution of **1** (20.0 mg, 54.3 µmol) in benzene (1 mL). The colorless solution was stirred for 1 d at 80 °C prior to removal of all volatiles. The residue was dissolved in hexane (2 mL) and the resulting colorless solution was stored at –30 °C. **9** was obtained as a colorless solid and washed with cold pentane. Drying *in vacuo* yielded **9** (6.55 mg, 40.2 µmol, 74% yield) as a colorless solid. Colorless single crystals were obtained by slow evaporation of a concentrated hexane solution at –30 °C. *Note: The NMR data are in line with previously reported data.*[7]

B$_2$(C$_6$H$_2$-2,6-Me$_2$-4-(CHCHBCat))$_2$(NMe$_2$)$_2$, 10

Catecholborane (71.0 mg, 590 µmol, 2.2 equiv.) was added to a solution of **1** (100 mg, 268 µmol) and Wilkinson's catalyst (2.50 mg, 2.68 µmol, 0.01 equiv.) in benzene (10 mL). The colorless solution was stirred for 5 d at ambient temperature prior to removing of all volatiles. The obtained residue was suspended in hexane, filtered and the resulting colorless solution was stored at –30 °C. The precipitate

was filtered off and washed with cold pentane. Drying *in vacuo* yielded **10** (136 mg, 225 µmol, 84% yield) as a colorless solid. ^{1}H NMR (500 MHz, CD_2Cl_2, 243 K): δ = 7.62 (d, ^{4}J = 18.46 Hz, 2H, BC*H*), 7.25 (m, 4H, $C^{Ar}H$), 7.11 (s, 2H, $C^{Ar}H$), 7.09 (m, 4H, $C^{Ar}H$), 6.93 (s, 2H, $C^{Ar}H$), 6.38 (d, ^{4}J = 18.47 Hz, 2H, $C^{Ar}CH$), 3.14 (s, 6H, $N(CH_3)_2$), 2.58 (s, 6H, $N(CH_3)_2$), 2.25 (s, 12H, $C^{Ar}CH_3$), 1.43 (s, 6H, $C^{Ar}CH_3$) ppm. $^{13}C\{^{1}H\}$ NMR (126 MHz, CD_2Cl_2, 243 K): δ = 153.0 ($C^{Ar}C$H), 149.9 (BC^{Ar}), 148.2 (OC^{Ar}), 138.3 ($C^{Ar}CH_3$), 138.2 ($C^{Ar}CH_3$), 134.1 ($C^{Ar}C_q$), 125.1 (C^{Ar}H), 124.7 (C^{Ar}H), 122.6 (C^{Ar}H), 112.2 (C^{Ar}H), 109.9 (B*C*H), 44.3 ($N(CH_3)_2$), 40.3 ($N(CH_3)_2$), 22.3 ($C^{Ar}CH_3$), 20.0 ($C^{Ar}CH_3$) ppm. ^{11}B NMR (129 MHz, CD_2Cl_2, 297 K): δ = 50.0 (br s, B_2), 32.0 (s, *B*) ppm. Solid-state IR: ν(C=C) = 1548 cm^{-1}. Solid-state Raman: ν(C=C) = 1621, 1598 cm^{-1}. HRMS LIFDI for $[C_{36}H_{40}B_4N_2O_4]^+$ = $[M]^+$: calcd. 608.3355; found 608.3349.

NMR spectra of isolated compounds

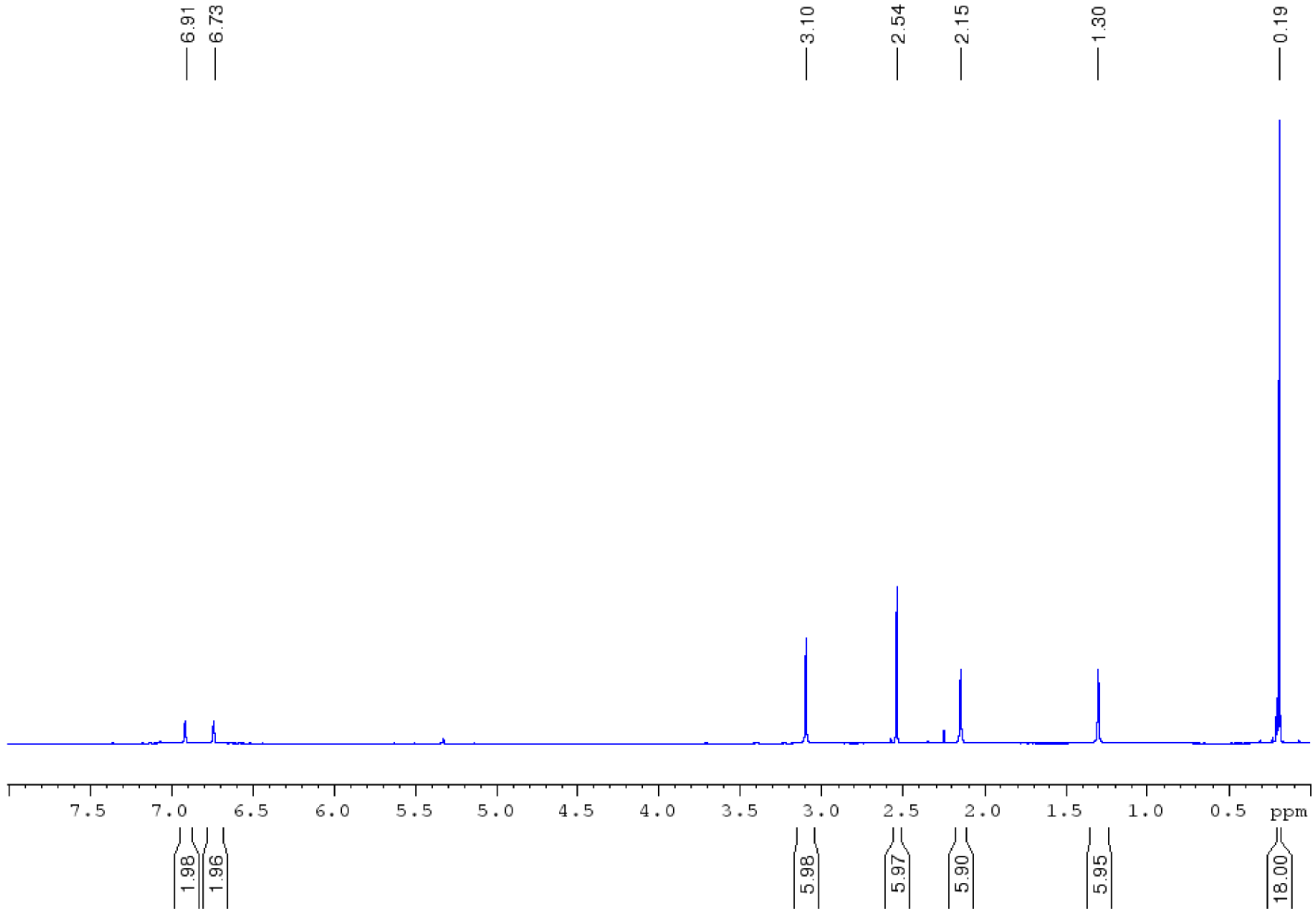

Figure S1. ^{1}H NMR spectrum of **$B_2(C_6H_2$-2,6-Me_2-4-$(CCSiMe_3))_2(NMe_2)_2$**, **1**$^{\mathbf{TMS}}$ in CD_2Cl_2.

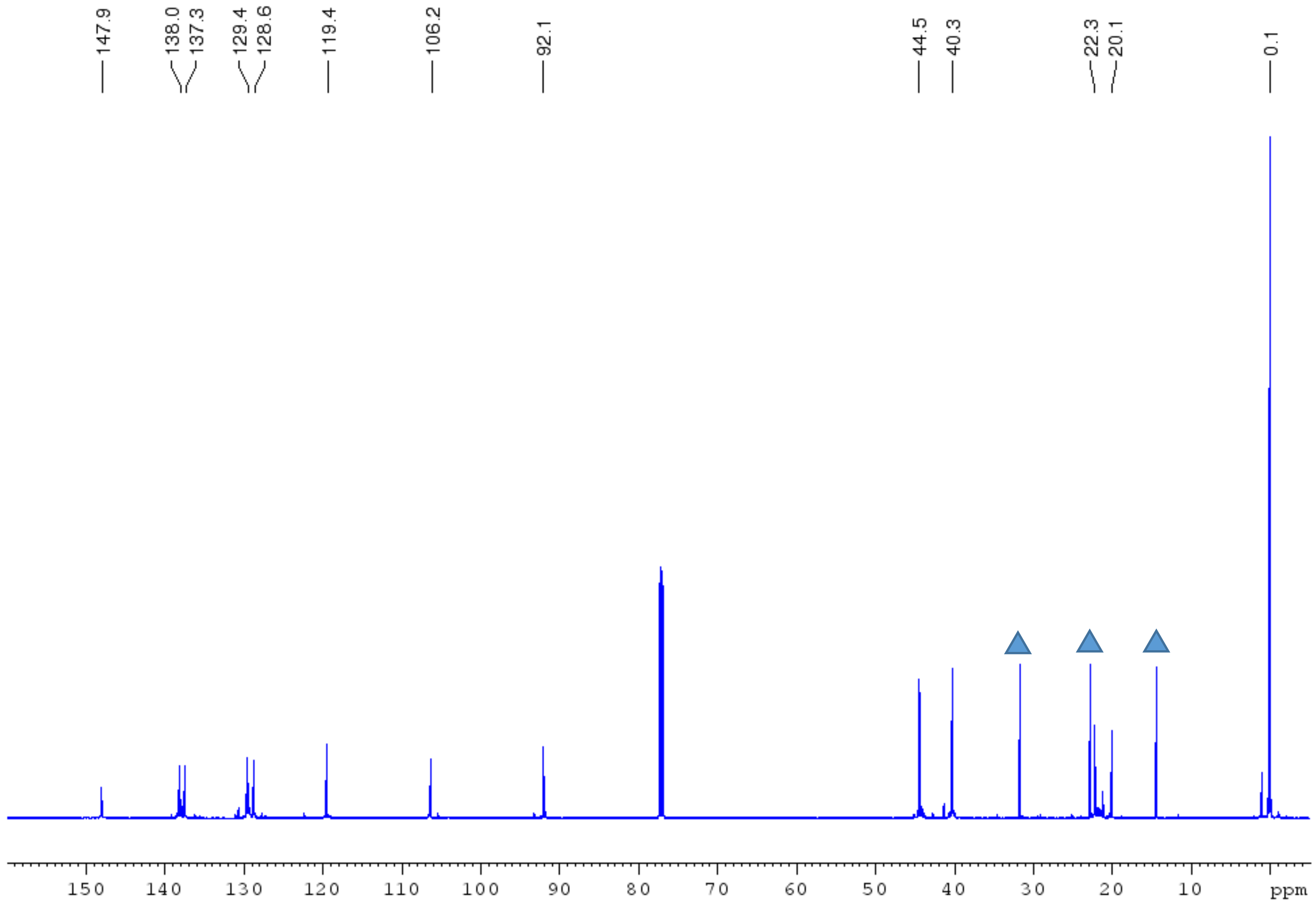

Figure S2. $^{13}C\{^{1}H\}$ NMR spectrum of **$B_2(C_6H_2$-2,6-Me_2-4-$(CCSiMe_3))_2(NMe_2)_2$**, **1**^{TMS} in $CDCl_3$. The resonances marked with △ correspond to residual hexane.

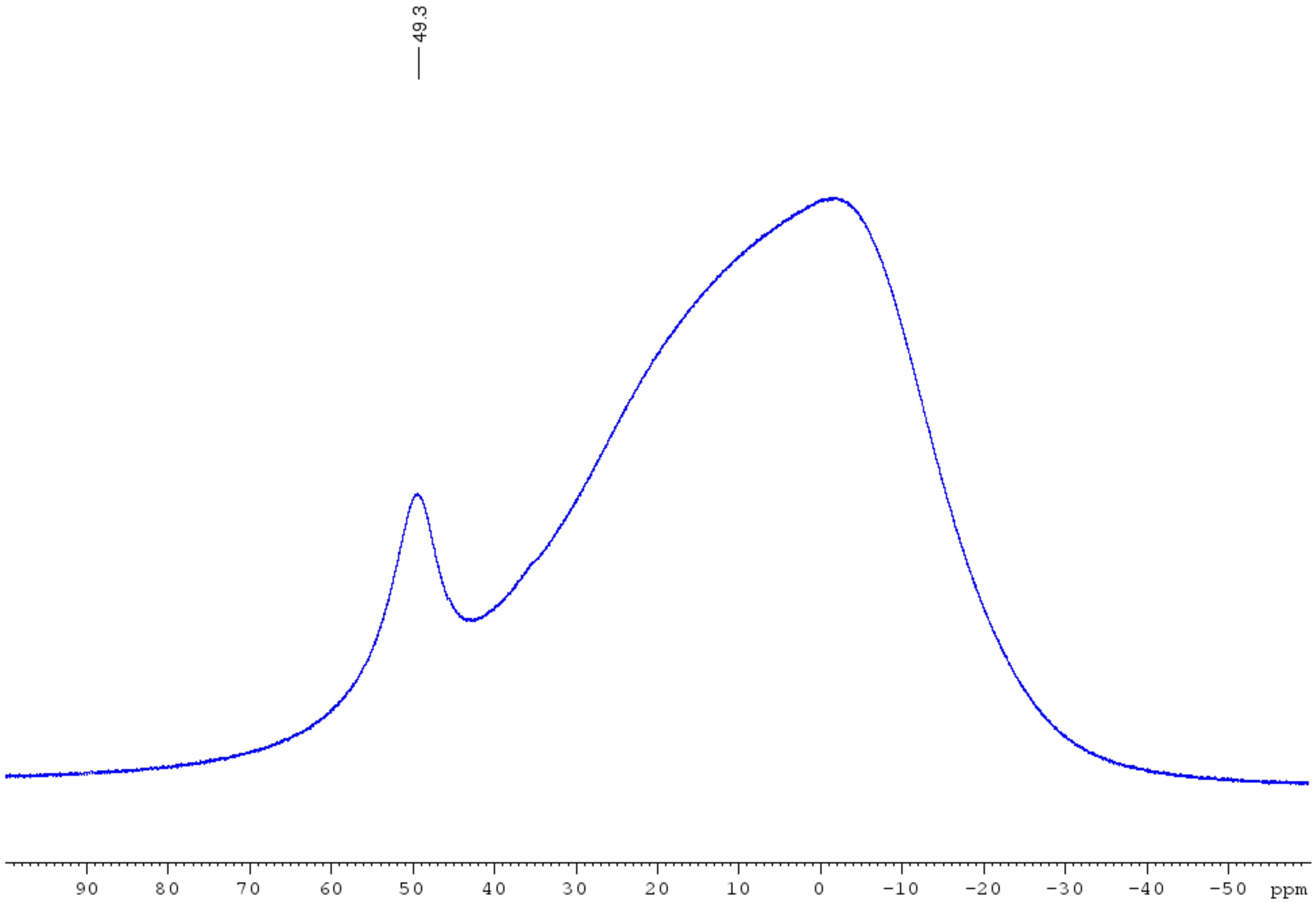

Figure S3. ^{11}B NMR spectrum of **$B_2(C_6H_2$-2,6-Me_2-4-$(CCSiMe_3))_2(NMe_2)_2$, 1^{TMS}** in CD_2Cl_2.

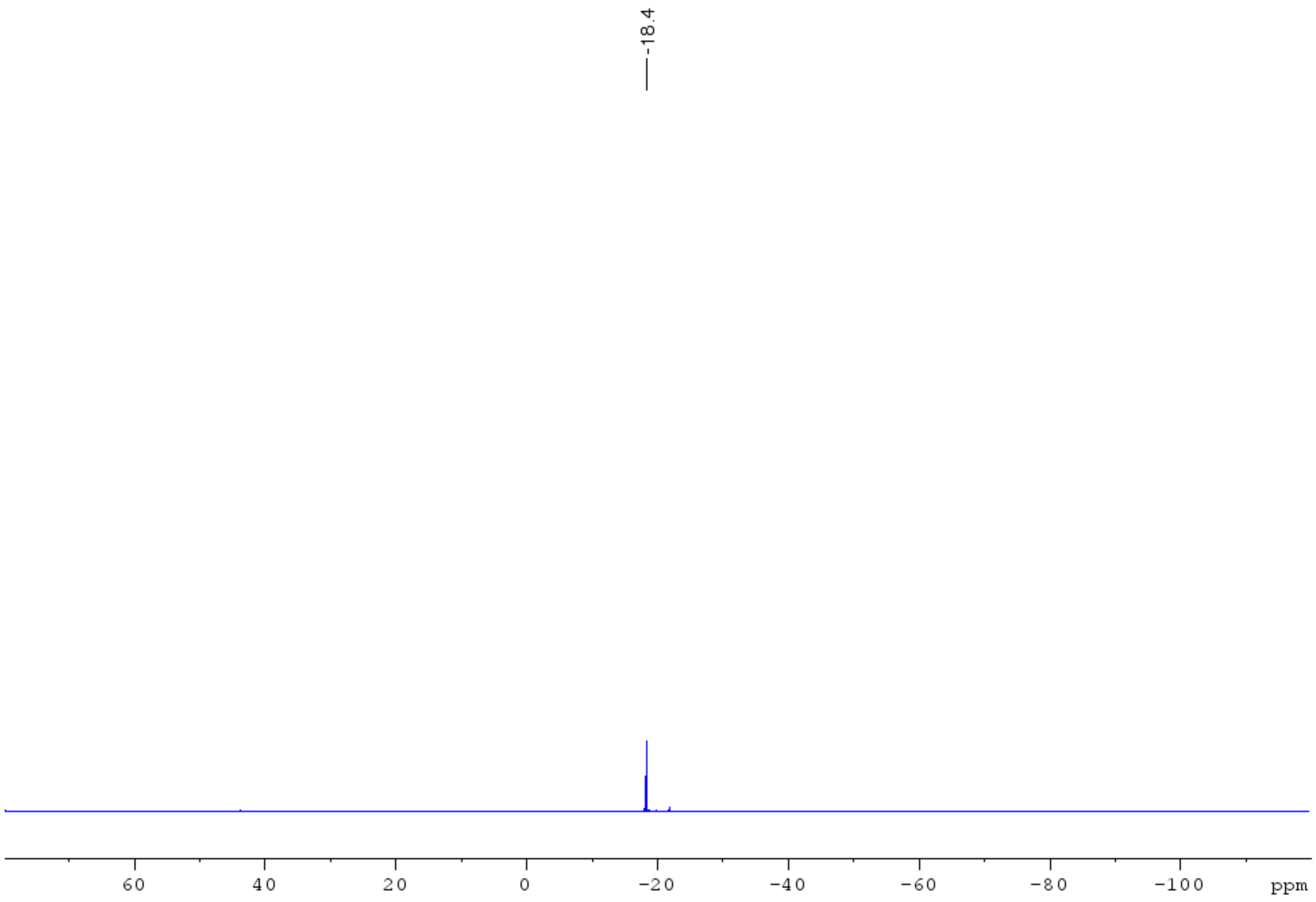

Figure S4. ^{29}Si NMR spectrum of **$B_2(C_6H_2$-2,6-Me_2-4-$(CCSiMe_3))_2(NMe_2)_2$**, **1^{TMS}** in $CDCl_3$

.

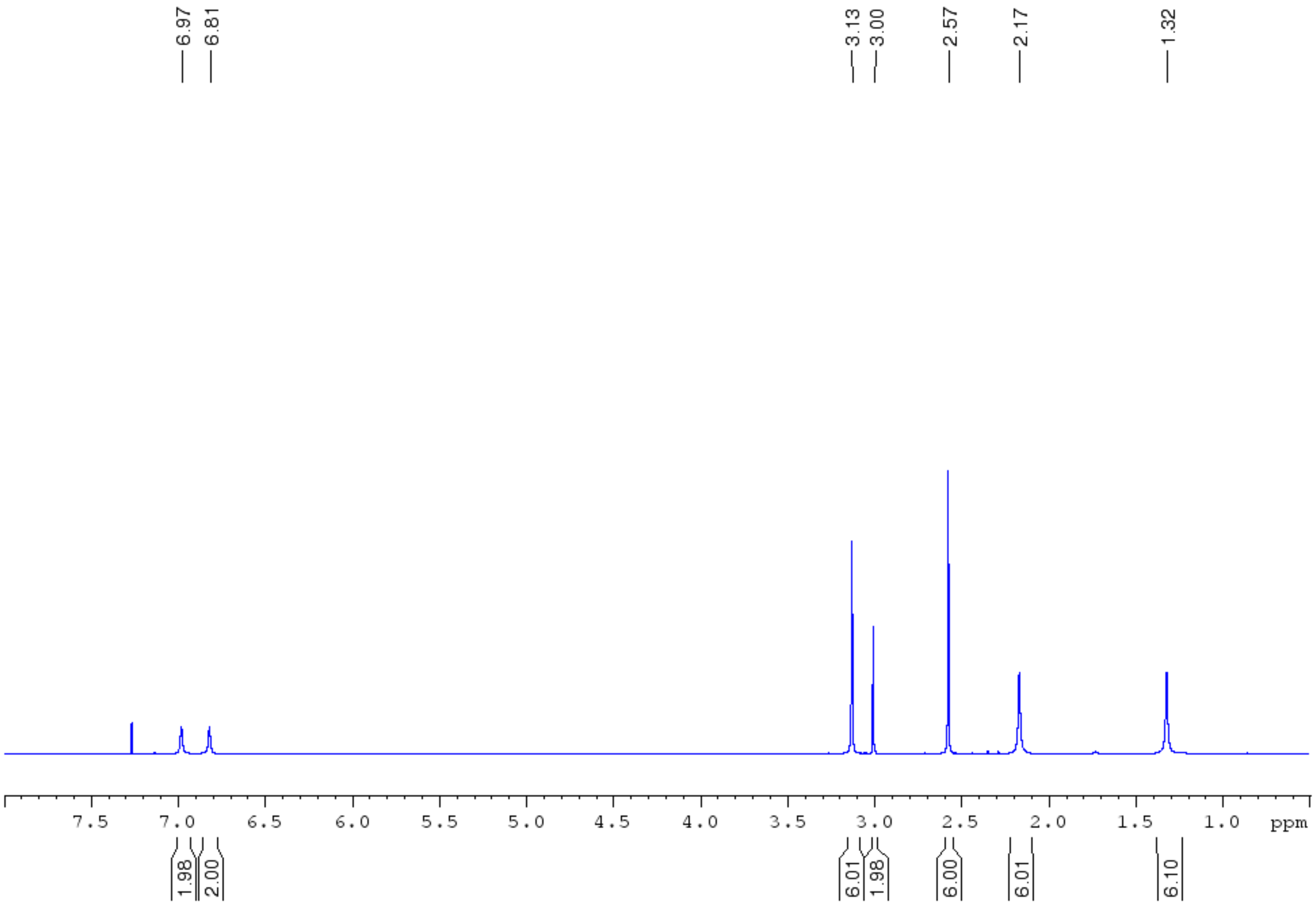

Figure S5. ^{1}H NMR spectrum of **$B_2(C_6H_2$-2,6-Me_2-4-$(CCH))_2(NMe_2)_2$**, **1** in $CDCl_3$.

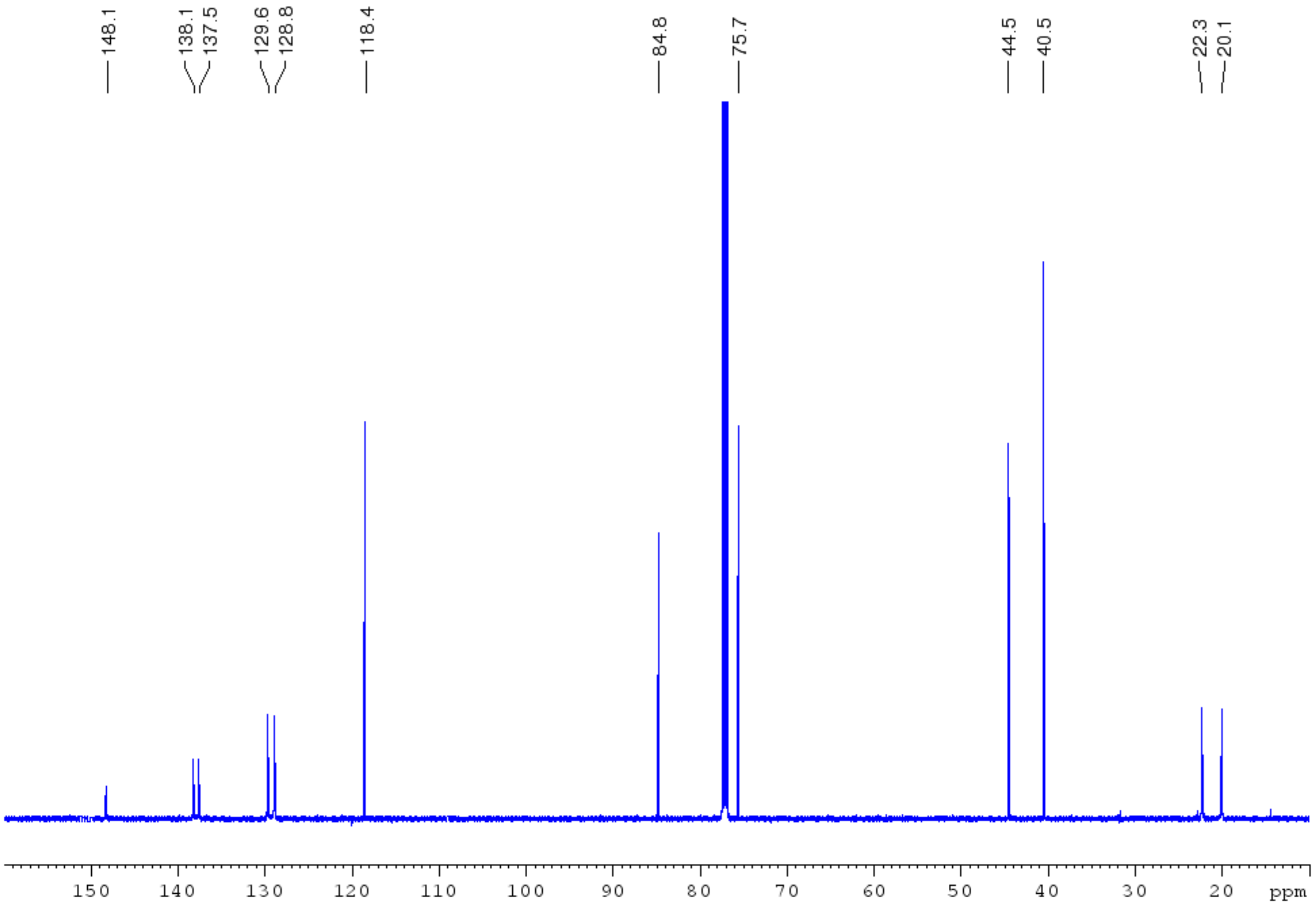

Figure S6. $^{13}C\{^{1}H\}$ NMR spectrum of **$B_2(C_6H_2$-2,6-Me_2-4-$(CCH))_2(NMe_2)_2$**, **1** in $CDCl_3$.

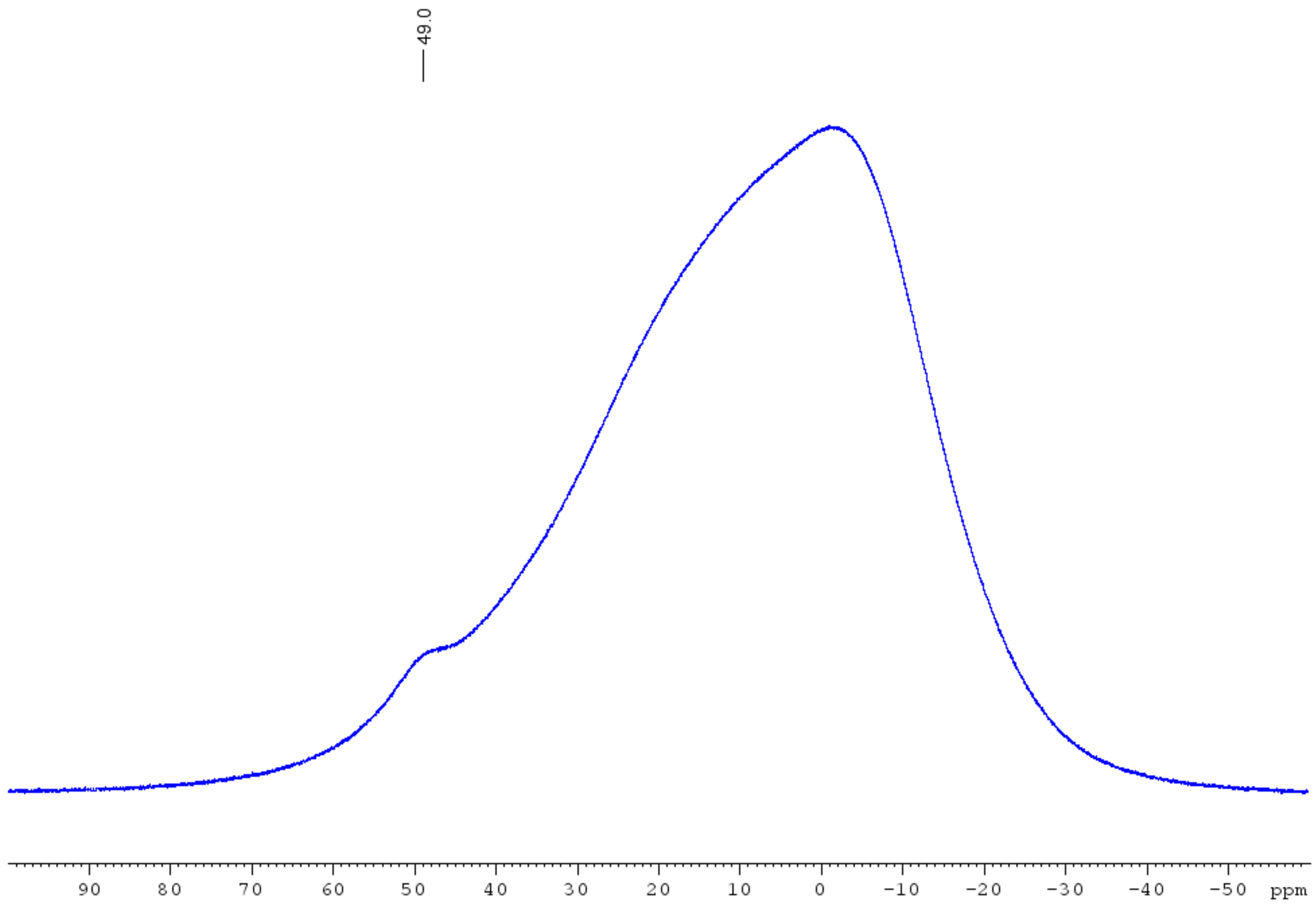

Figure S7. ^{11}B NMR spectrum of of **$B_2(C_6H_2$-2,6-Me_2-4-$(CCH))_2(NMe_2)_2$**, **1** in CD_2Cl_2.

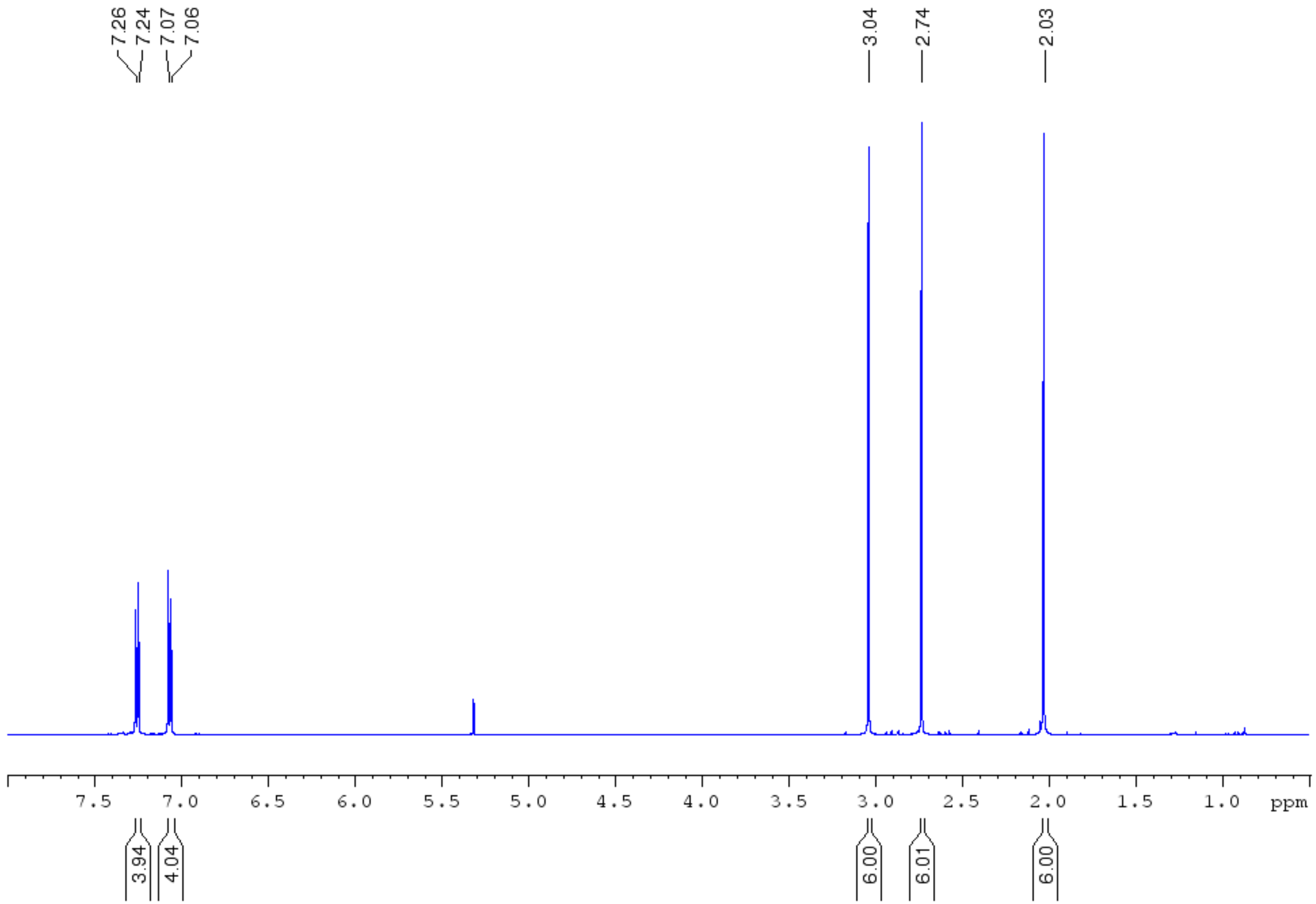

Figure S8. ^{1}H NMR spectrum of **$B_2(C_6H_4$-4-CCMe$))_2(NMe_2)_2$**, **2** in CD_2Cl_2.

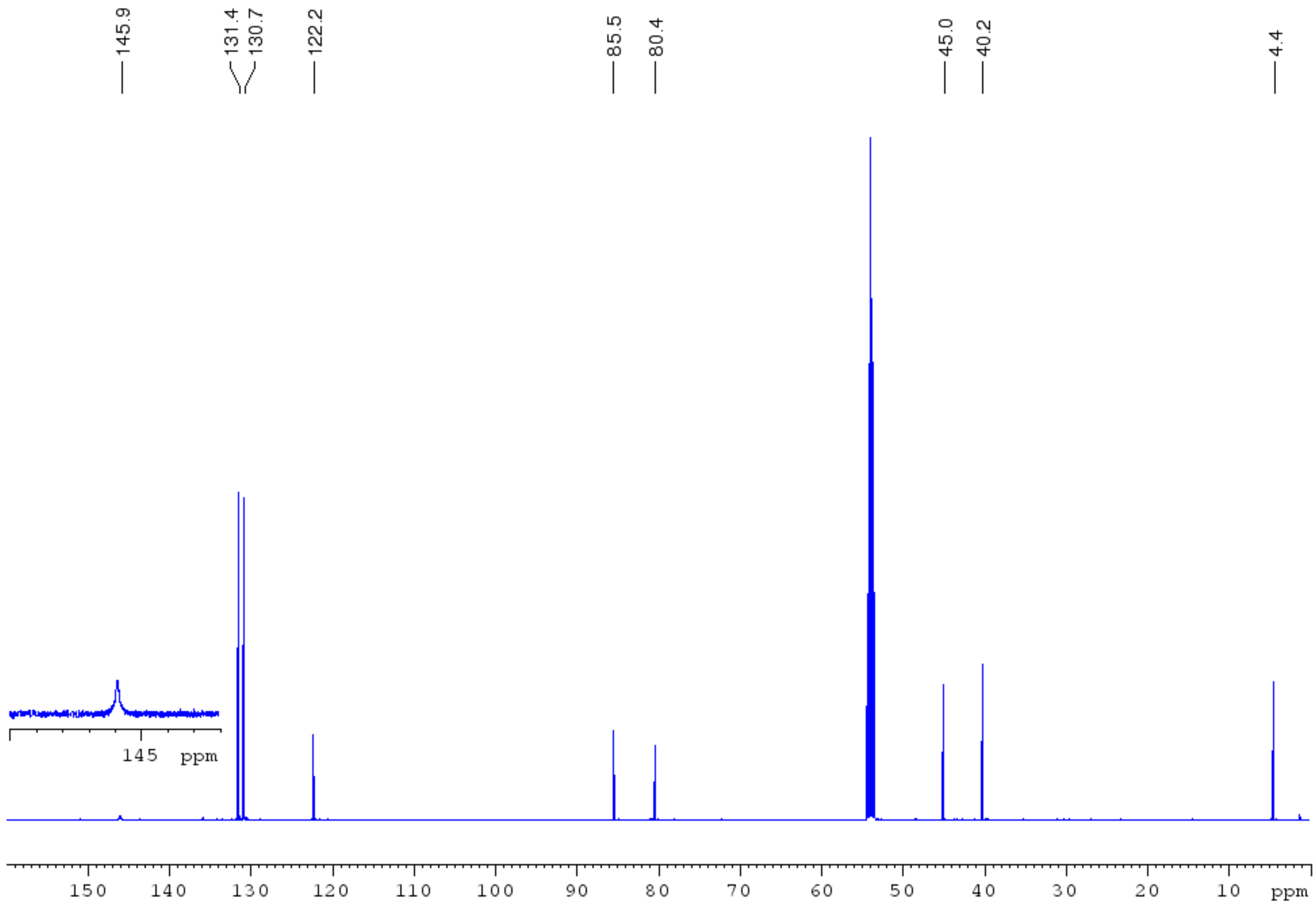

Figure S9. $^{13}C\{^1H\}$ spectrum of **$B_2(C_6H_4$-4-CCMe$))_2(NMe_2)_2$**, **2** in CD_2Cl_2.

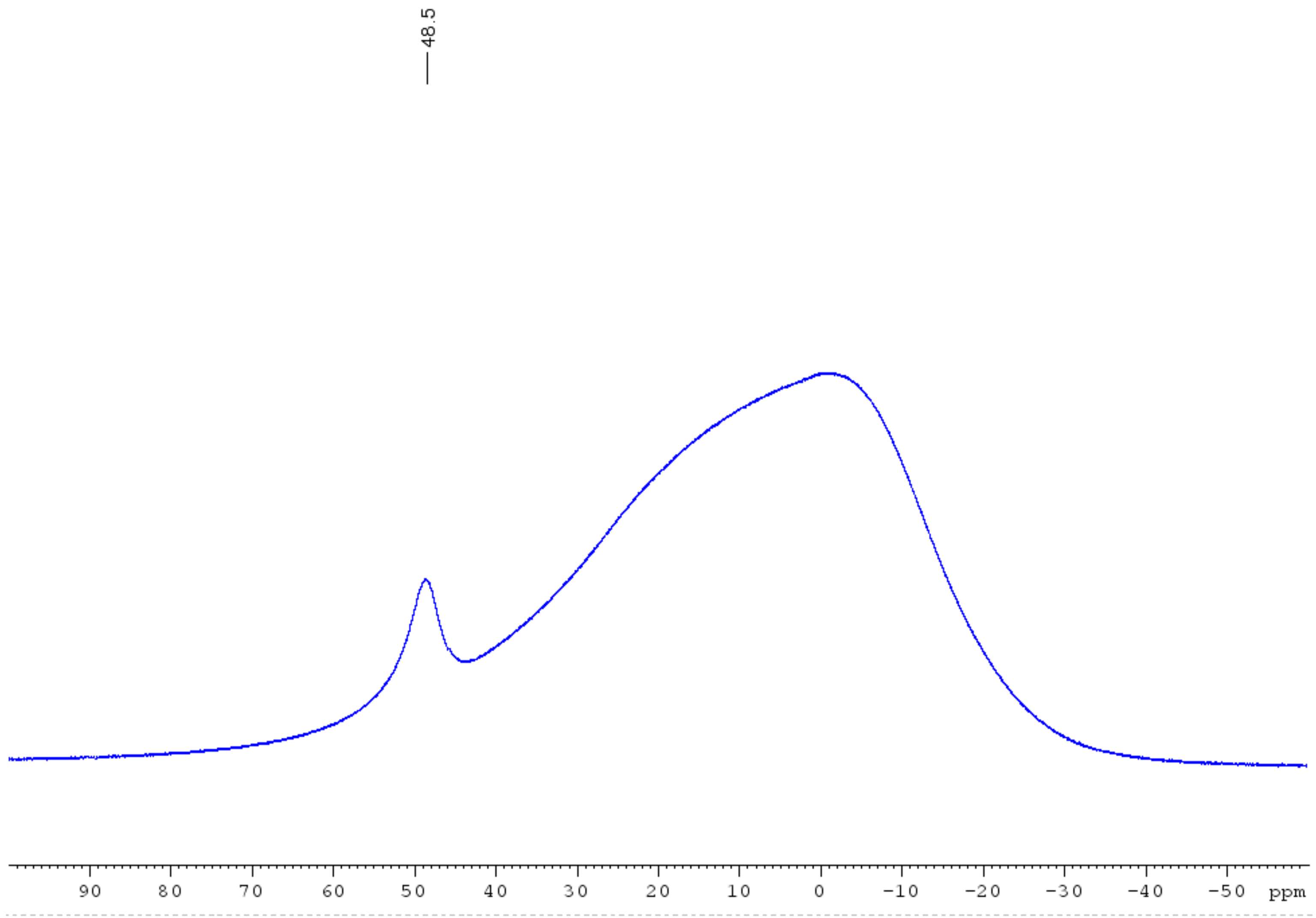

Figure S10. ^{11}B spectrum of **$B_2(C_6H_4$-4-CCMe$))_2(NMe_2)_2$, 2** in CD_2Cl_2.

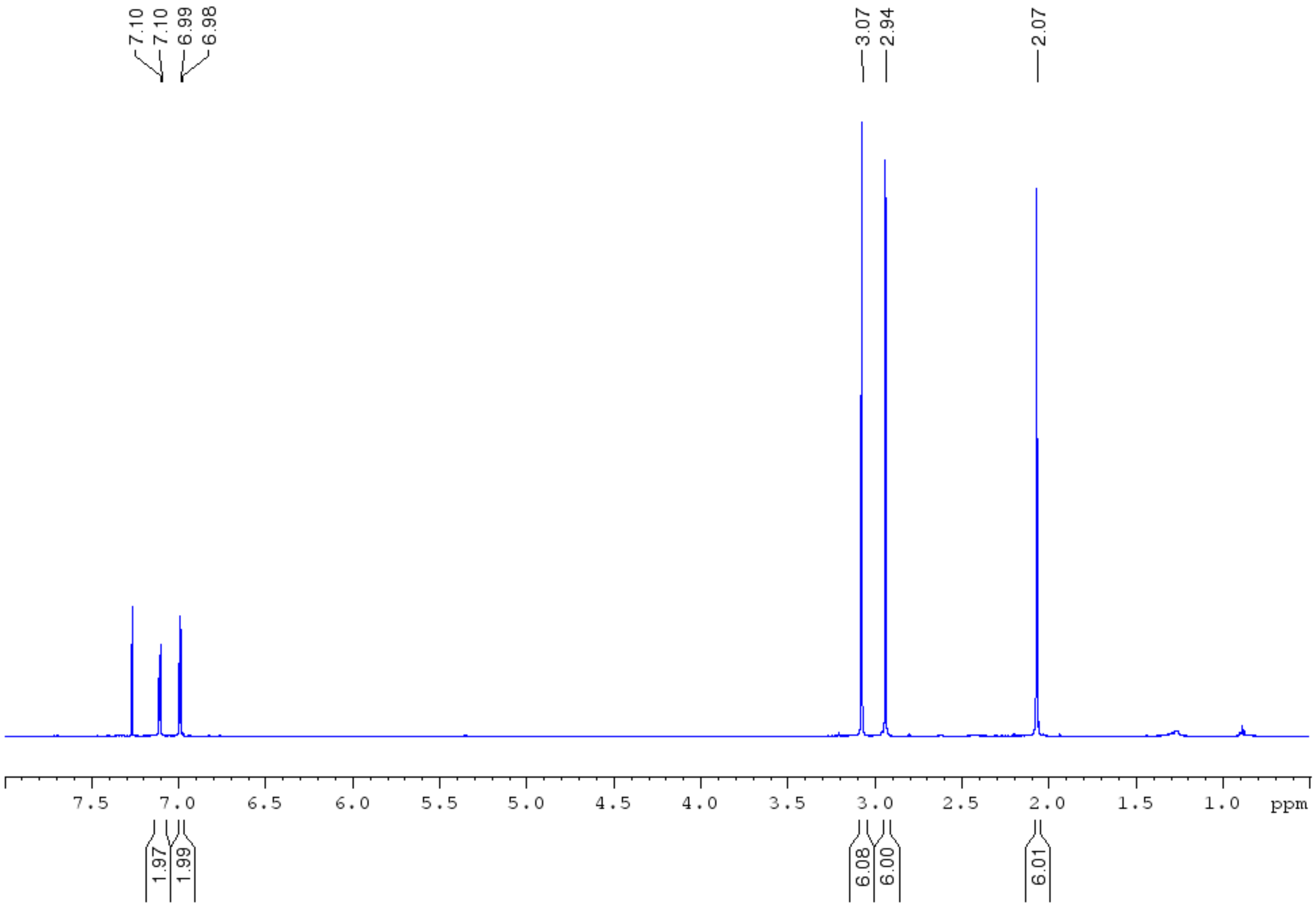

Figure S11. ^{1}H NMR spectrum of **$B_2(2\text{-}C_4H_2S\text{-}5\text{-}(CCMe))_2(NMe_2)_2$, 3** in $CDCl_3$.

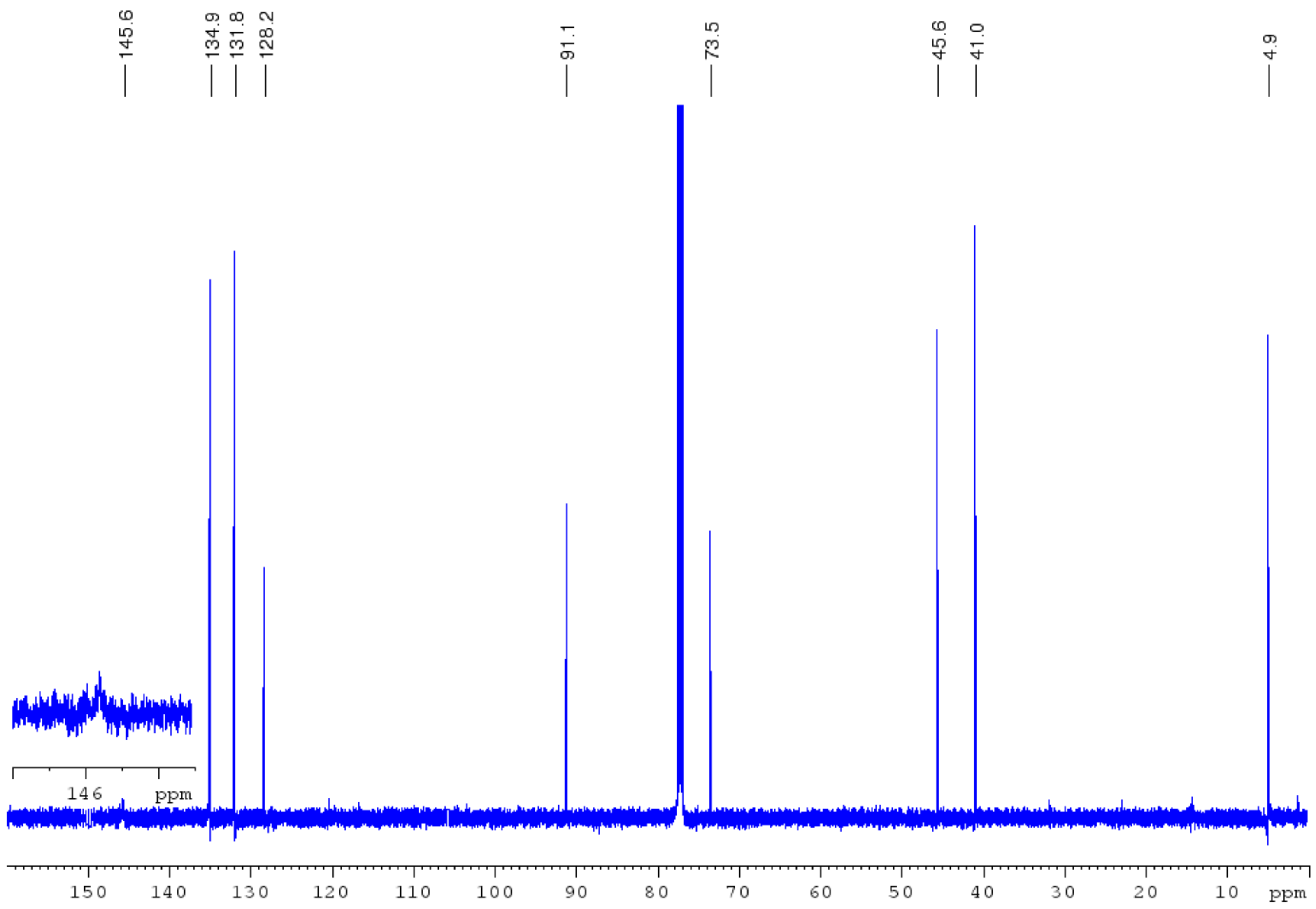

Figure S12. $^{13}C\{^{1}H\}$ NMR spectrum of **B_2(2-C_4H_2S-5-(CCMe))$_2$(NMe$_2$)$_2$**, **3** in $CDCl_3$.

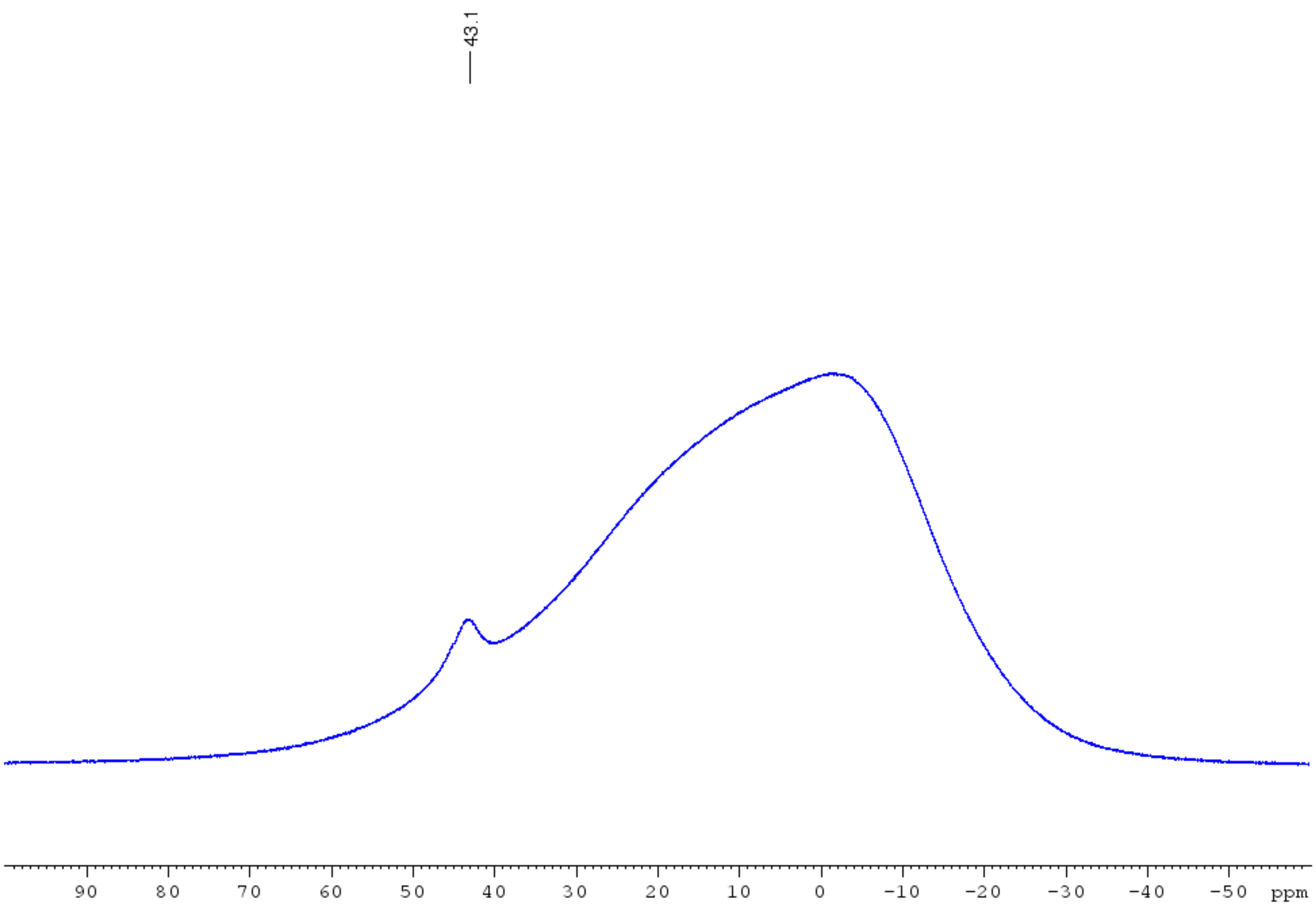

Figure S13. ^{11}B NMR spectrum of **B_2(2-C_4H_2S-5-(CCMe))$_2$(NMe_2)$_2$, 3** in $CDCl_3$.

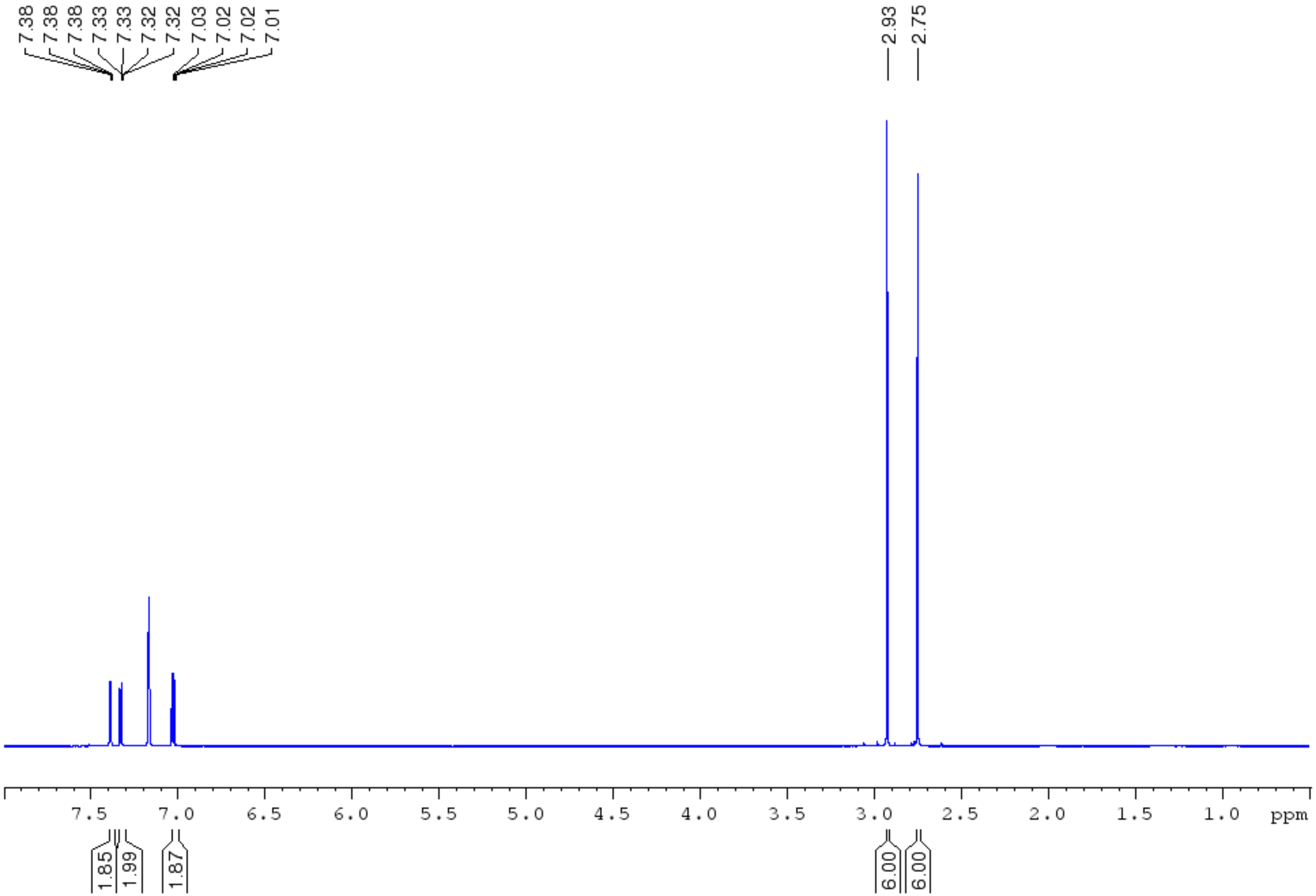

Figure S14. ^{1}H NMR spectrum of $\mathbf{B_2(C_4H_3S)_2(NMe_2)_2}$, **4** in CD_2Cl_2.

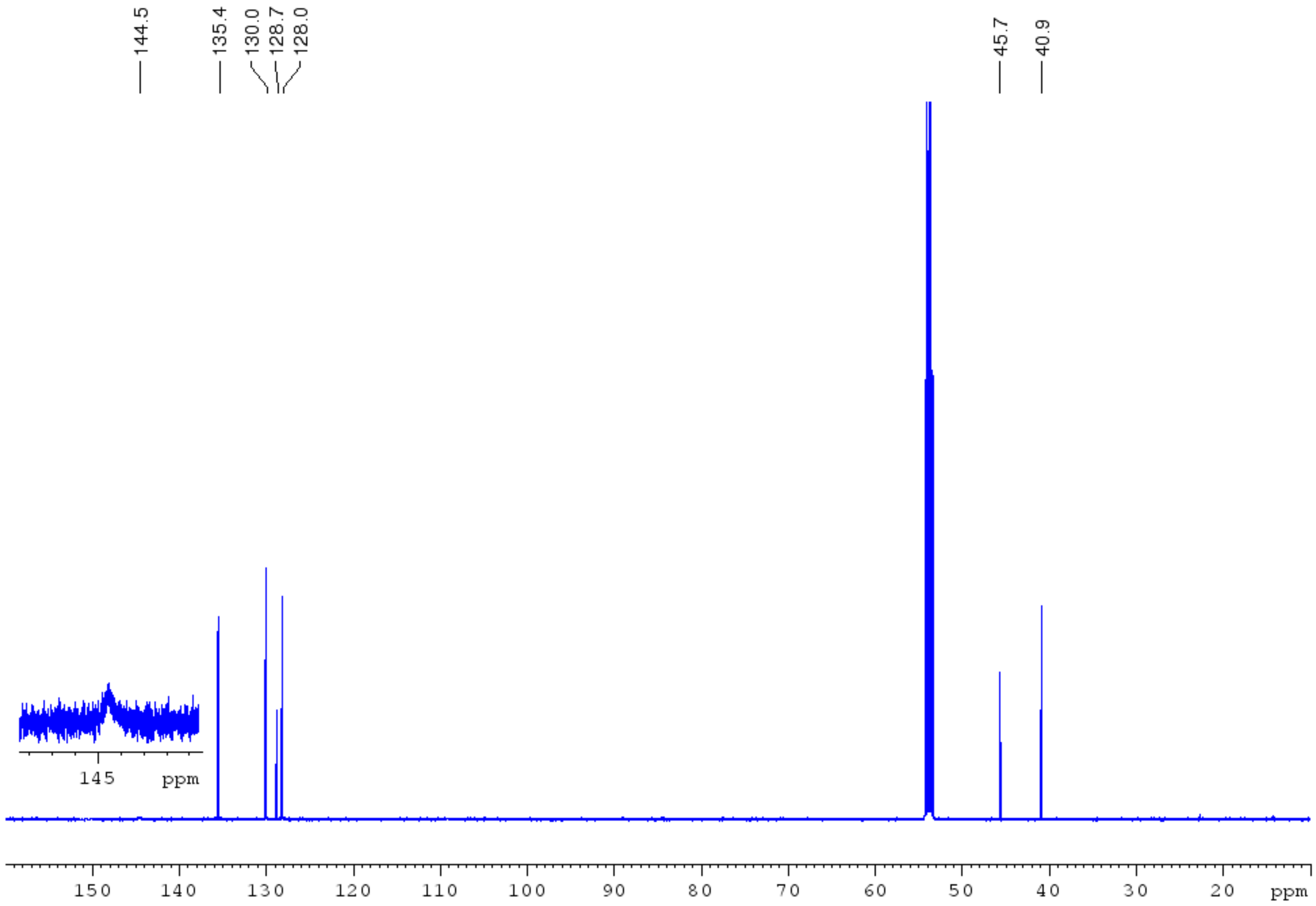

Figure S15. $^{13}C\{^1H\}$ NMR spectrum of $\mathbf{B_2(C_4H_3S)_2(NMe_2)_2}$, **4** in CD_2Cl_2.

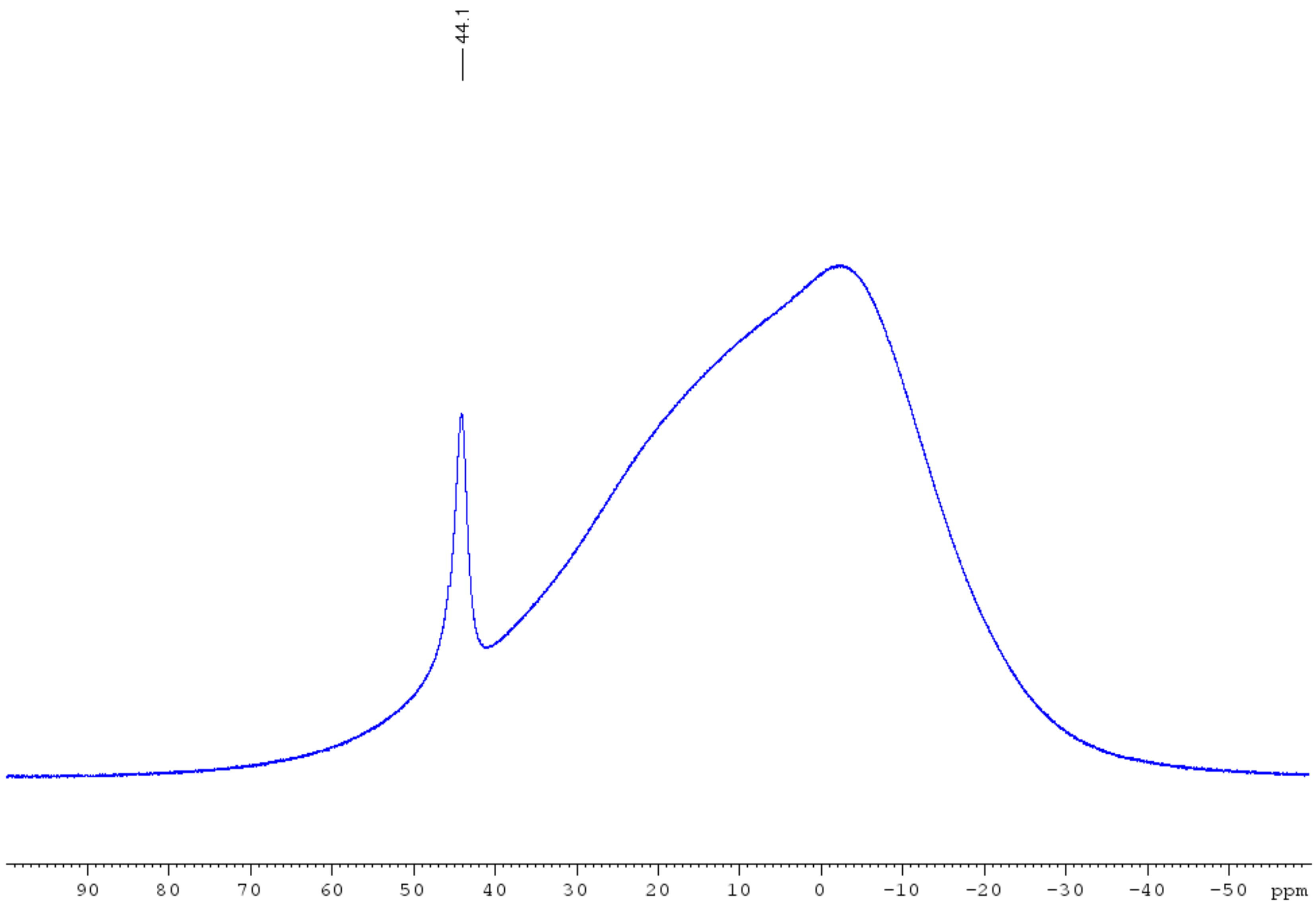

Figure S16. ^{11}B NMR spectrum of **$B_2(C_4H_3S)_2(NMe_2)_2$**, **4** in CD_2Cl_2.

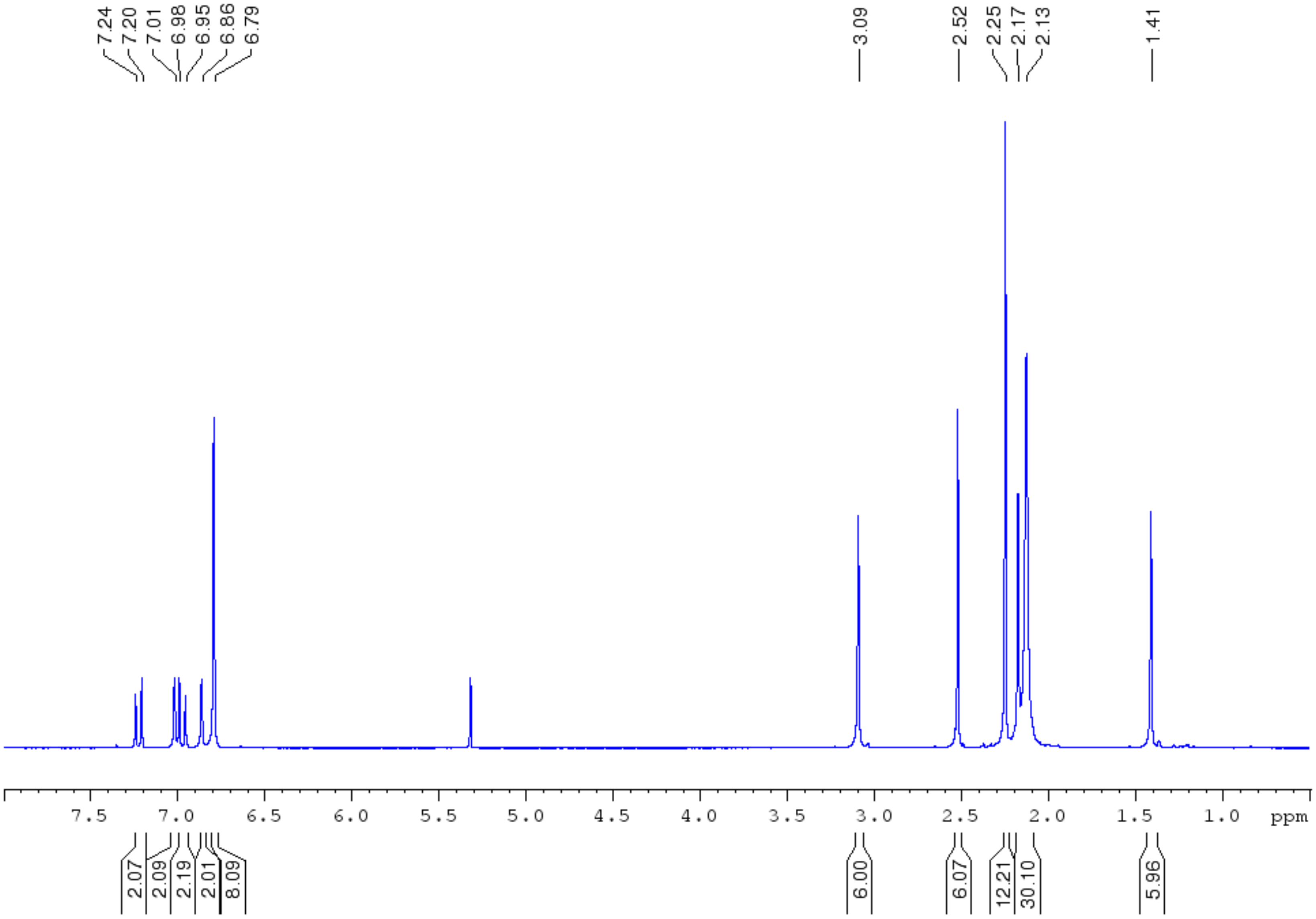

Figure S17. ^{1}H NMR spectrum of **$B_2(C_6H_2$-2,6-Me_2-4-$(CHCHBMes_2))_2(NMe_2)_2$**, **5a** in CD_2Cl_2.

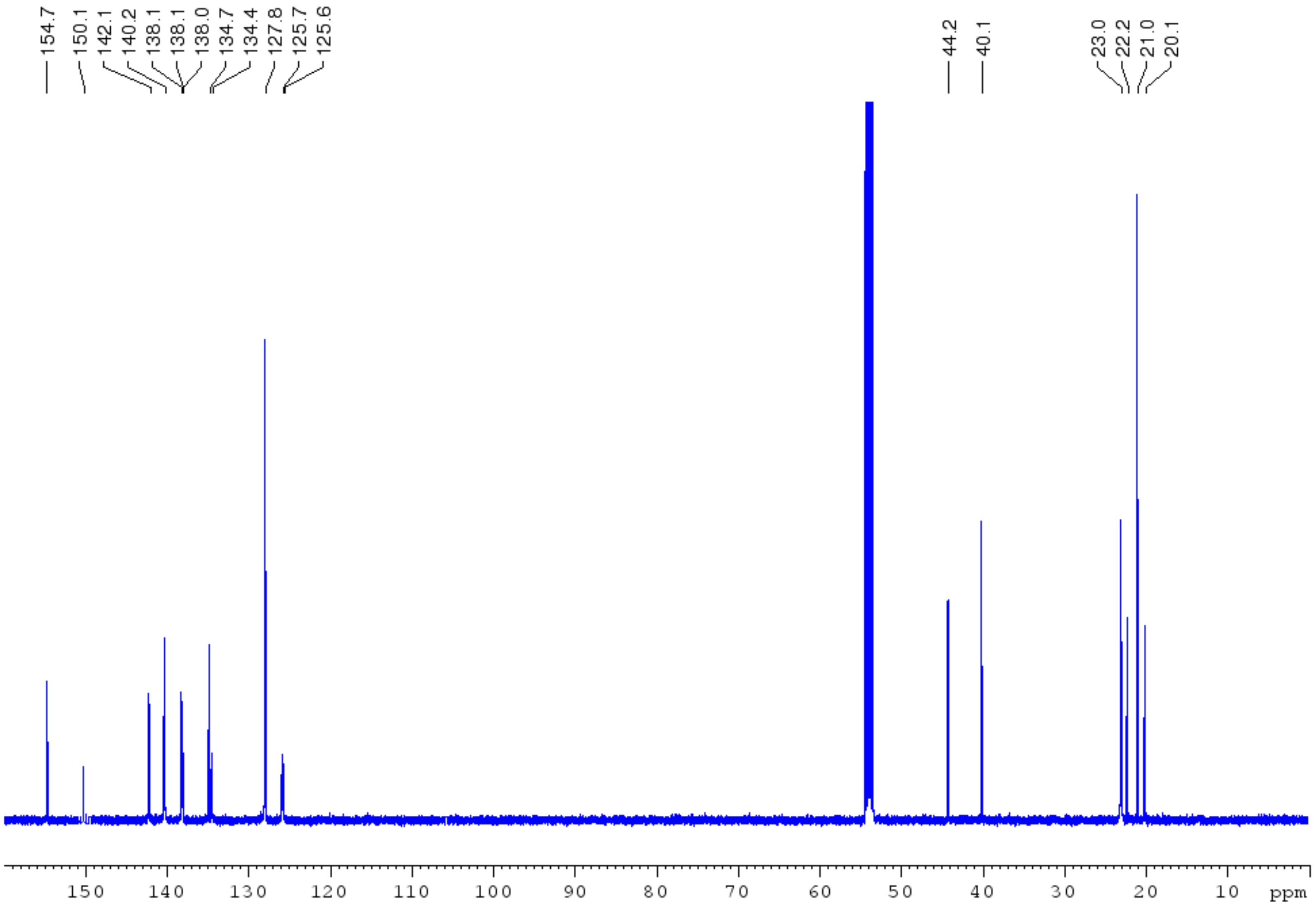

Figure S18. $^{13}C\{^{1}H\}$ NMR spectrum of **$B_2(C_6H_2$-2,6-Me_2-4-$(CHCHBMes_2))_2(NMe_2)_2$, 5a** in CD_2Cl_2.

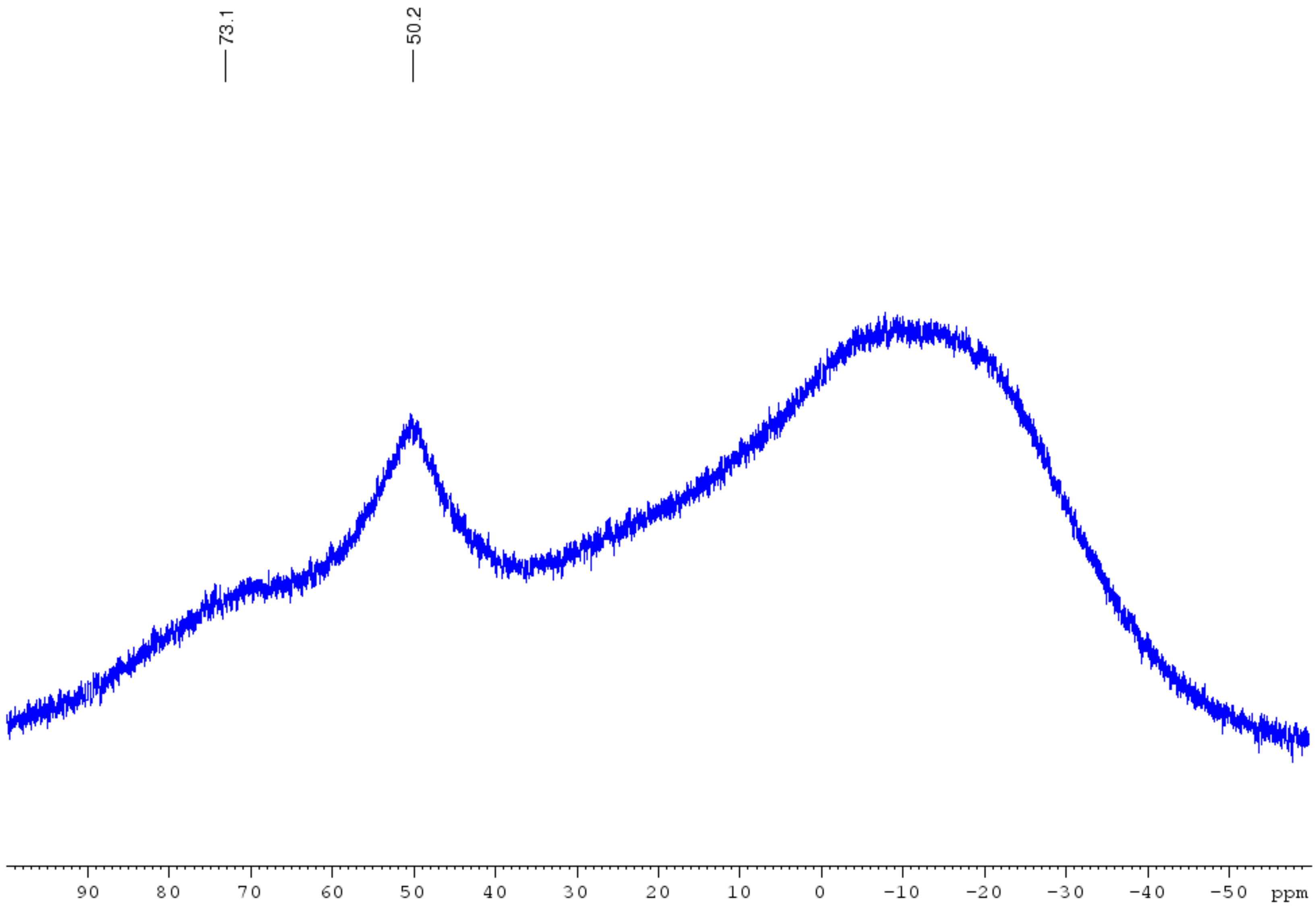

Figure S19. ^{11}B NMR spectrum of **$B_2(C_6H_2$-2,6-Me_2-4-$(CHCHBMes_2))_2(NMe_2)_2$, 5a** in $CDCl_3$.

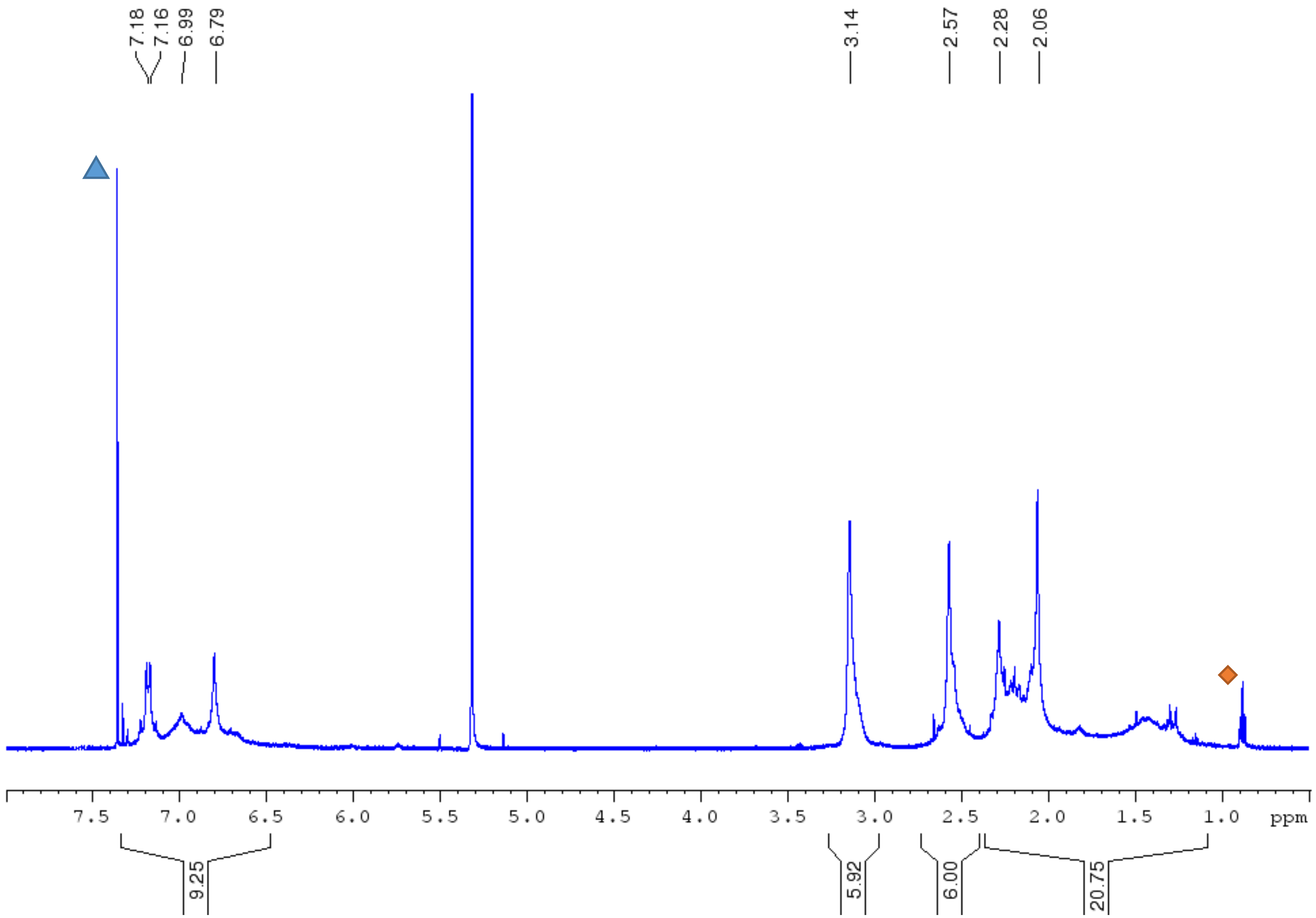

Figure S20. ^{1}H NMR spectrum of **Oligomerization product of 1**, **5b** in CD_2Cl_2. The resonances marked with ▲ correspond to residual benzene. The resonances marked with ◆ correspond to residual hexane.

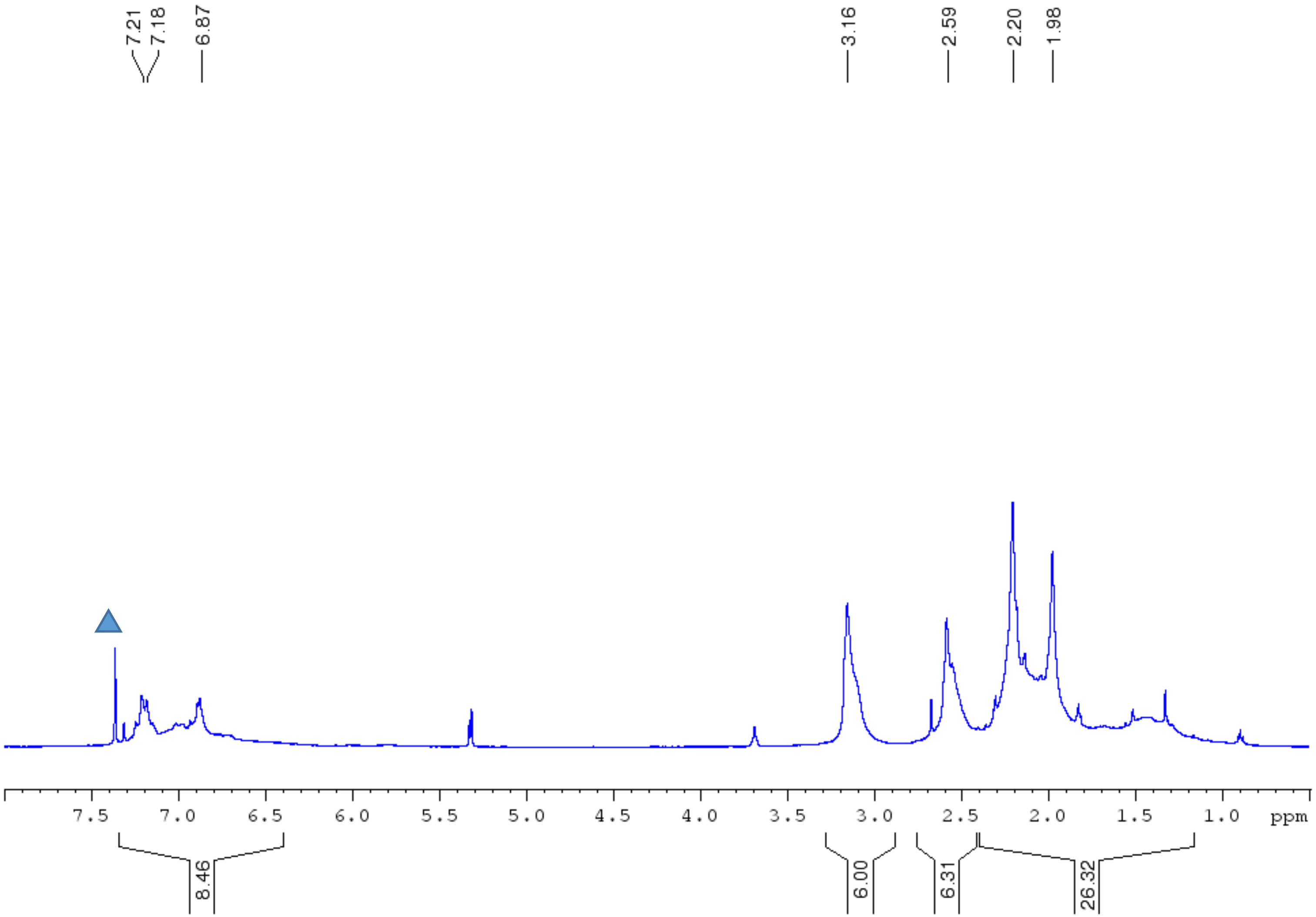

Figure S21. ^{1}H NMR spectrum of **Oligomerization product of 1**, **5c** in CD_2Cl_2. The resonances marked with △ correspond to residual benzene.

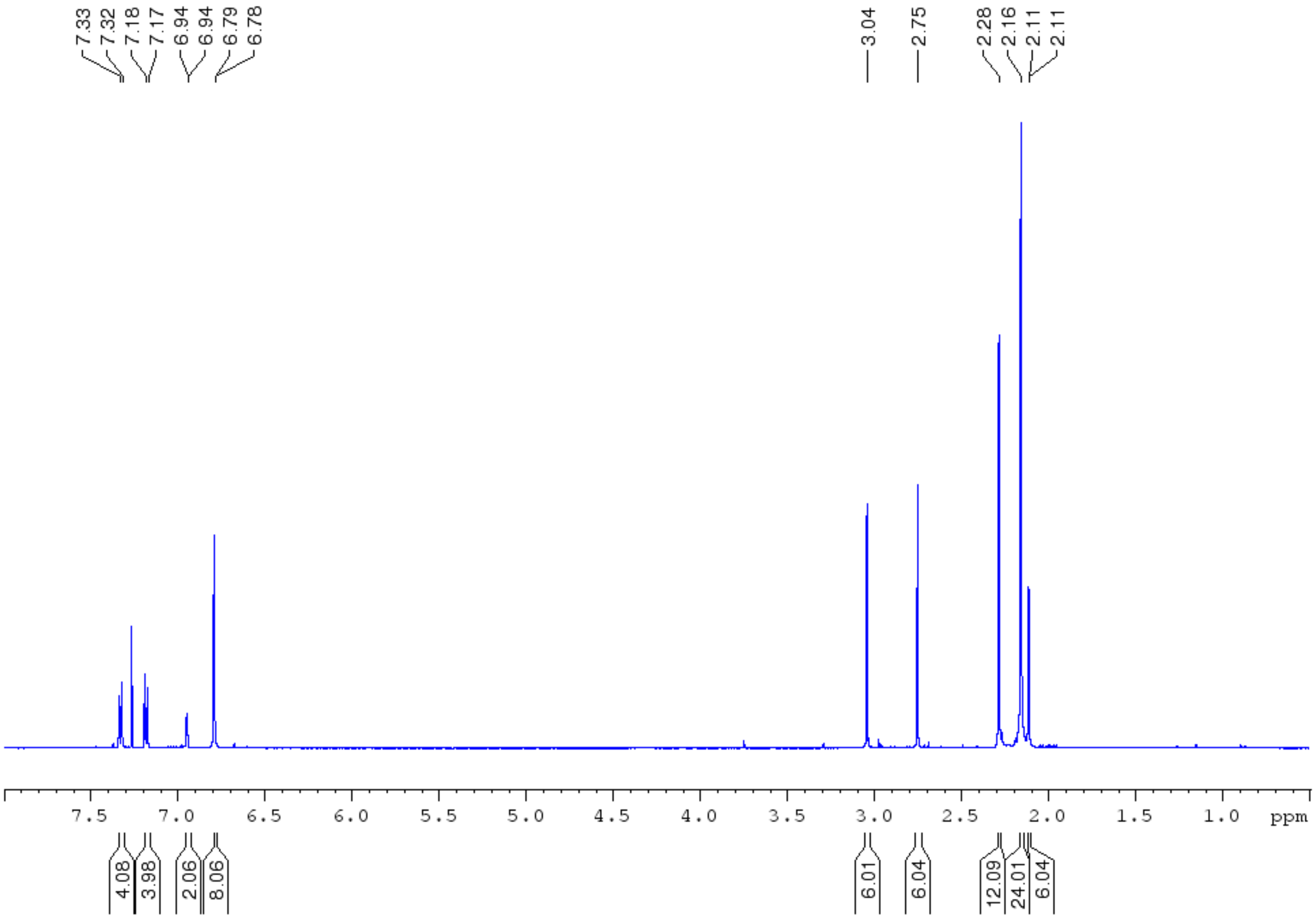

Figure S22. ^{1}H NMR spectrum of **$B_2(C_6H_4$-4-$(CHCMeBMes_2))_2(NMe_2)_2$, 6a** in $CDCl_3$.

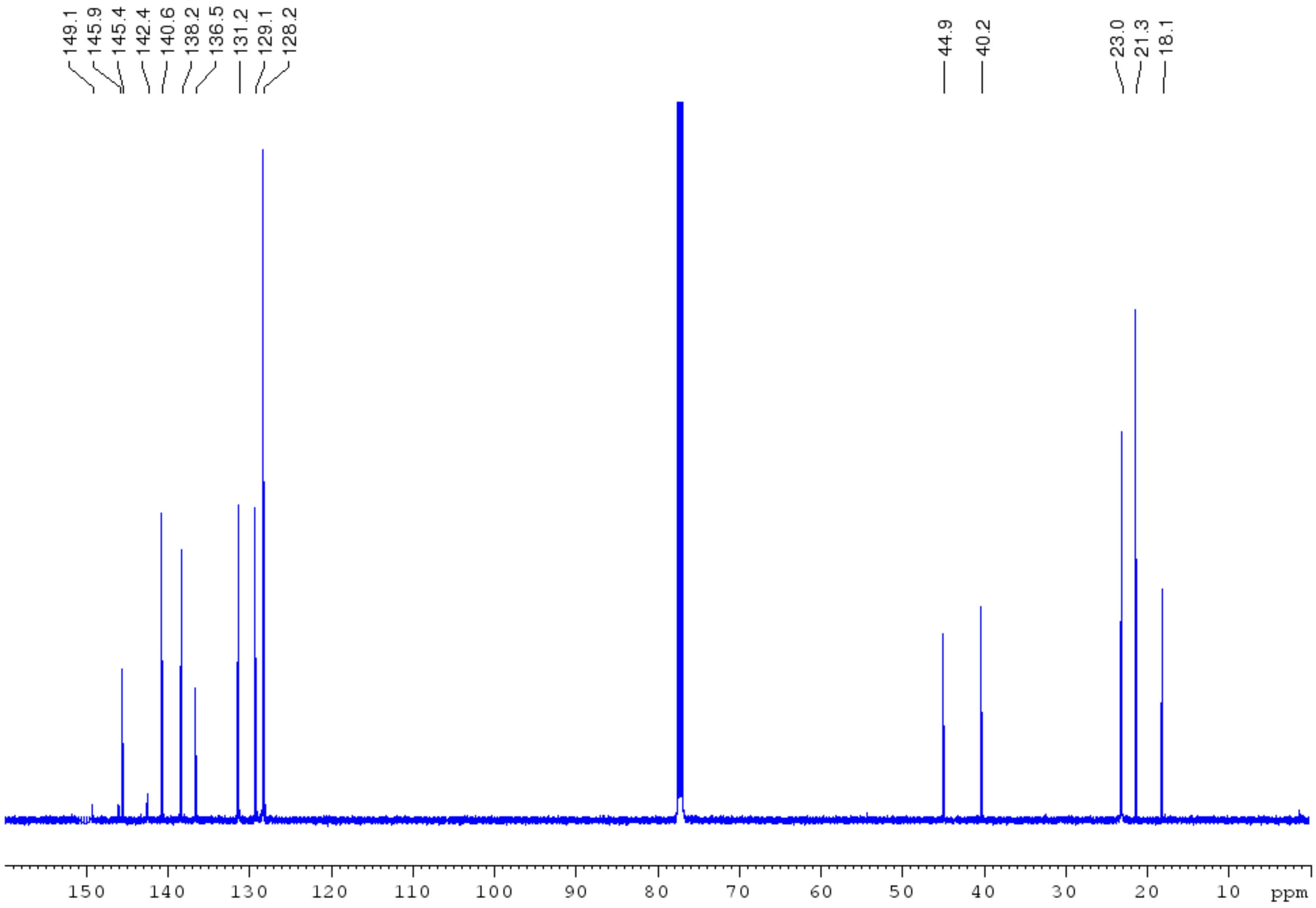

Figure S23. $^{13}C\{^1H\}$ NMR spectrum of **$B_2(C_6H_4$-4-$(CHCMeBMes_2))_2(NMe_2)_2$**, **6a** in $CDCl_3$.

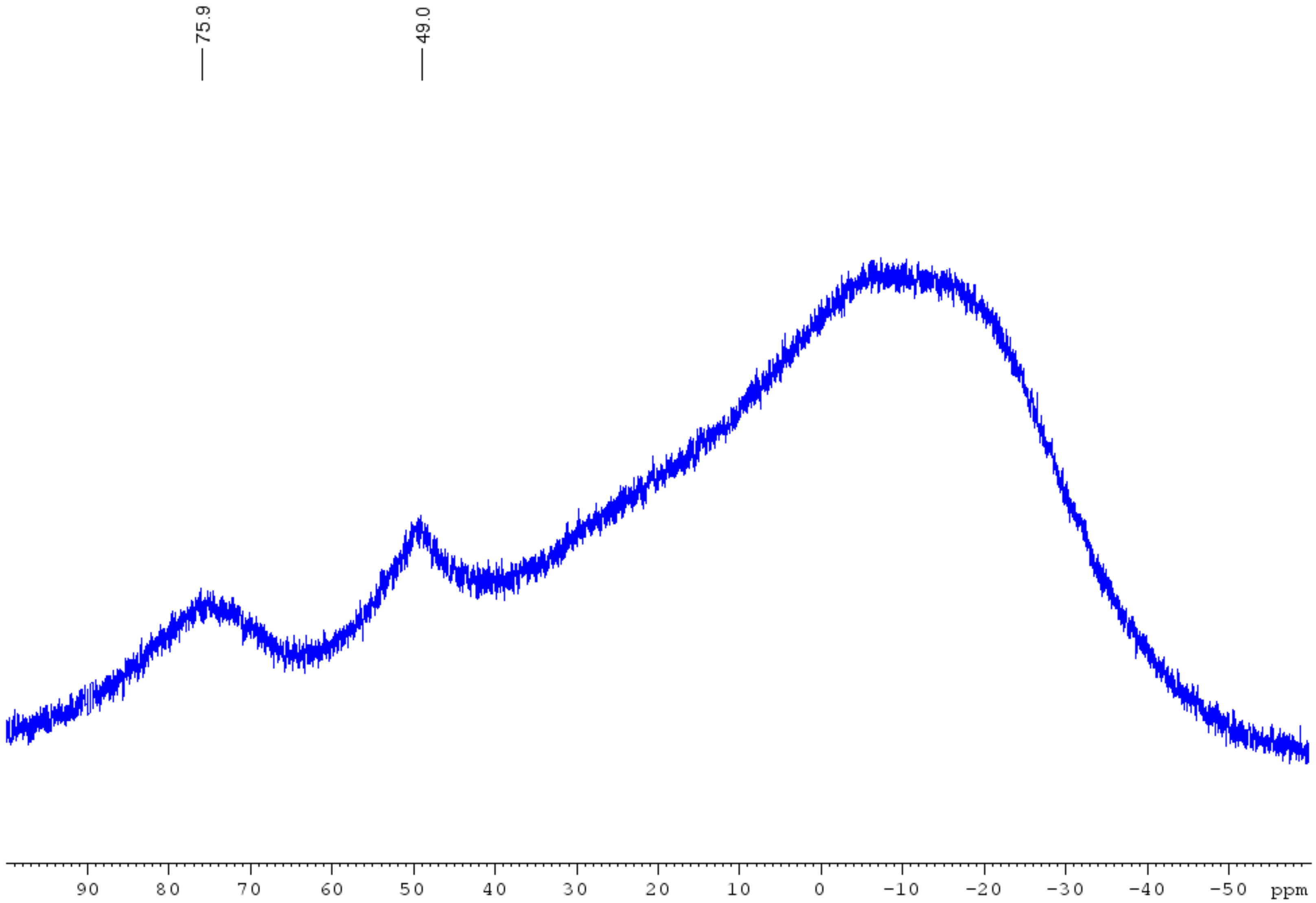

Figure S24. ^{11}B NMR spectrum of **$B_2(C_6H_4$-4-$(CHCMeBMes_2))_2(NMe_2)_2$**, **6a** in C_6D_6.

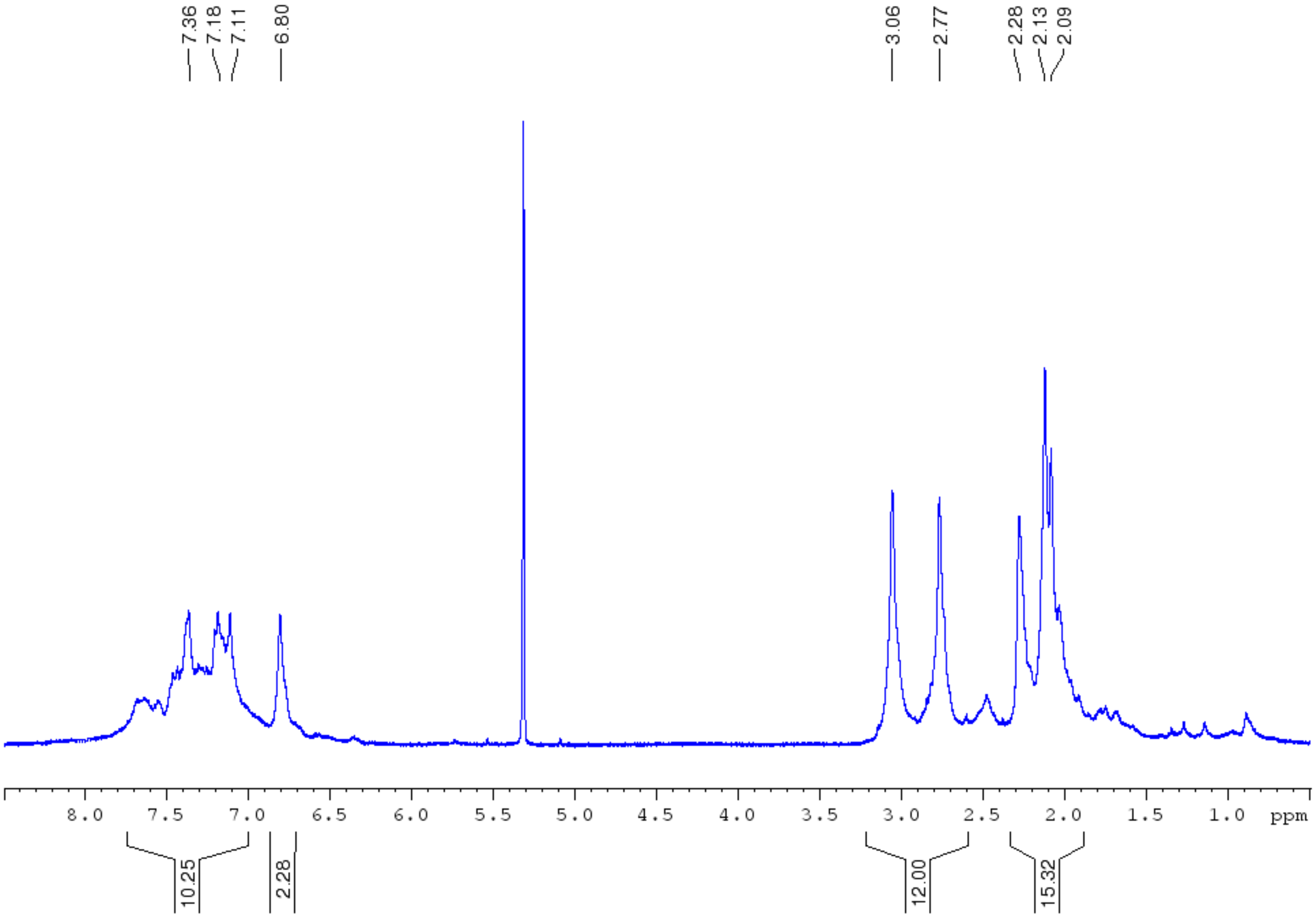

Figure S25. ^{1}H NMR spectrum of **Oligomerization product of 2**, **6b** in CD_2Cl_2.

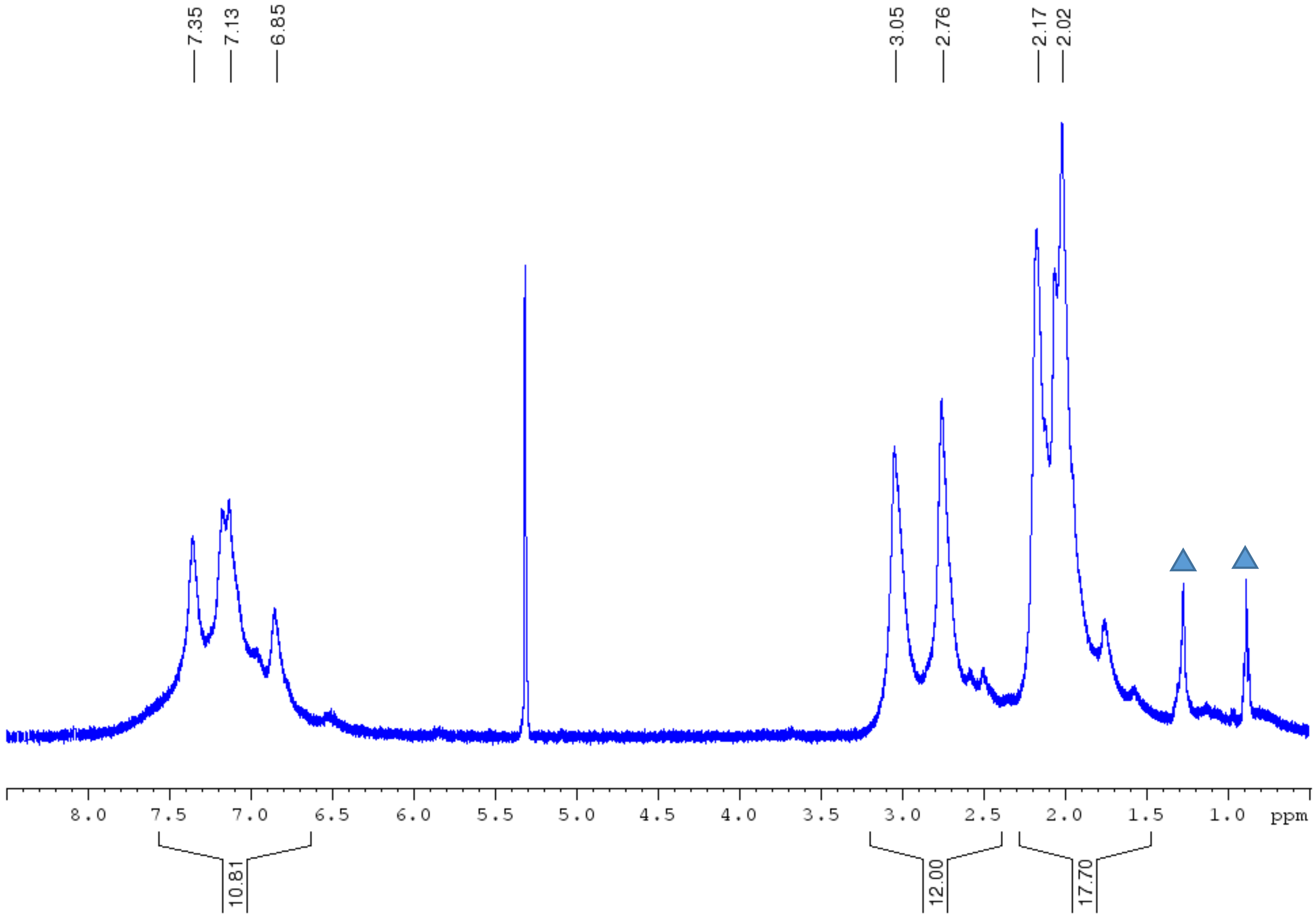

Figure S26. ^{1}H NMR spectrum of **Oligomerization product of 2**, **6c** in CD_2Cl_2. The resonances marked with △ correspond to residual hexane.

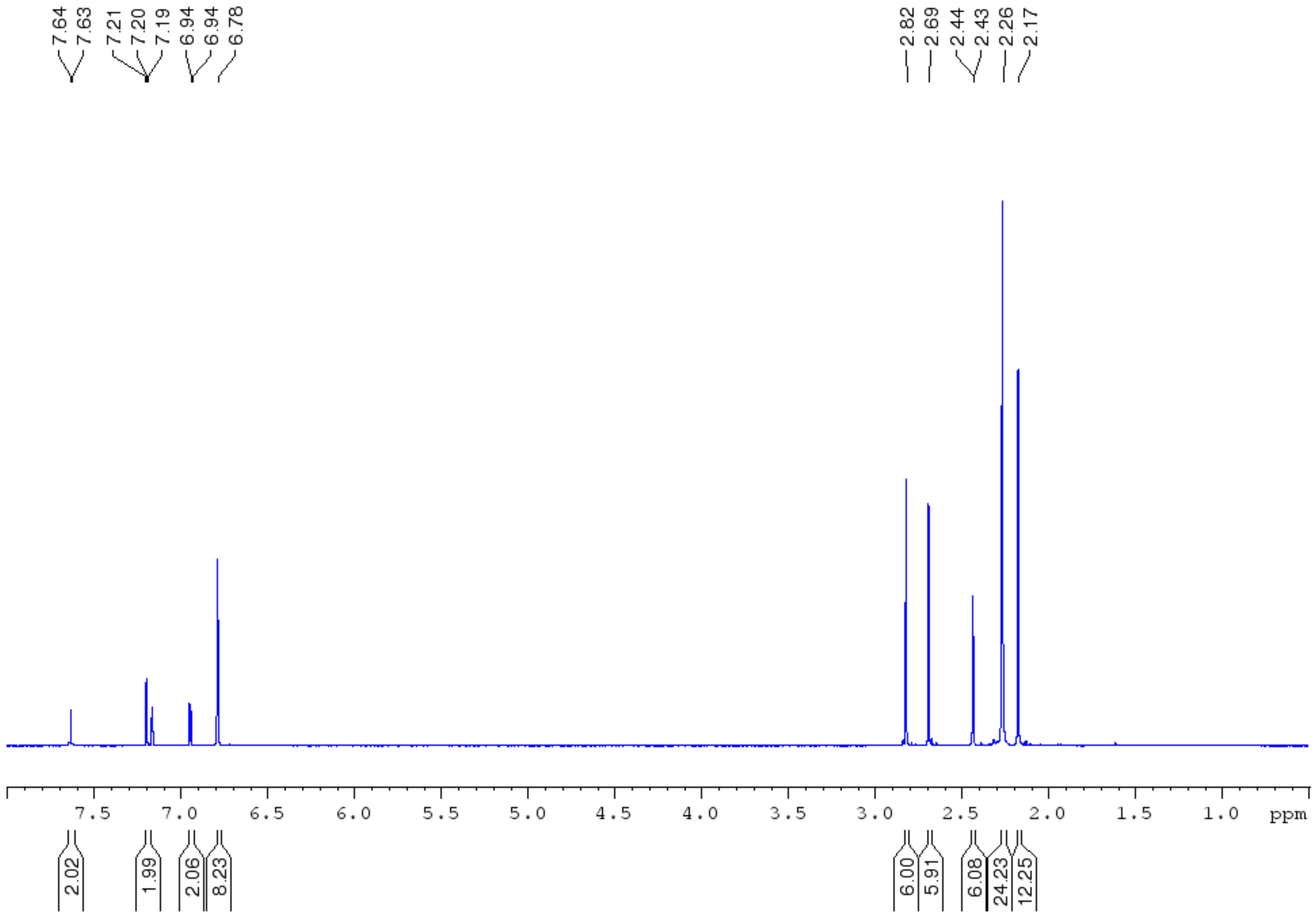

Figure S27. ^{1}H NMR spectrum of **B_2(2-C_4H_2S-5-(CHCMeBMes$_2$))$_2$(NMe$_2$)$_2$**, **7a** in C_6D_6.

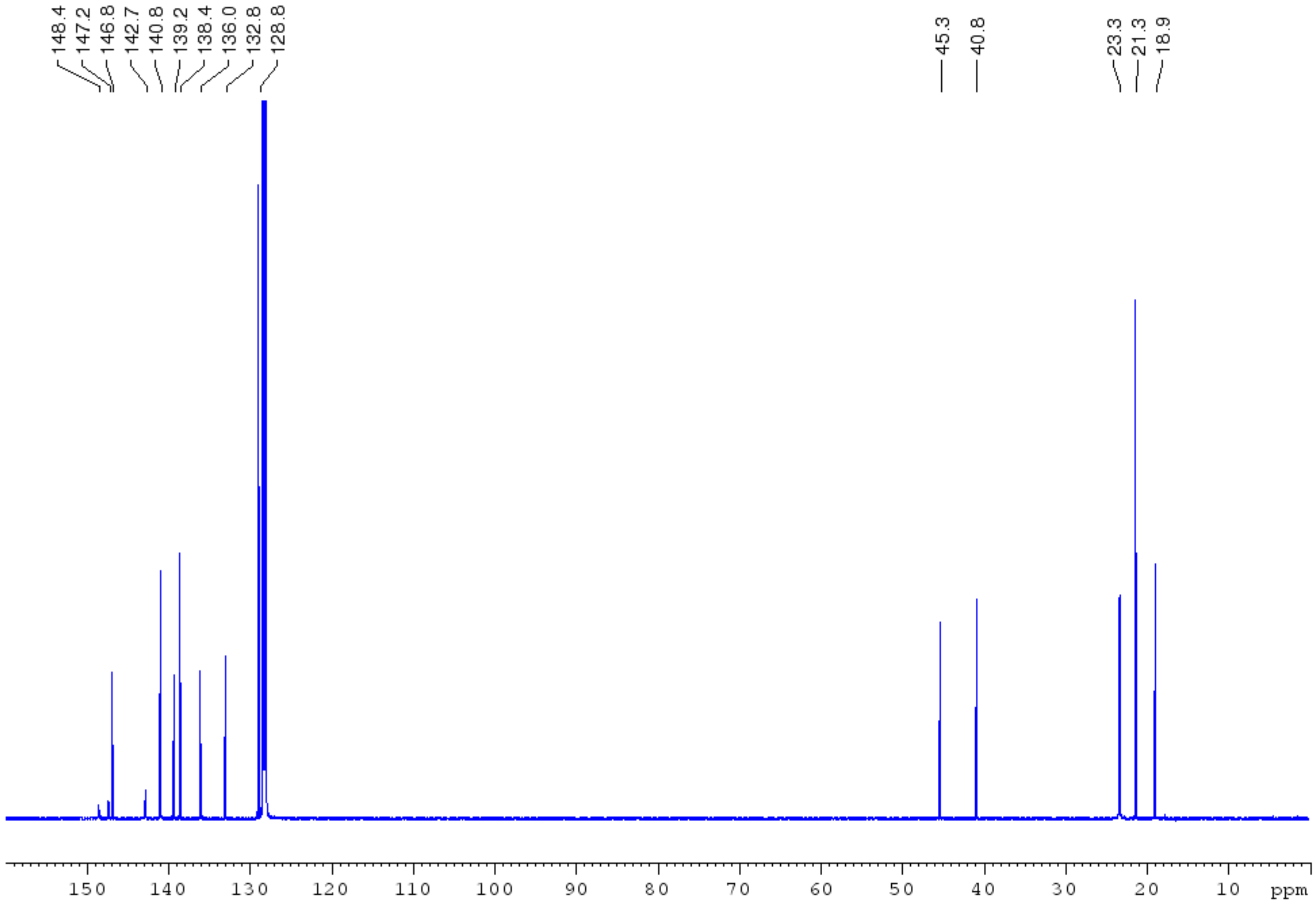

Figure S28. $^{13}C\{^{1}H\}$ NMR spectrum of **$B_2(2\text{-}C_4H_2S\text{-}5\text{-}(CHCMeBMes_2))_2(NMe_2)_2$, 7a** in C_6D_6.

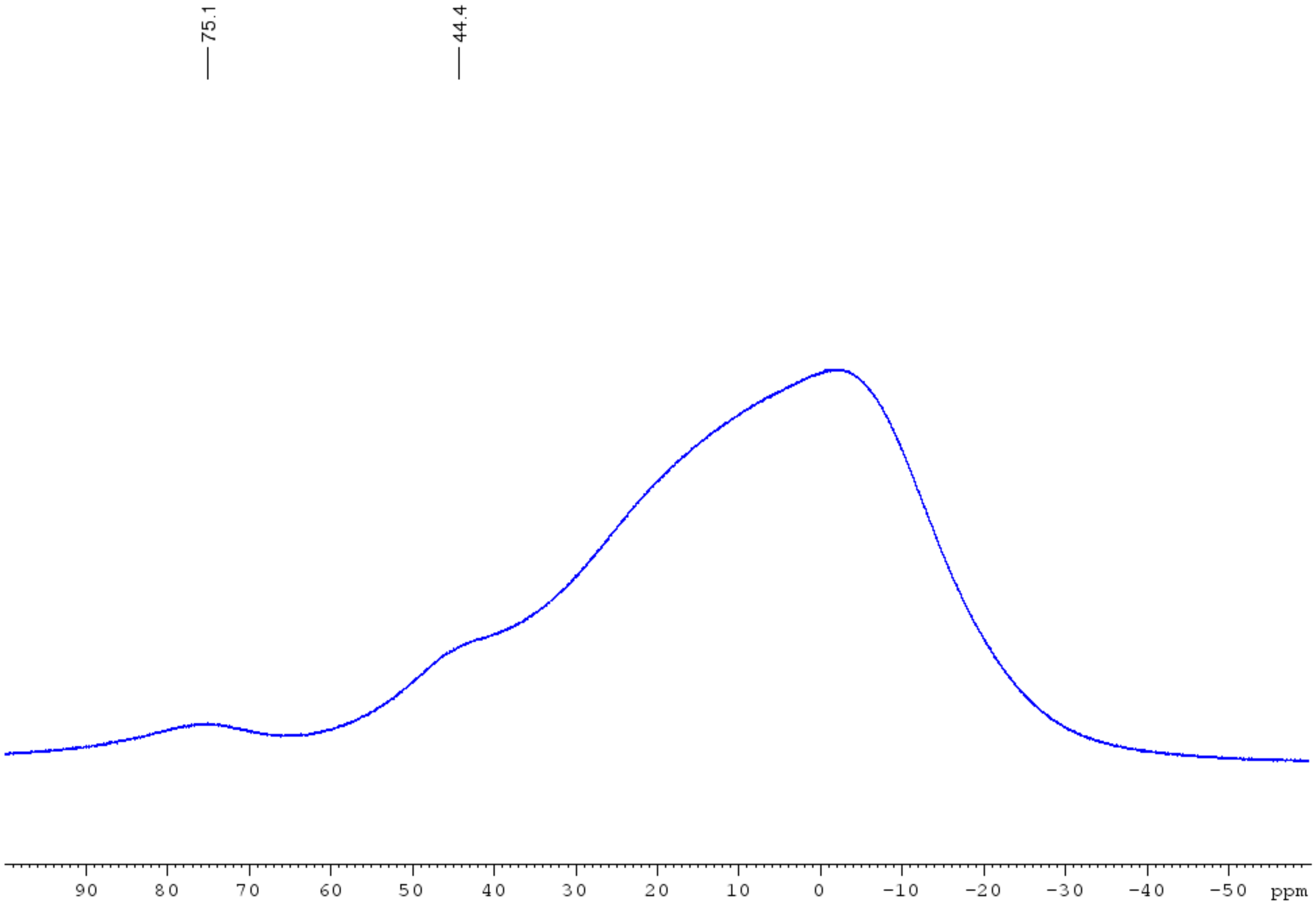

Figure S29. ^{11}B NMR spectrum of **B_2(2-C_4H_2S-5-(CHCMeBMes$_2$))$_2$(NMe$_2$)$_2$**, **7a** in C_6D_6.

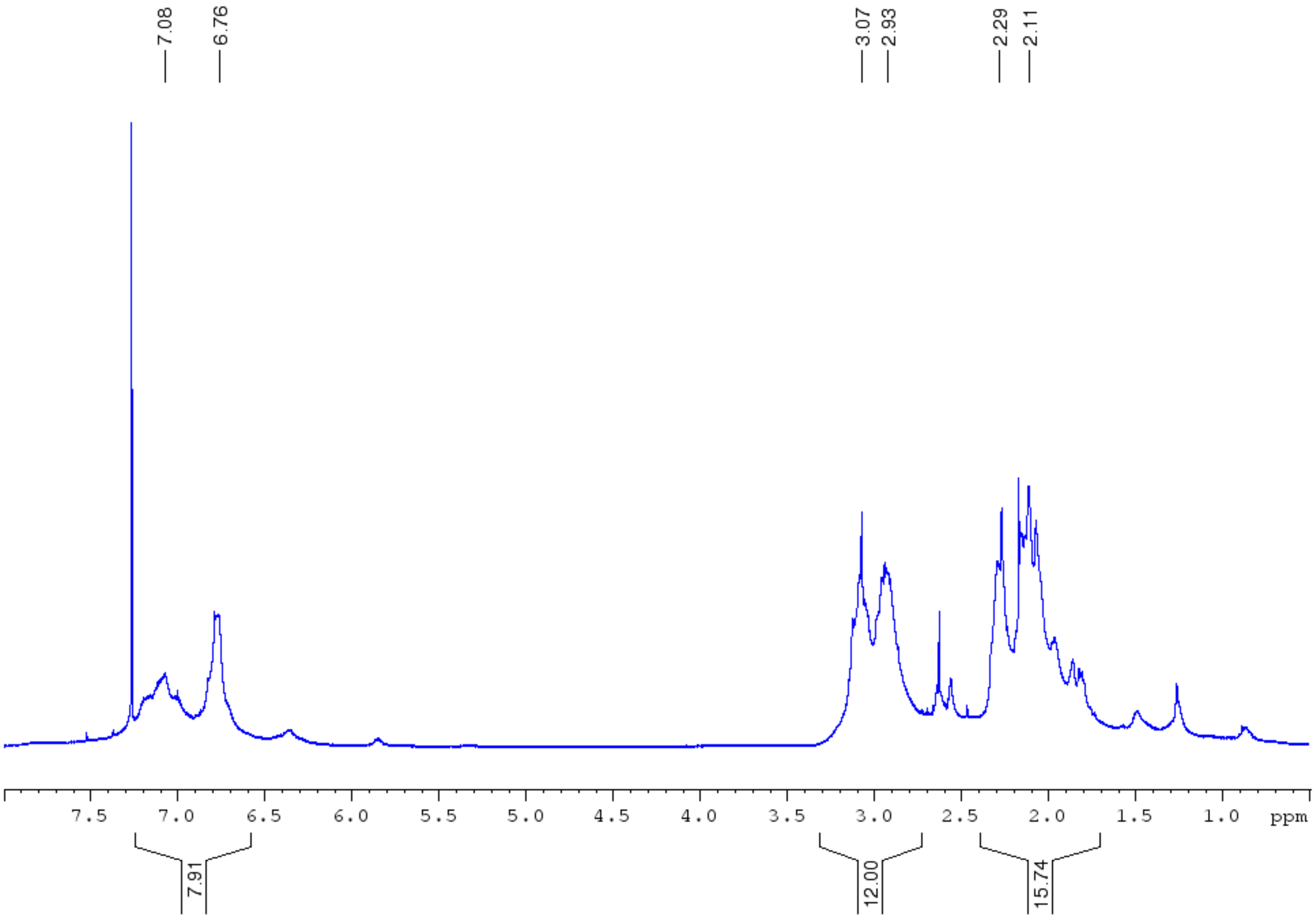

Figure S30. ^{1}H NMR spectrum of **Oligomerization product of 3**, **7b** in $CDCl_3$.

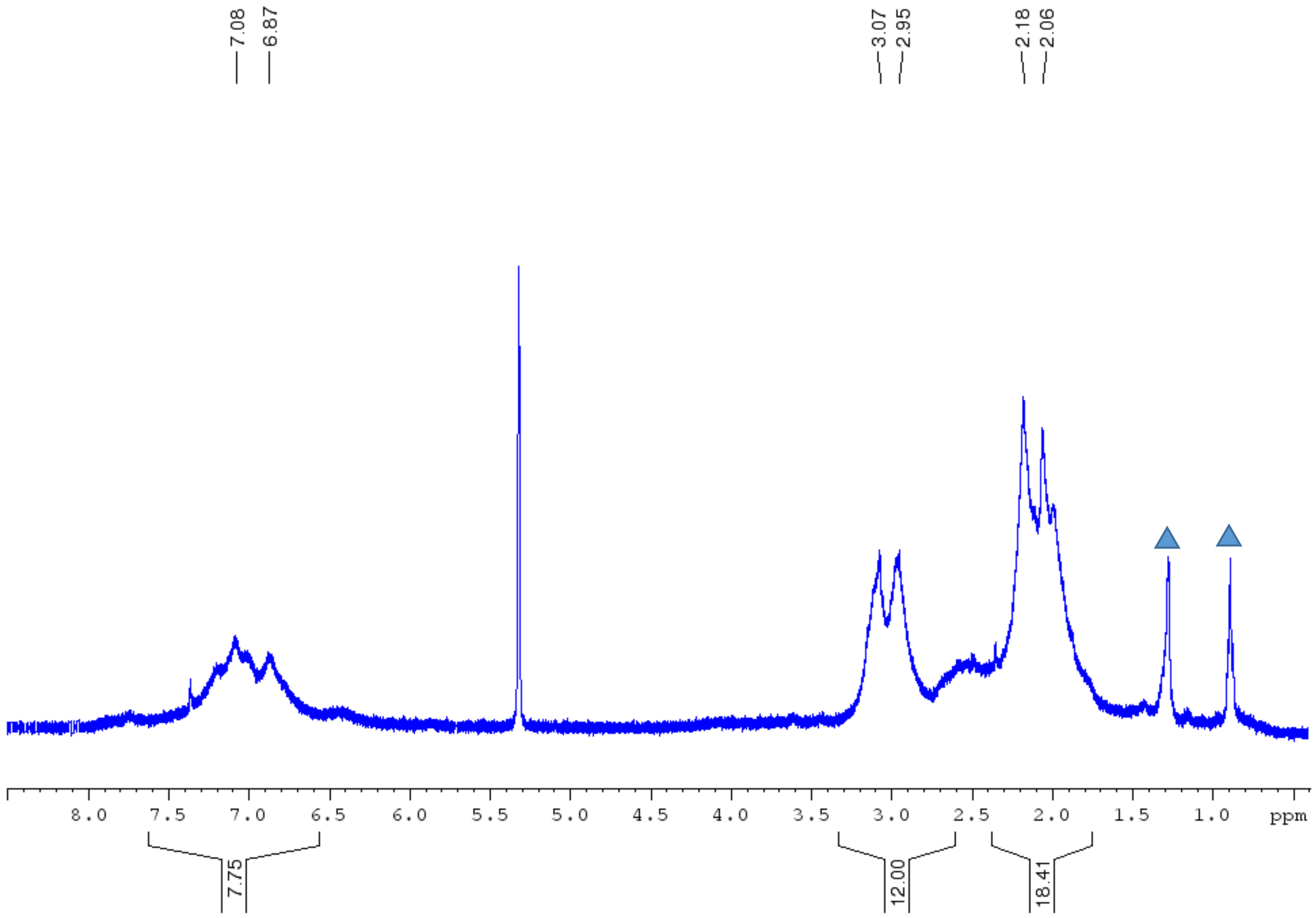

Figure S31. ^{1}H NMR spectrum of **Oligomerization product of 3**, **7c** in CD_2Cl_2. The resonances marked with ▲ correspond to residual hexane.

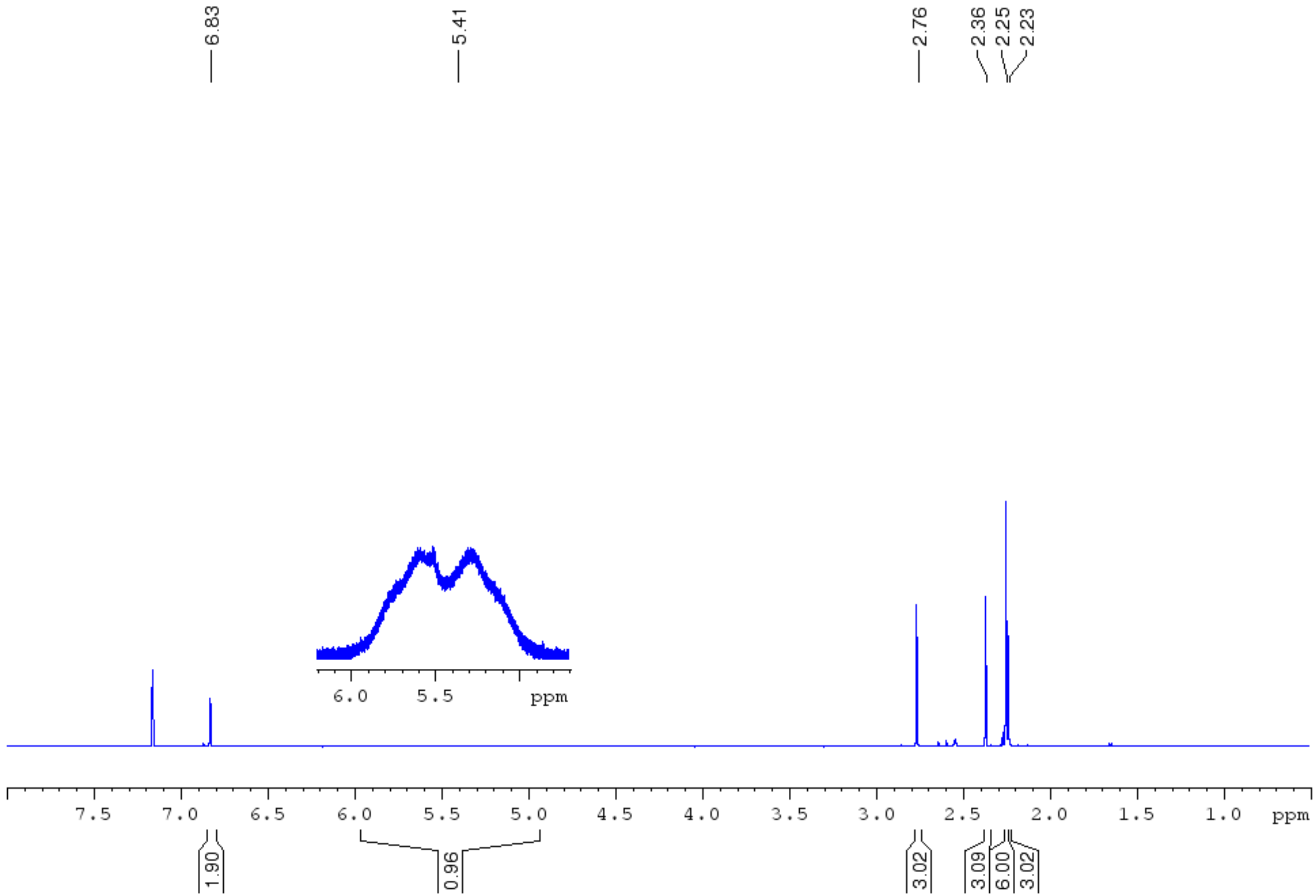

Figure S32. ^{1}H NMR spectrum of **HBMes(NMe$_2$)**, **8** in C_6D_6.

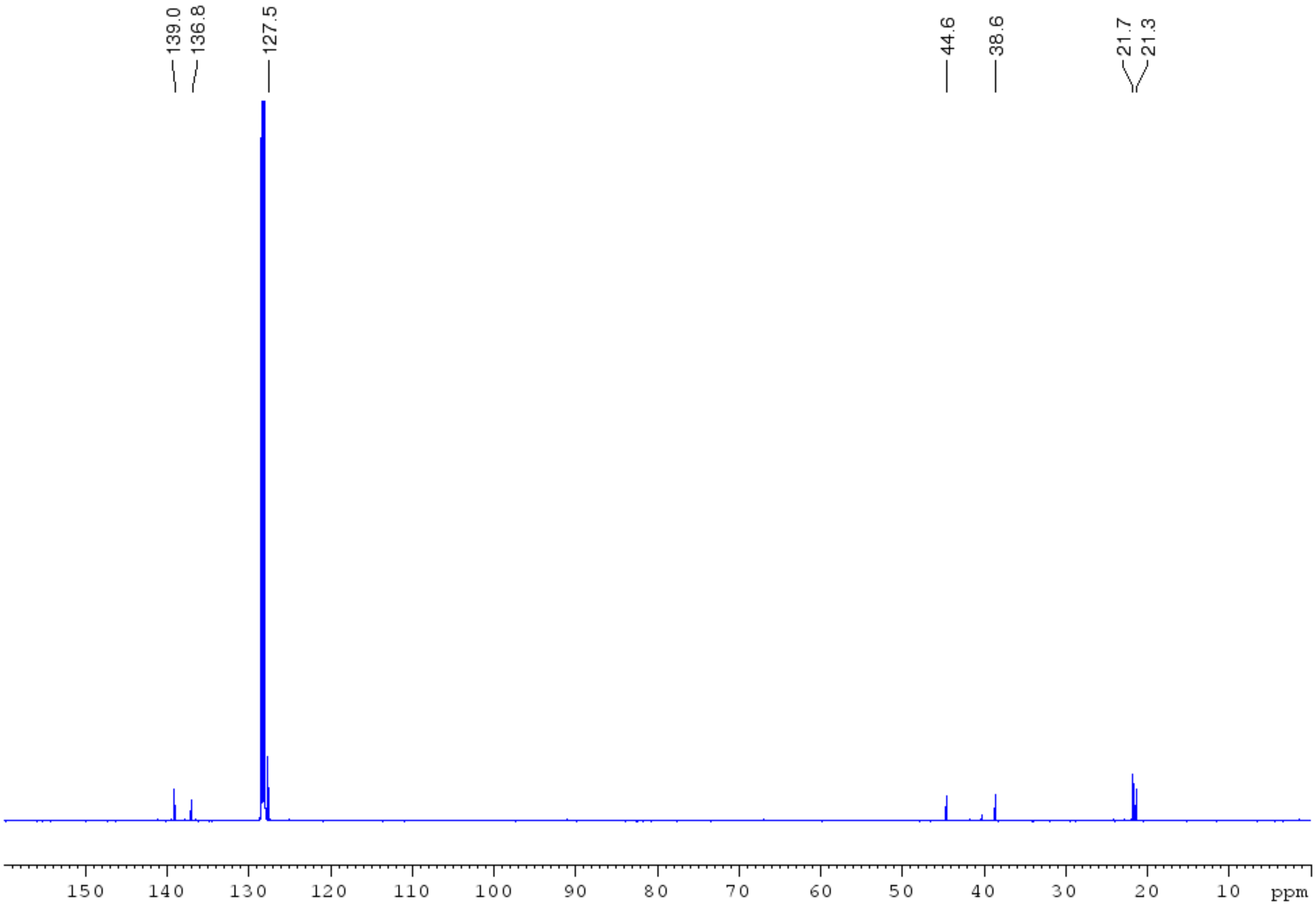

Figure S33. $^{13}C\{^{1}H\}$ NMR spectrum of **HBMes(NMe$_2$)**, **8** in C_6D_6.

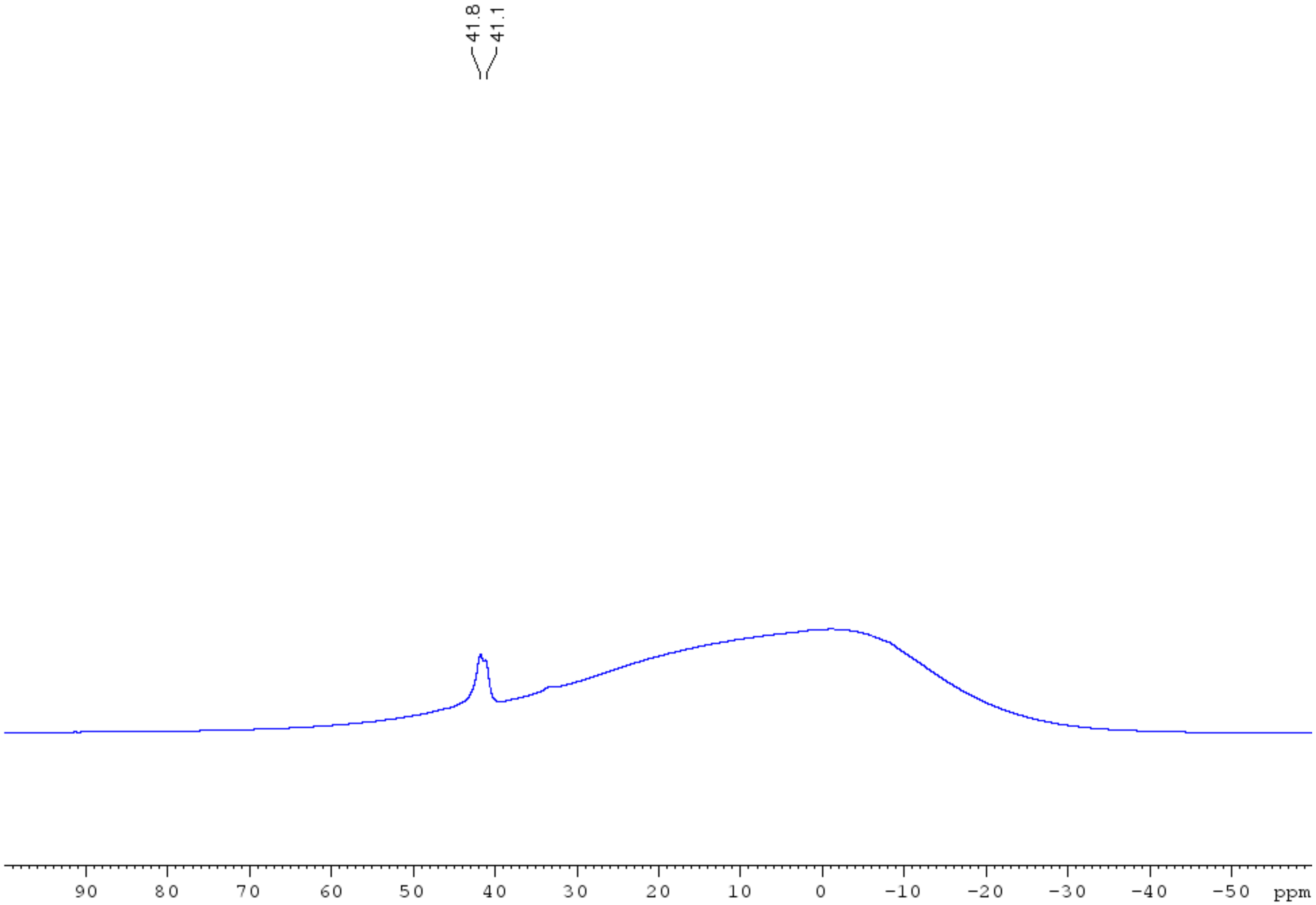

Figure S34. ^{11}B NMR spectrum of **HBMes(NMe$_2$)**, **8** in C_6D_6.

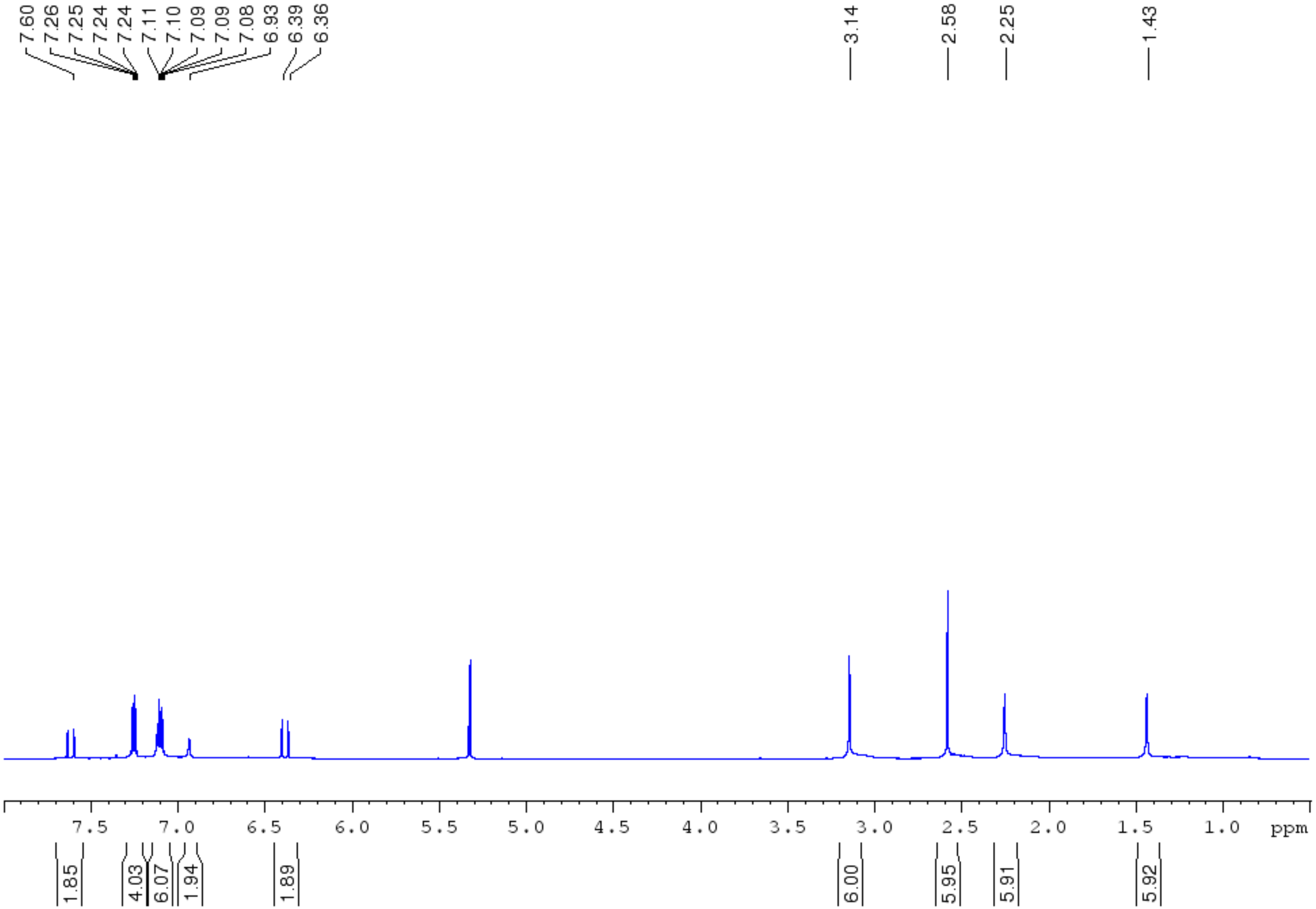

Figure S35. ^{1}H NMR spectrum of **$B_2(C_6H_2$-2,6-Me_2-4-(CHCHBCat))$_2(NMe_2)_2$, 10** in CD_2Cl_2.

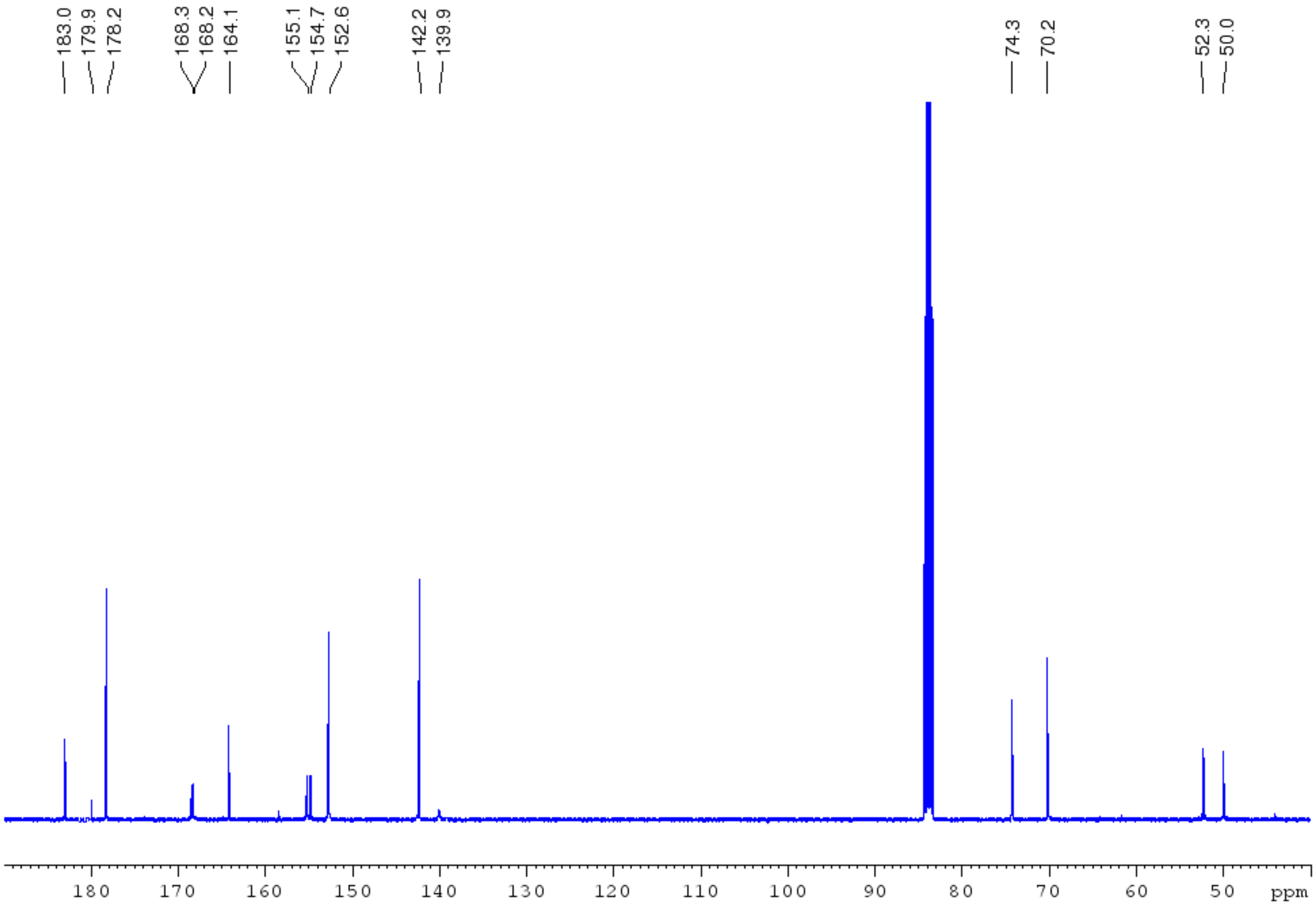

Figure S36. $^{13}C\{^{1}H\}$ NMR spectrum of **$B_2(C_6H_2$-2,6-Me_2-4-(CHCHBCat))$_2(NMe_2)_2$**, **10** in CD_2Cl_2.

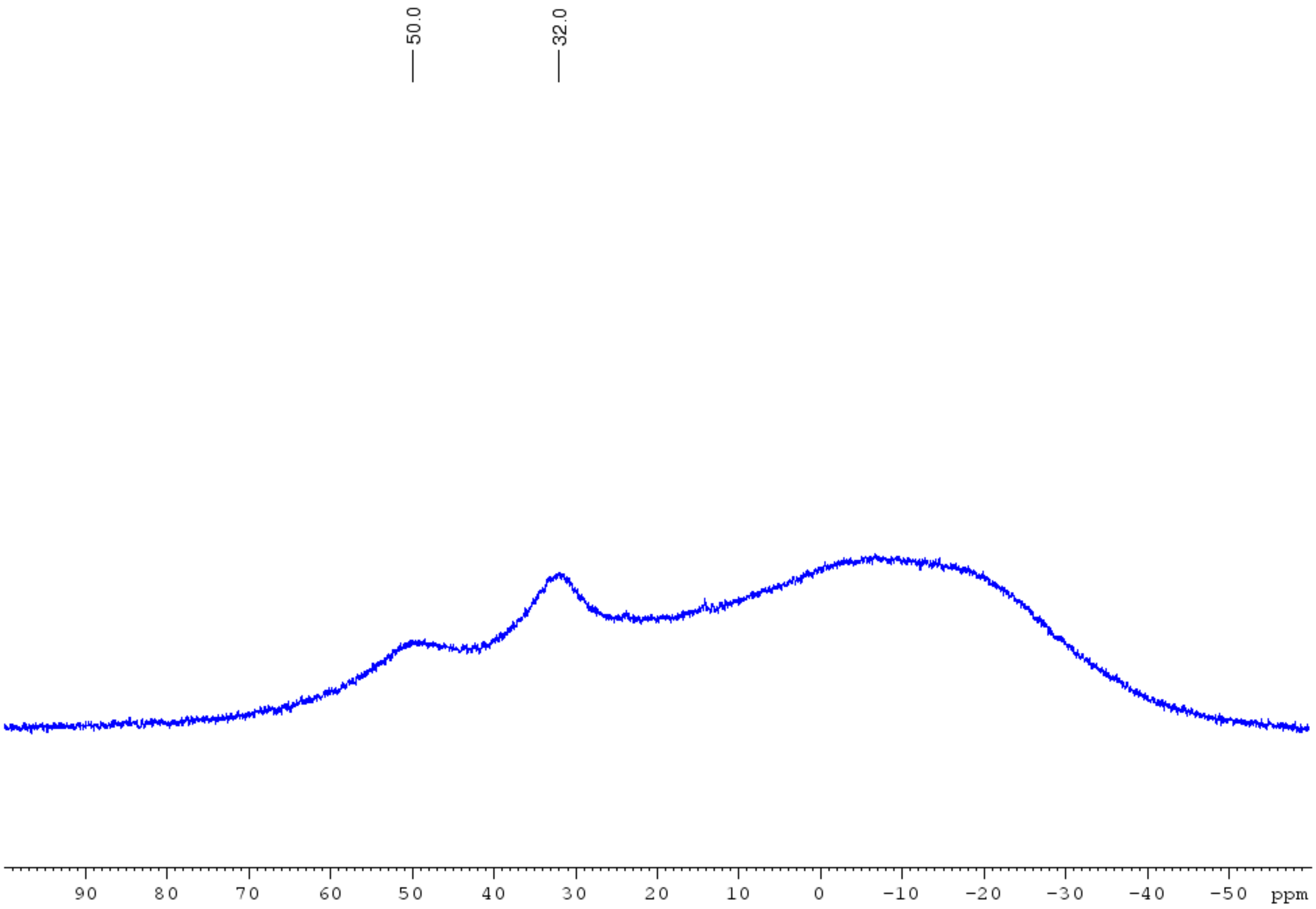

Figure S37. ^{11}B NMR spectrum of **$B_2(C_6H_2$-2,6-Me_2-4-(CHCHBCat))$_2$(NMe_2)$_2$, 10** in CD_2Cl_2.

IR spectra

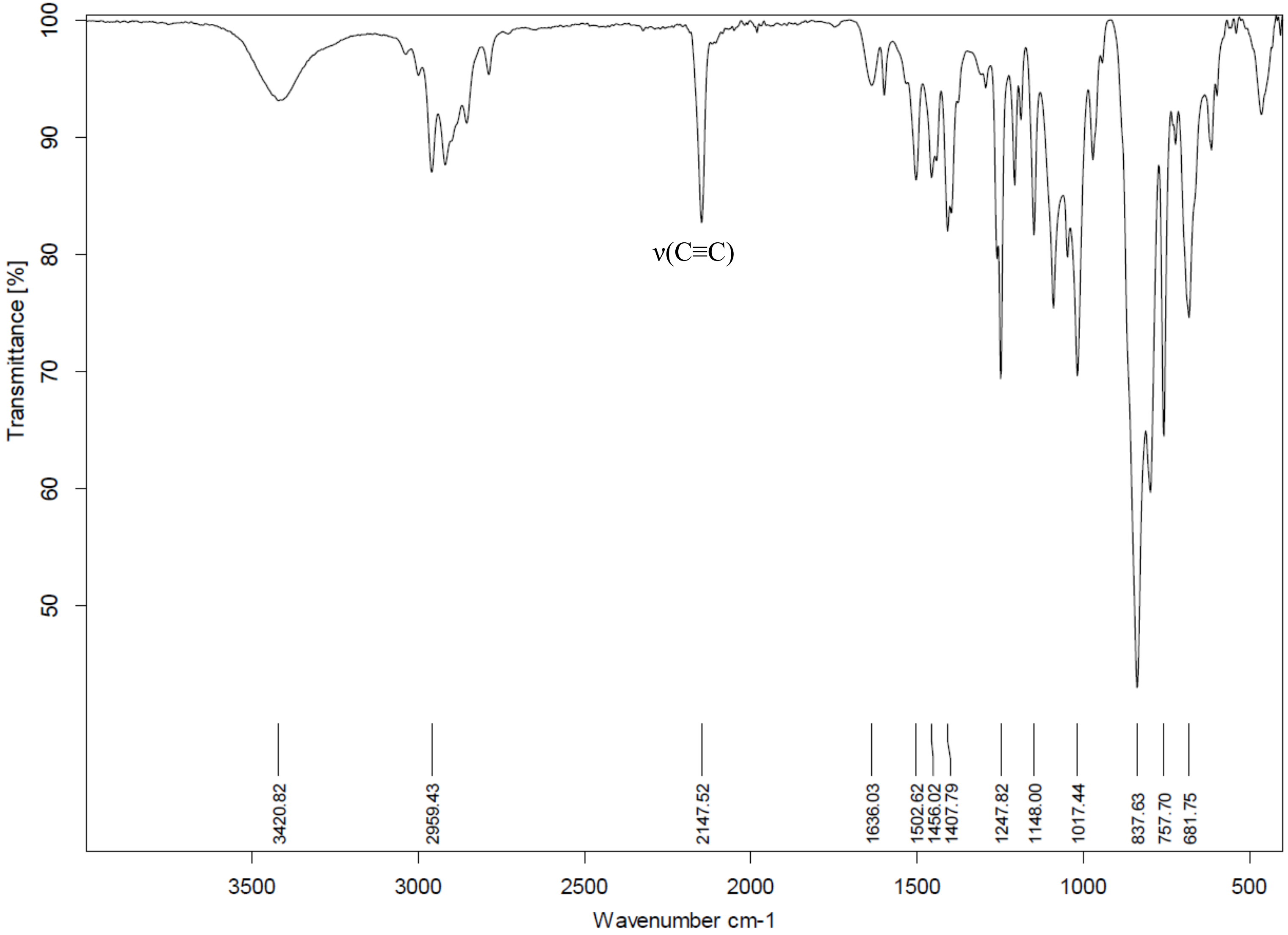

Figure S38. Solid-state IR spectrum of **$B_2(C_6H_2$-2,6-Me_2-4-$(CCSiMe_3))_2(NMe_2)_2$**, **1^{TMS}**.

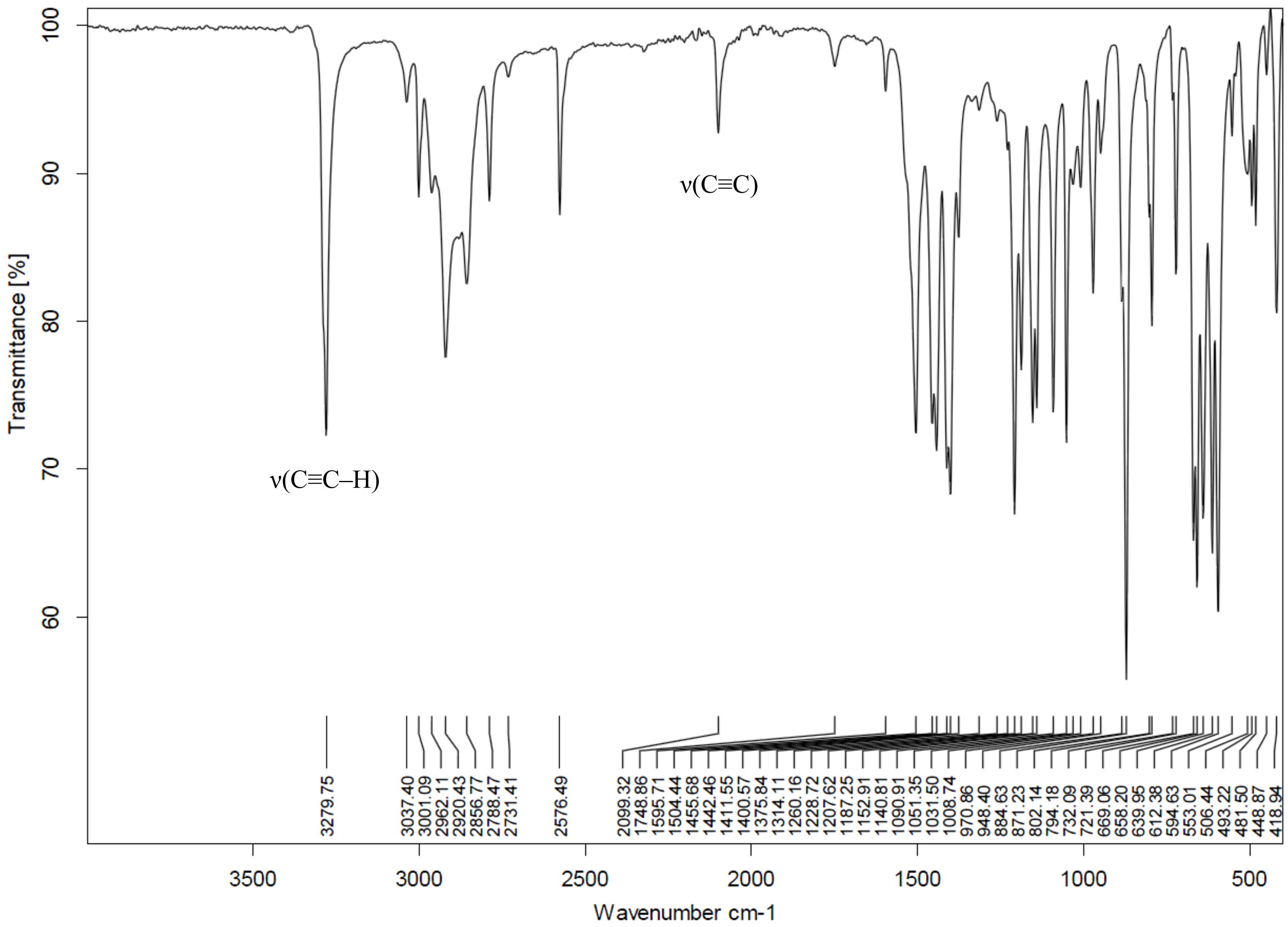

Figure S39. Solid-state IR spectrum of $\mathbf{B_2(C_6H_2\text{-}2,6\text{-}Me_2\text{-}4\text{-}(CCH))_2(NMe_2)_2}$, **1**.

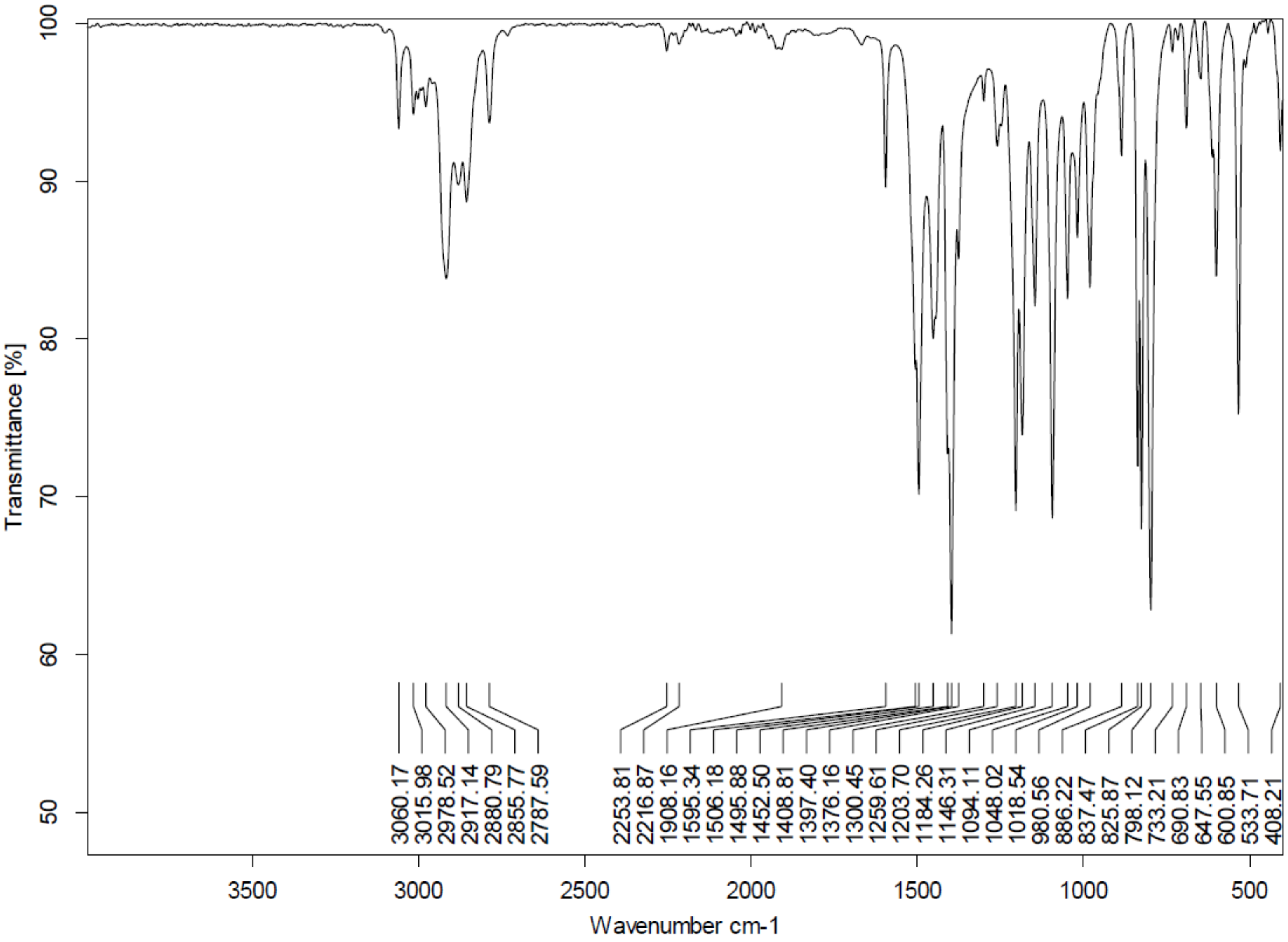

Figure S40. Solid-state IR spectrum of $\mathbf{B_2(C_6H_4\text{-}4\text{-}CCMe))_2(NMe_2)_2}$, **2**.

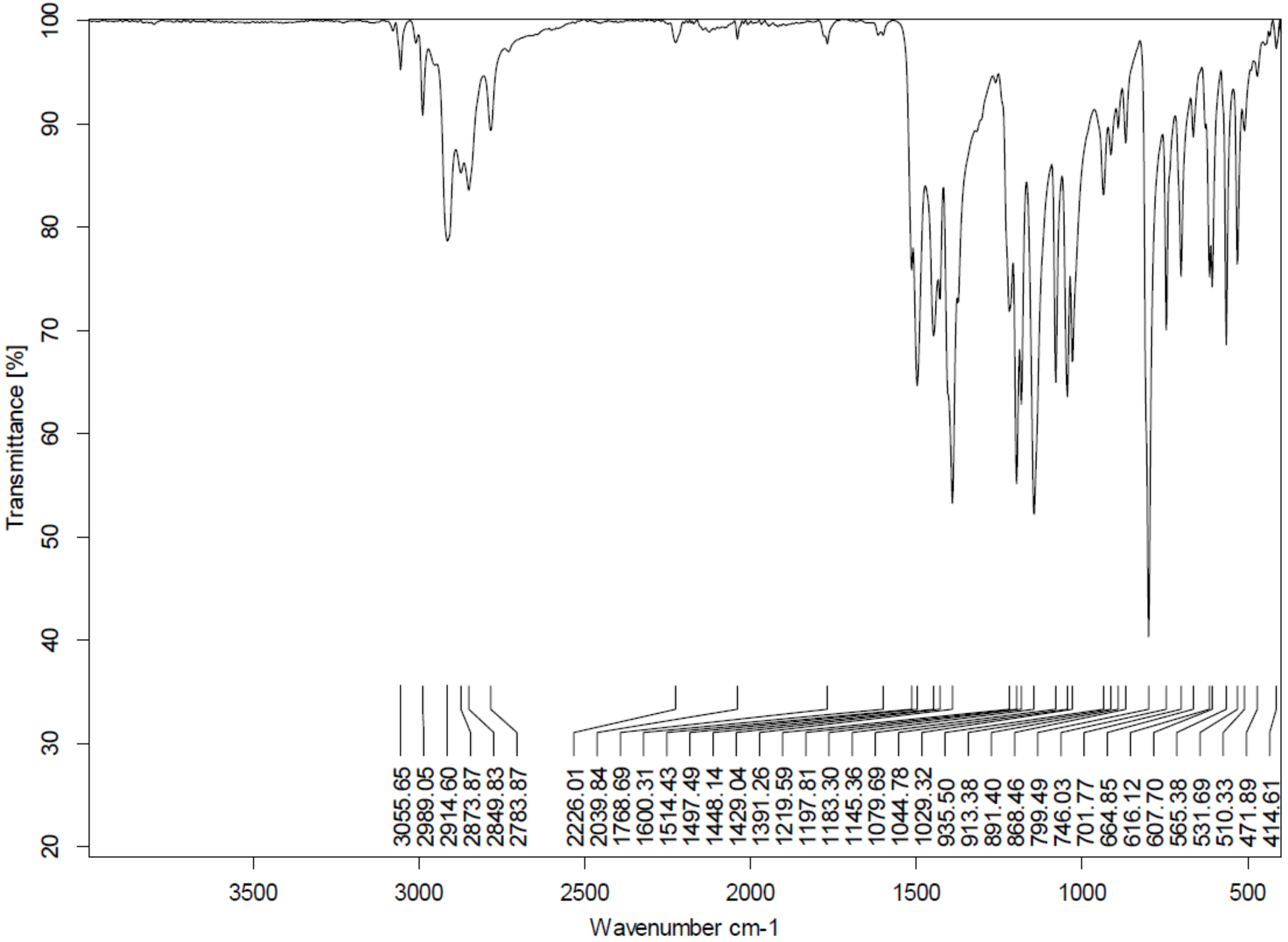

Figure S41. Solid-state IR spectrum of **$B_2(2\text{-}C_4H_2S\text{-}5\text{-}(CCMe))_2(NMe_2)_2$**, **3**.

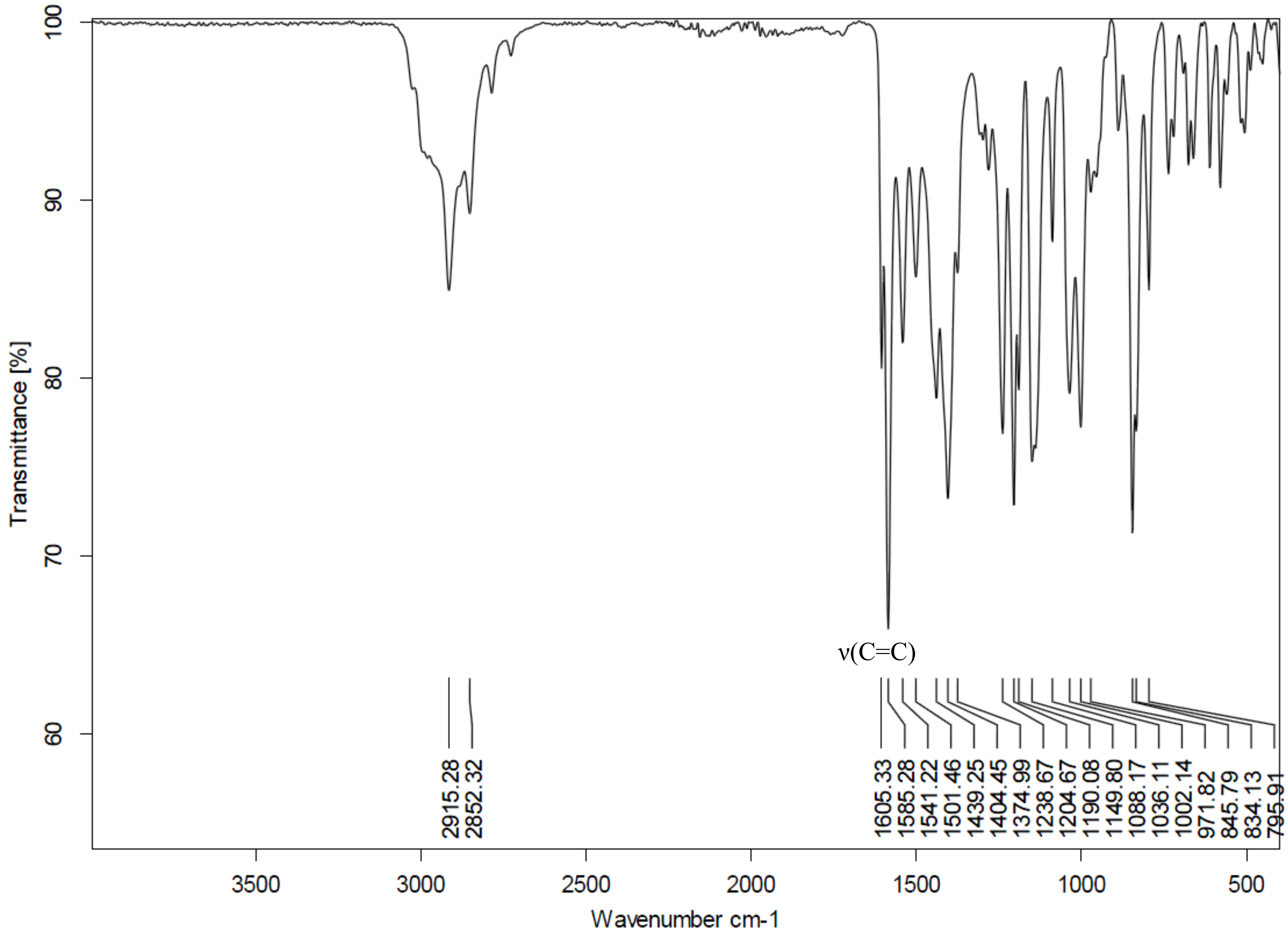

Figure S42. Solid-state IR spectrum of **$B_2(C_6H_2$-2,6-Me_2-4-$(CHCHBMes_2))_2(NMe_2)_2$**, **5a**.

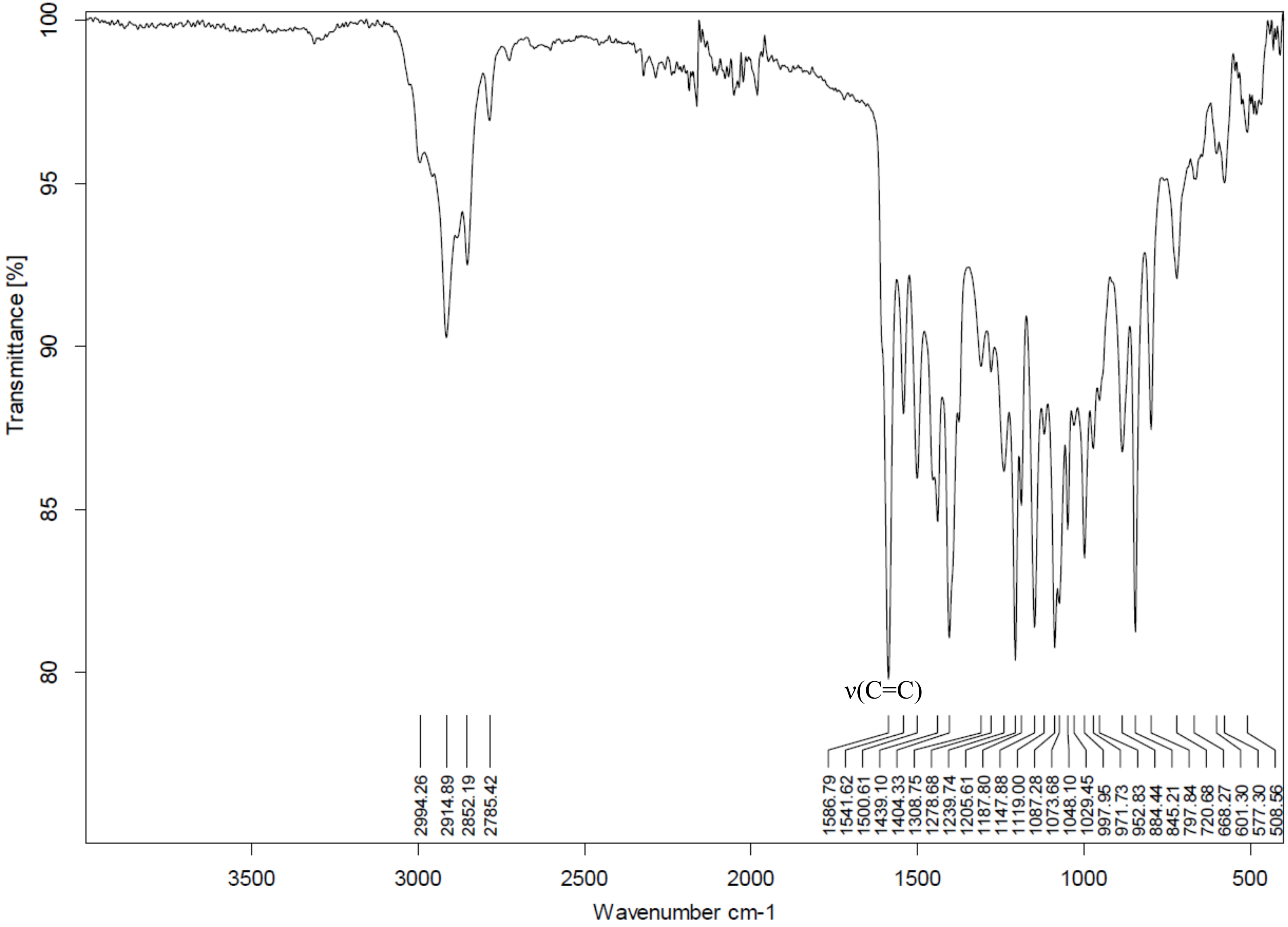

Figure S43. Solid-state IR spectrum of **Oligomerization product of 1**, **5b**.

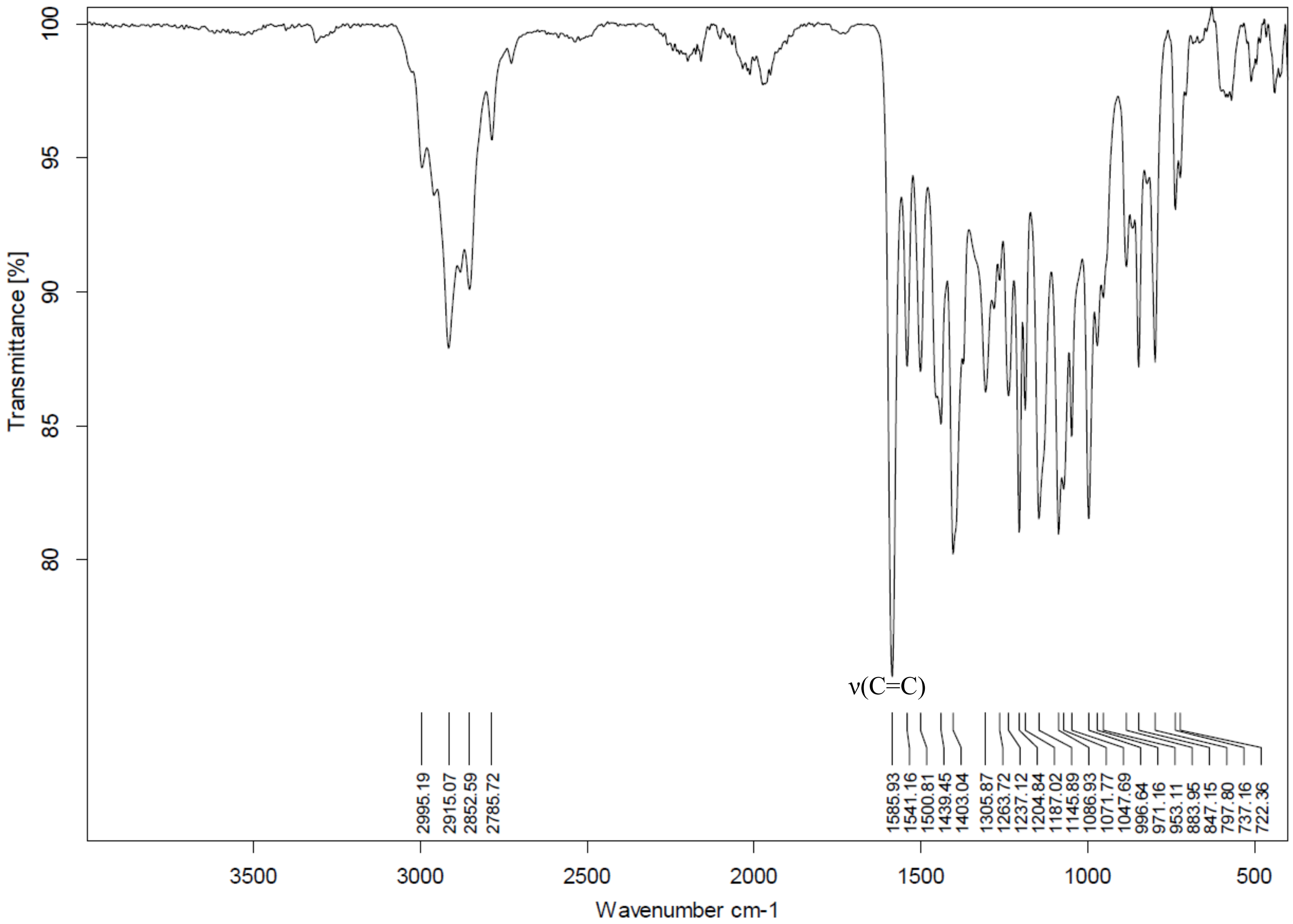

Figure S44. Solid-state IR spectrum of **Oligomerization product of 1**, **5c**.

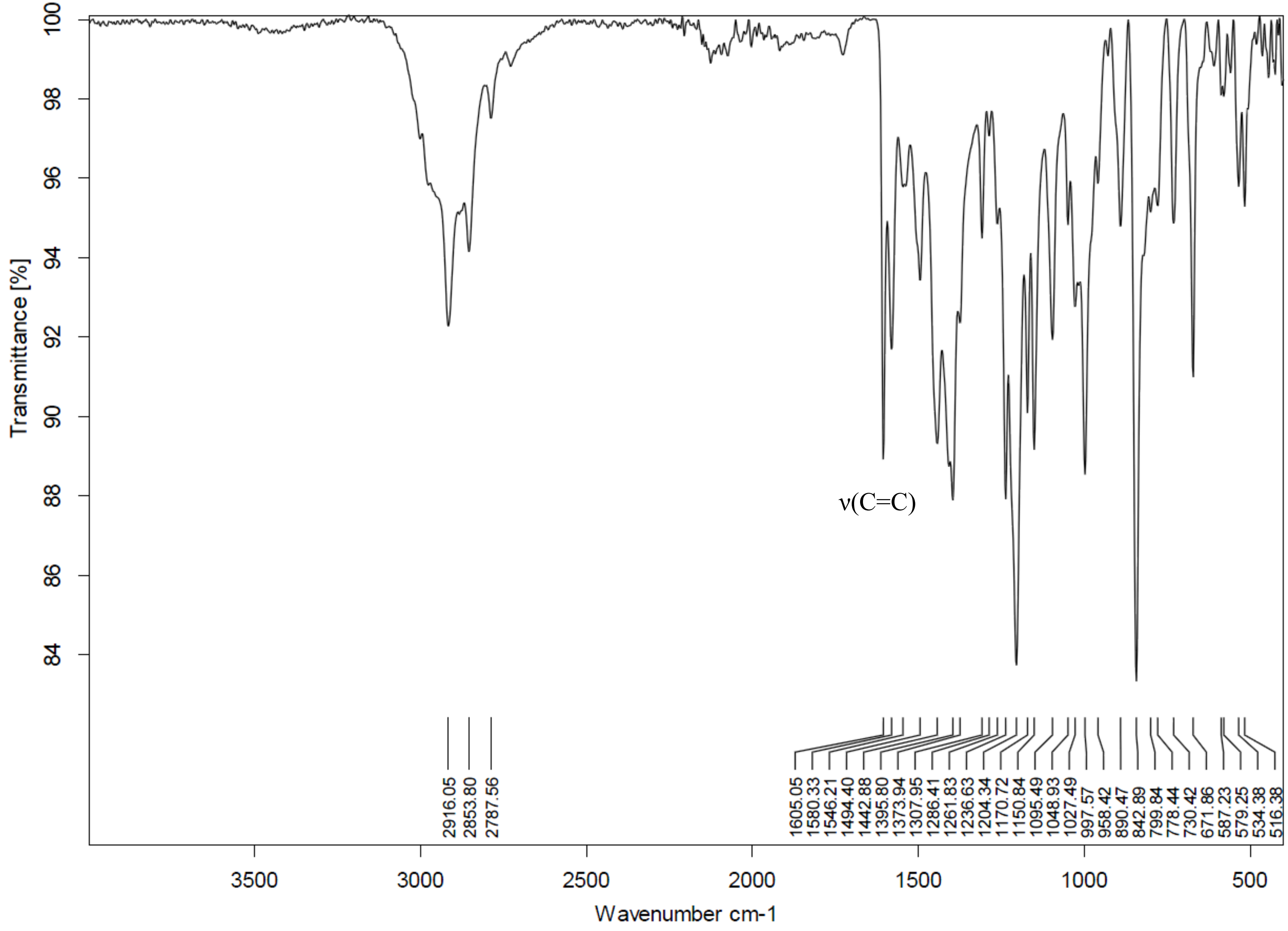

Figure S45. Solid-state IR spectrum of $\mathbf{B_2(C_6H_4\text{-}4\text{-}(CHCMeBMes_2))_2(NMe_2)_2}$, **6a**.

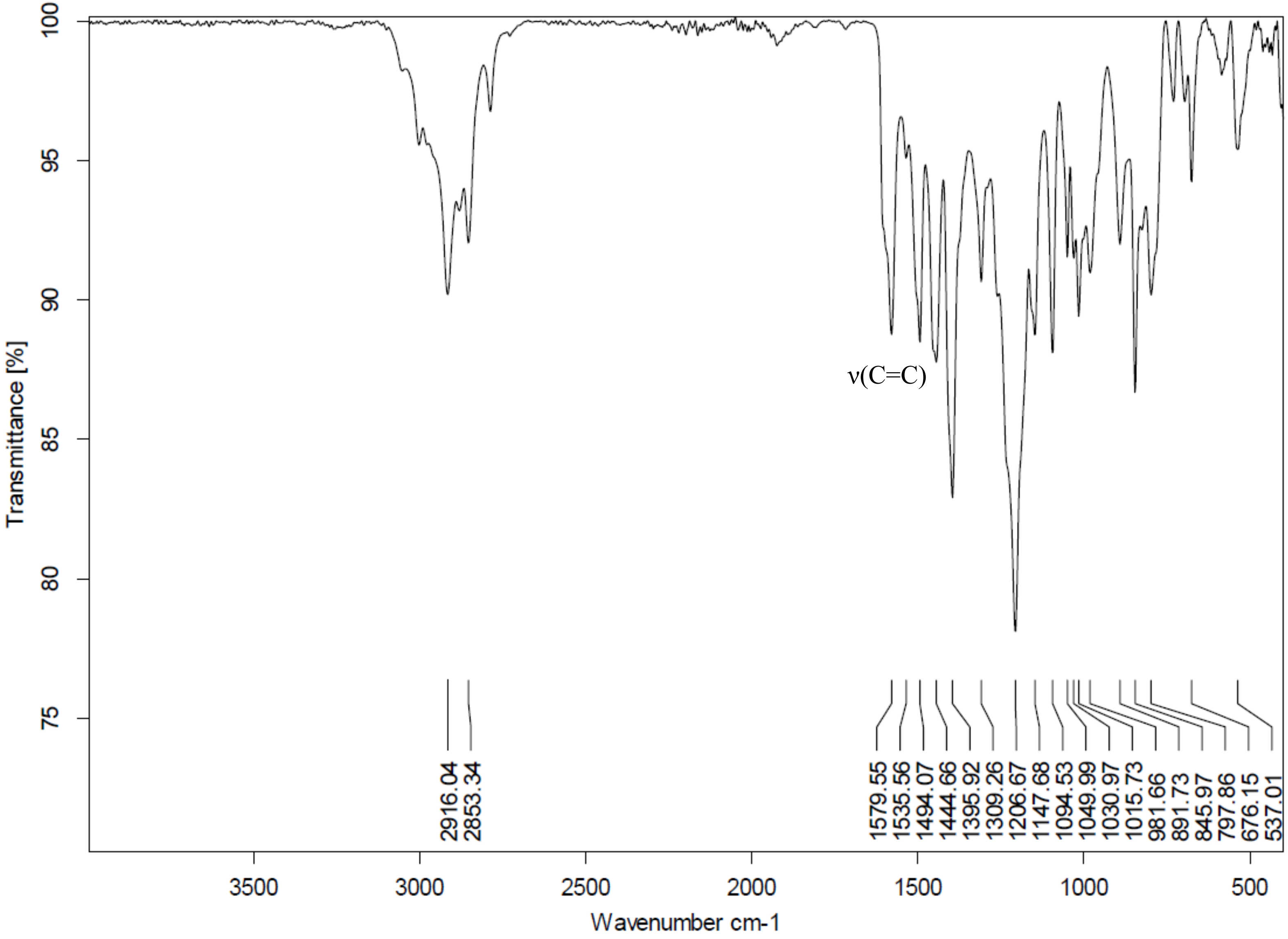

Figure S46. Solid-state IR spectrum of **Oligomerization product of 2**, **6b**.

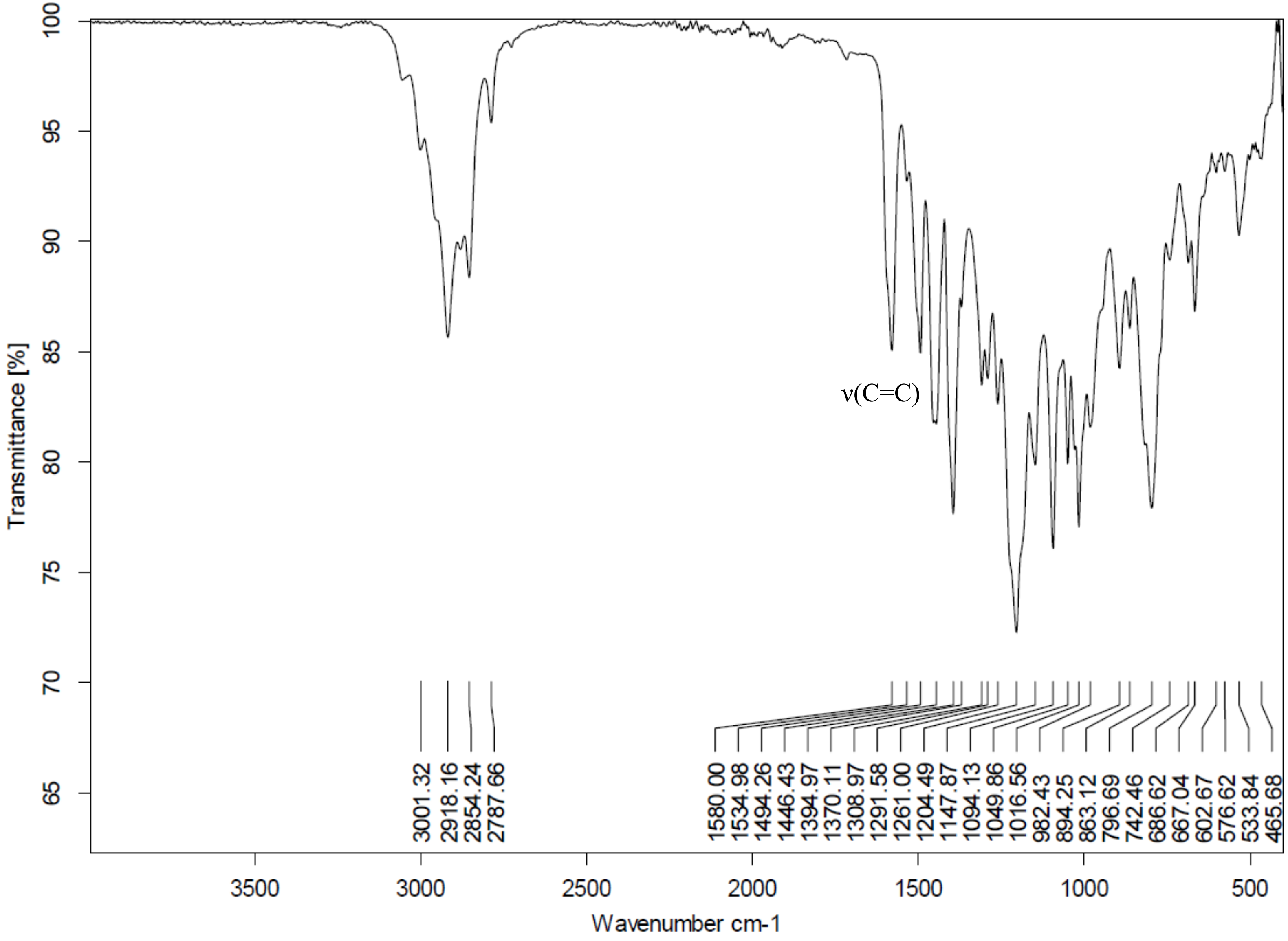

Figure S47. Solid-state IR spectrum of **Oligomerization product of 2**, **6c**.

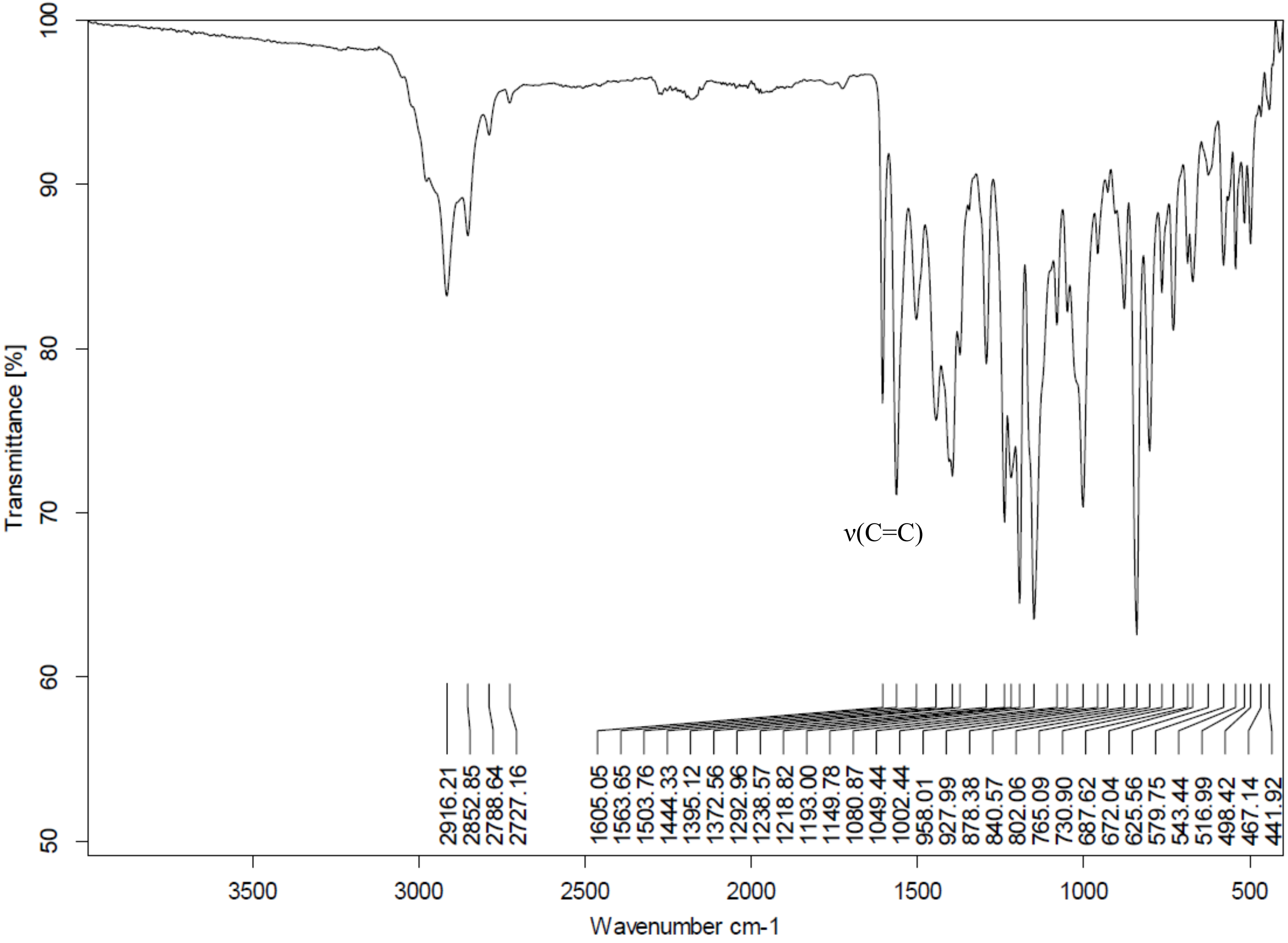

Figure S48. Solid-state IR spectrum of $\mathbf{B_2(2\text{-}C_4H_2S\text{-}5\text{-}(CHCMeBMes_2))_2(NMe_2)_2}$, **7a**.

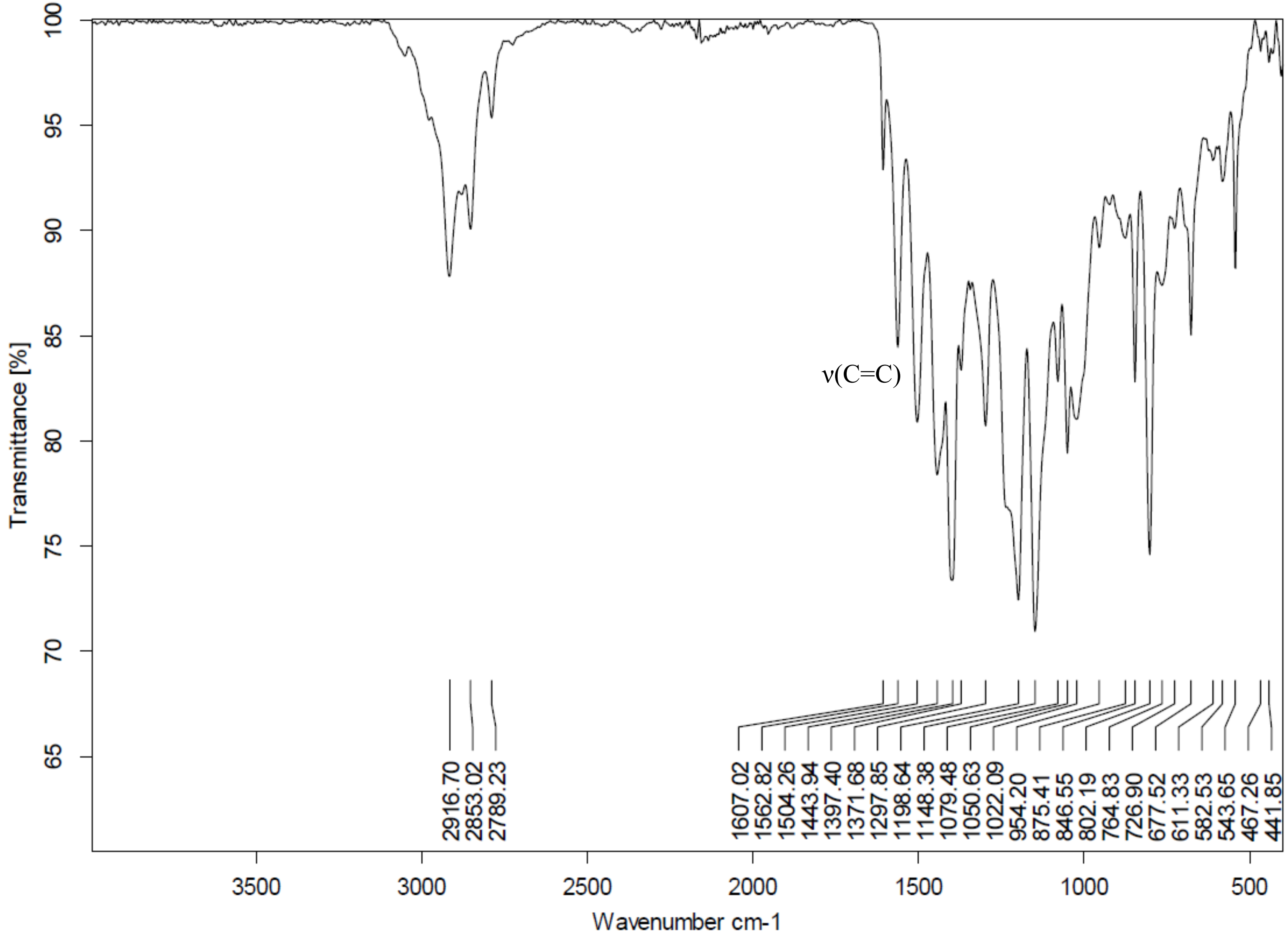

Figure S49. Solid-state IR spectrum of **Oligomerization product of 3**, **7b**.

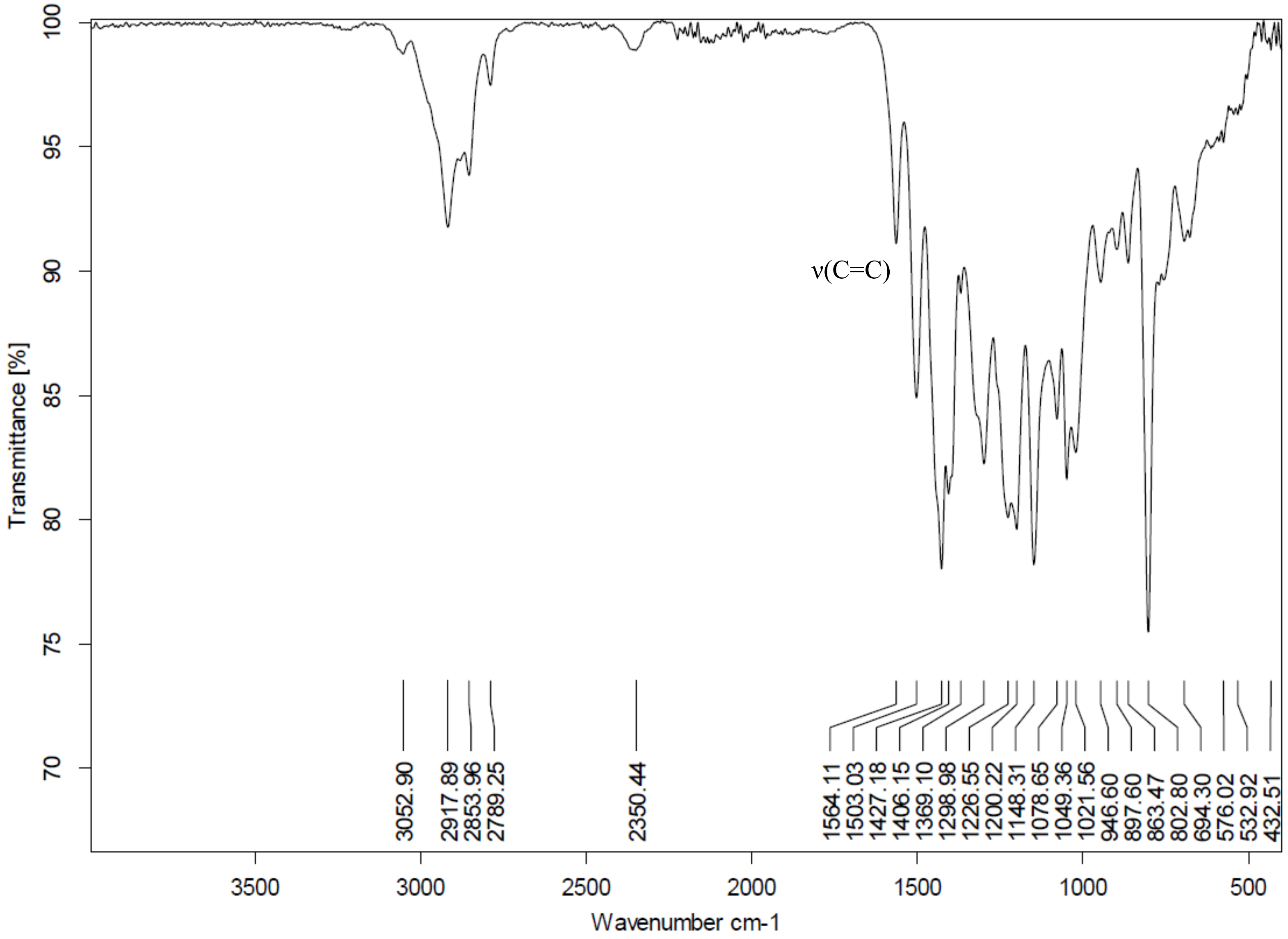

Figure S50. Solid-state IR spectrum of **Oligomerization product of 3**, **7c**.

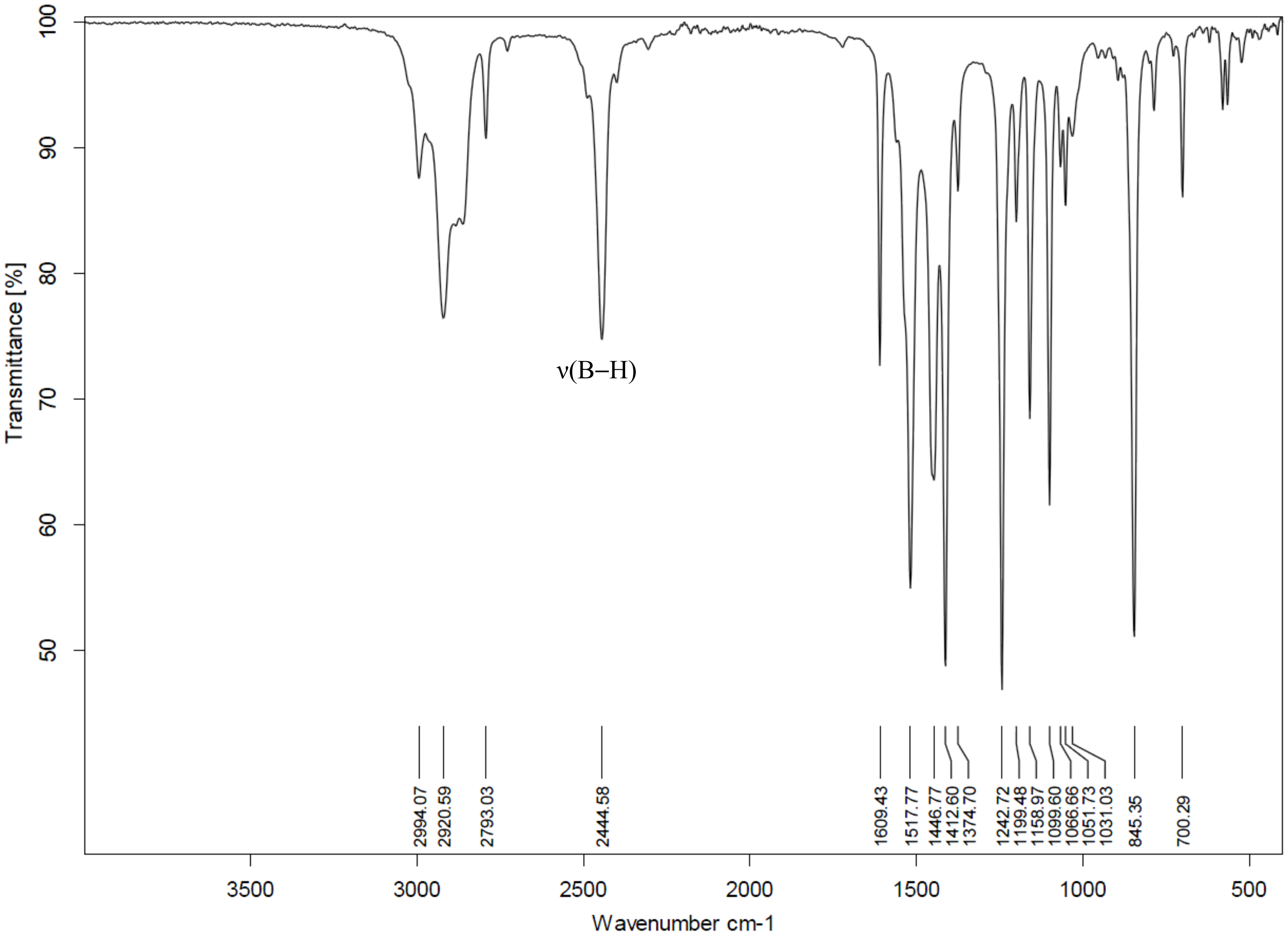

Figure S51. IR spectrum of **HBMes(NMe$_2$)**, **8**.

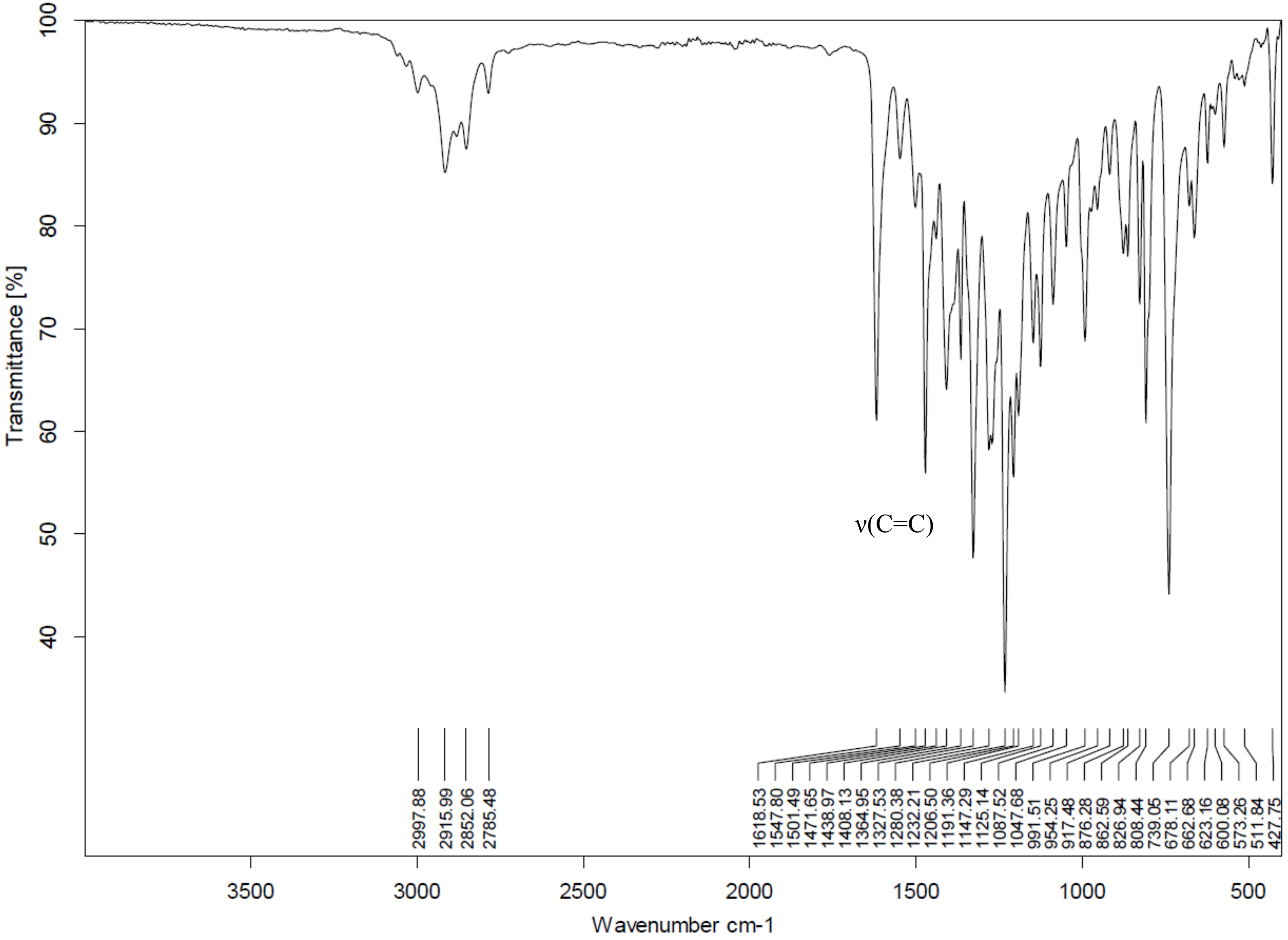

Figure S52. Solid-state IR spectrum of **$B_2(C_6H_2$-2,6-Me_2-4-(CHCHBCat))$_2(NMe_2)_2$, 10**.

Raman spectra

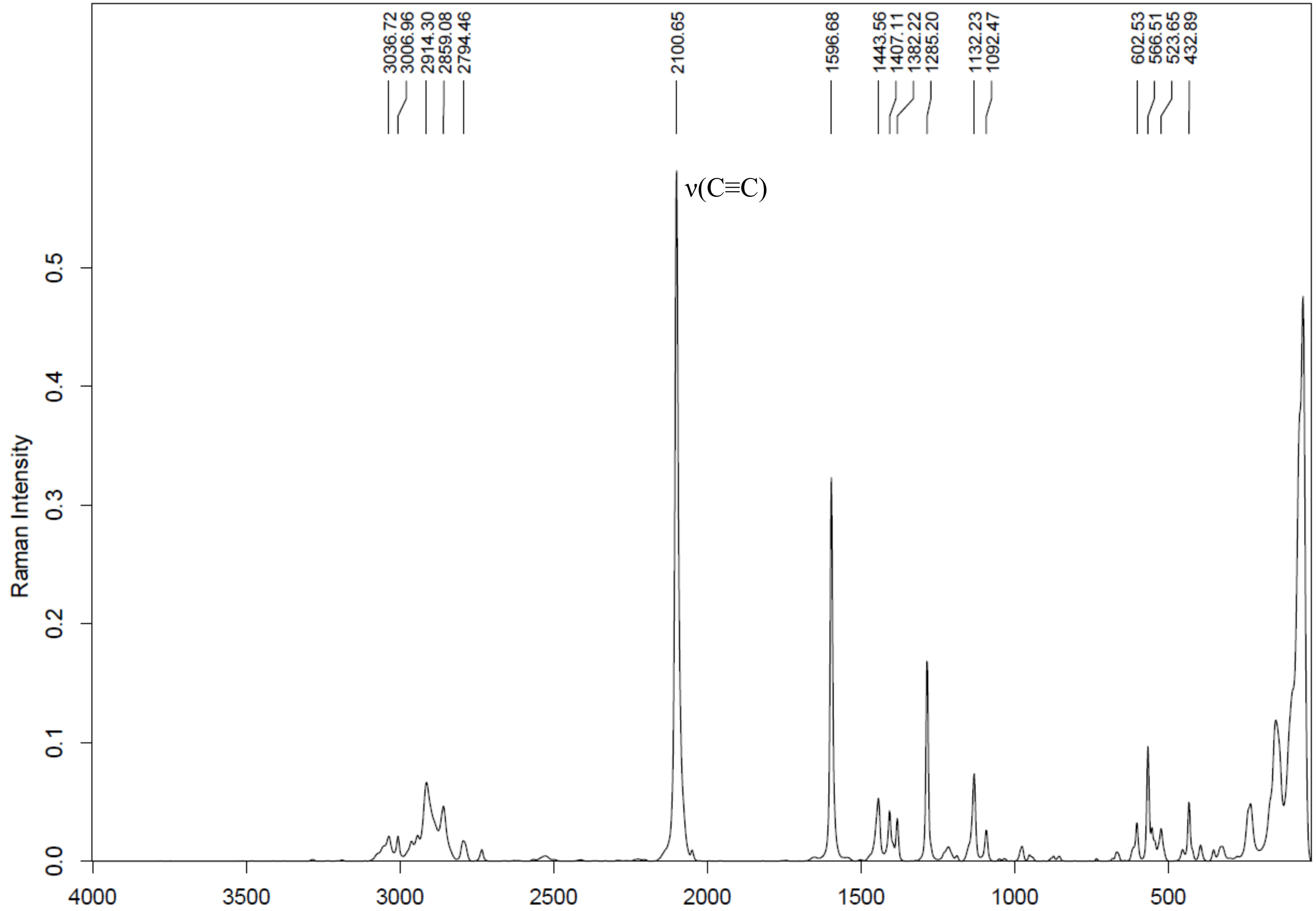

Figure S53. Solid-state IR spectrum of **$B_2(C_6H_2$-2,6-Me_2-4-(CCH)$)_2(NMe_2)_2$**, **1**.

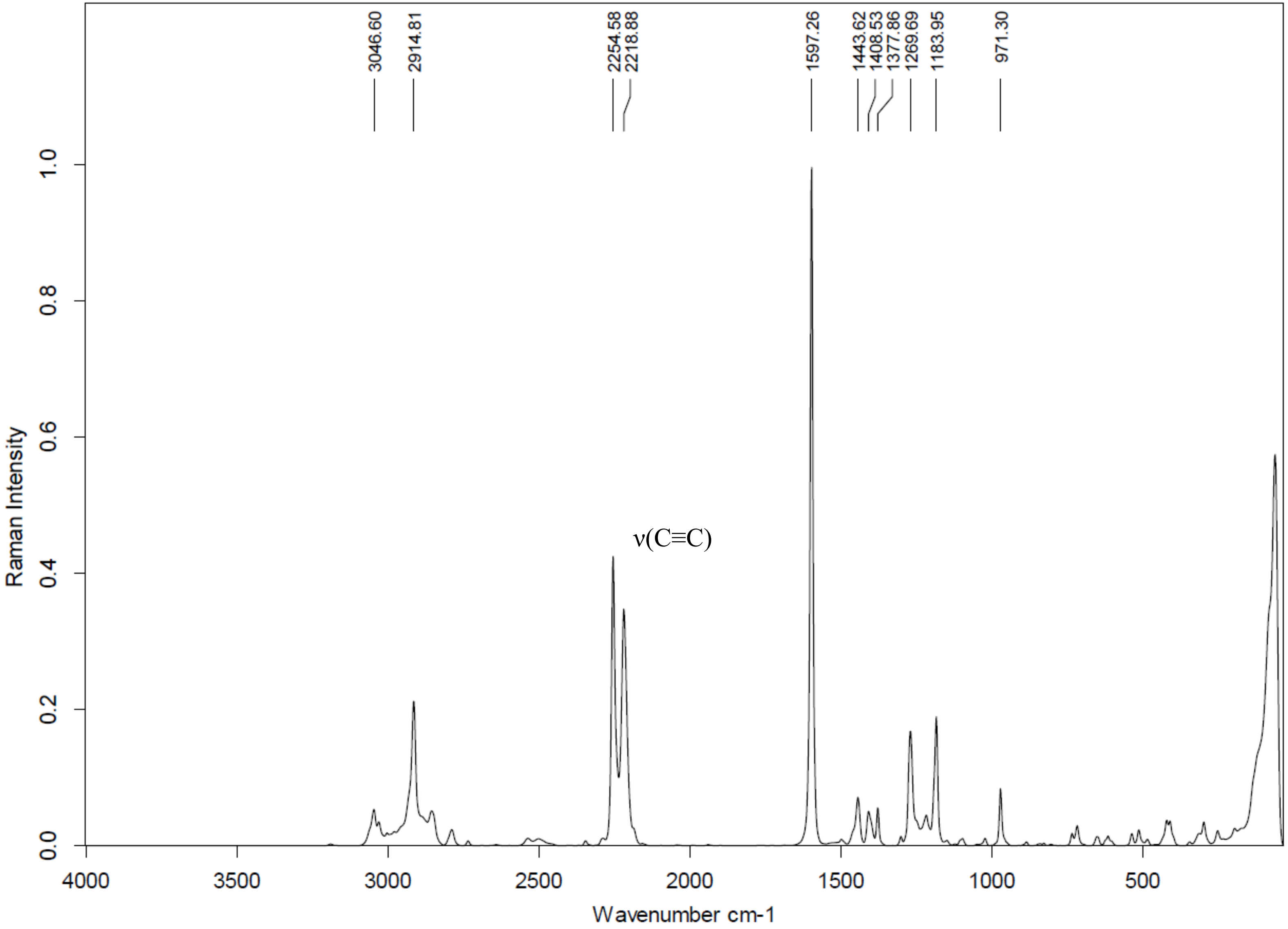

Figure S54. Solid-state Raman spectrum of $\mathbf{B_2(C_6H_4\text{-}4\text{-}CCMe))_2(NMe_2)_2}$, **2**.

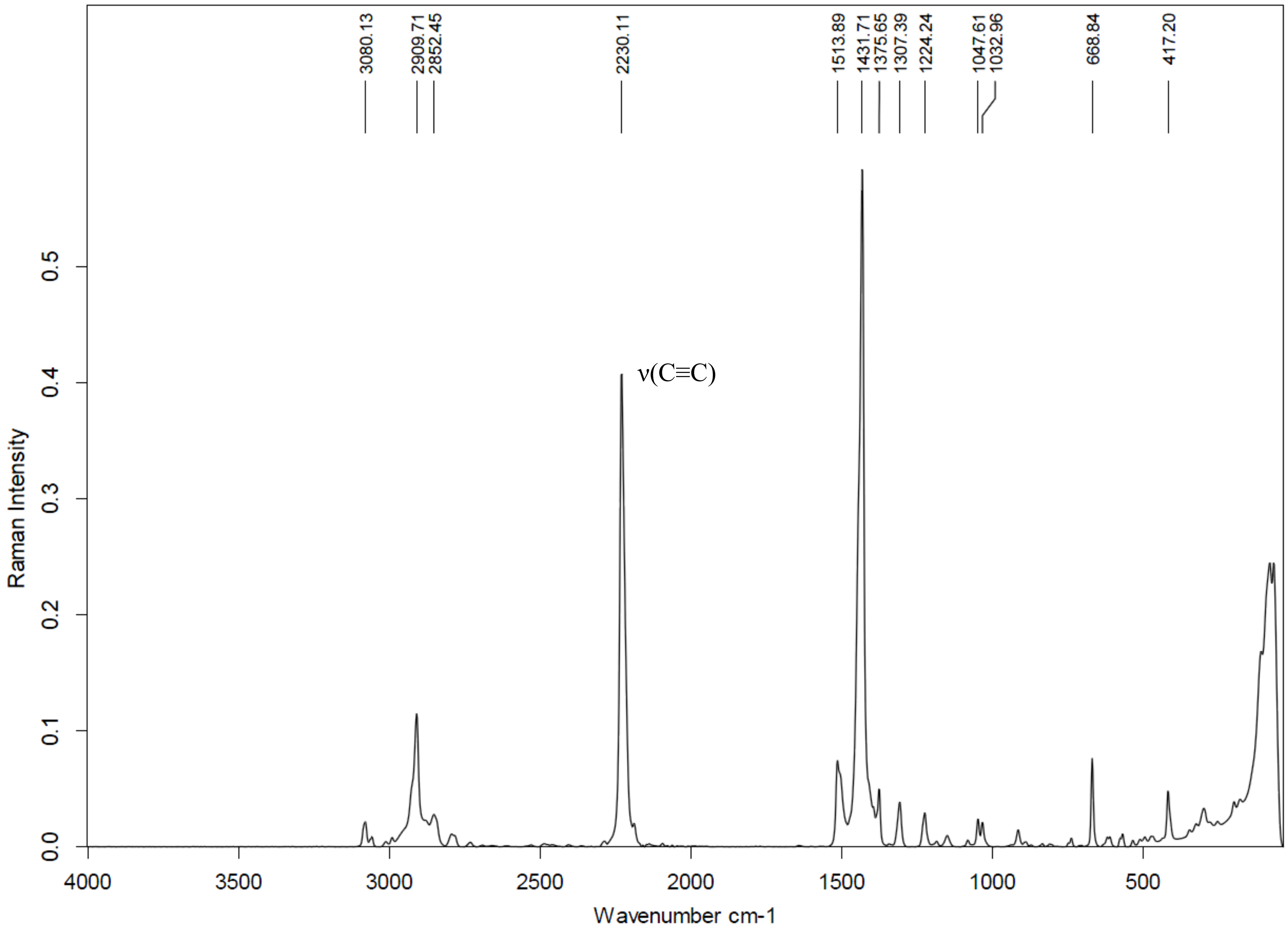

Figure S55. Solid-state Raman spectrum of **B_2(2-C_4H_2S-5-(CCMe))$_2$(NMe_2)$_2$**, **3**.

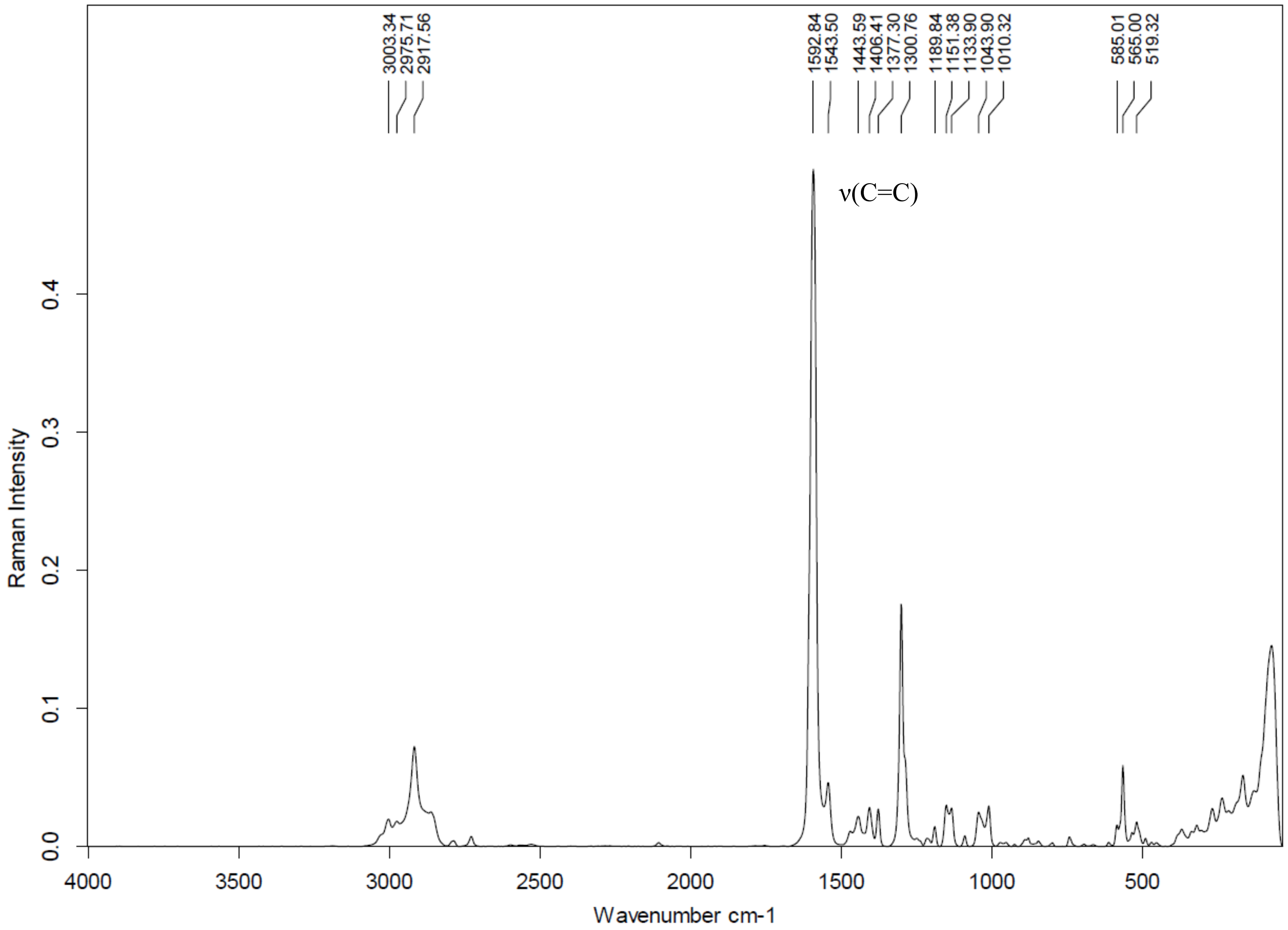

Figure S56. Solid-state Raman spectrum of **$B_2(C_6H_2$-2,6-Me_2-4-($CHCHBMes_2$))$_2$(NMe_2)$_2$**, **5a**.

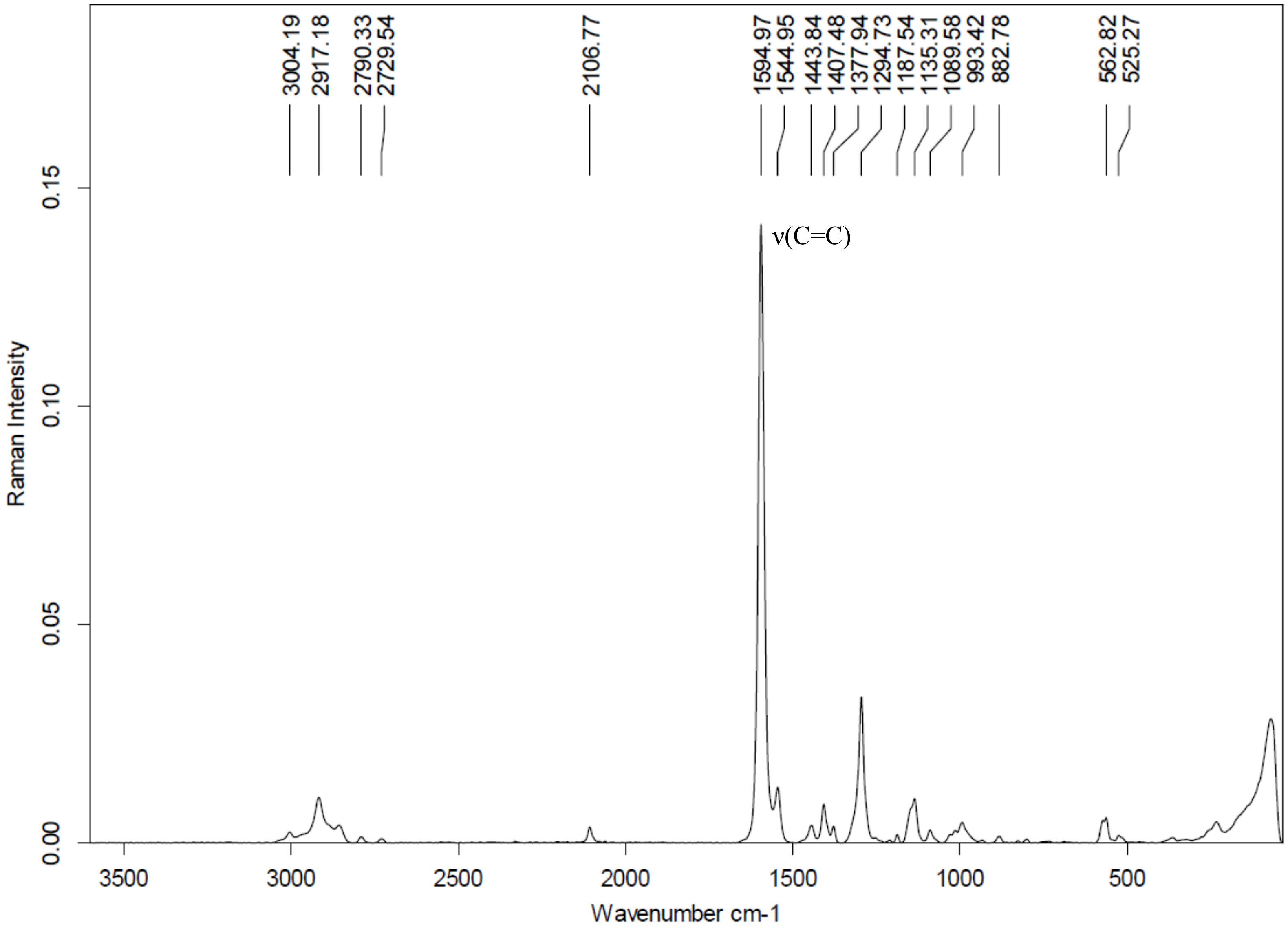

Figure S57. Solid-state Raman spectrum of **Oligomerization product of 1**, **5b**.

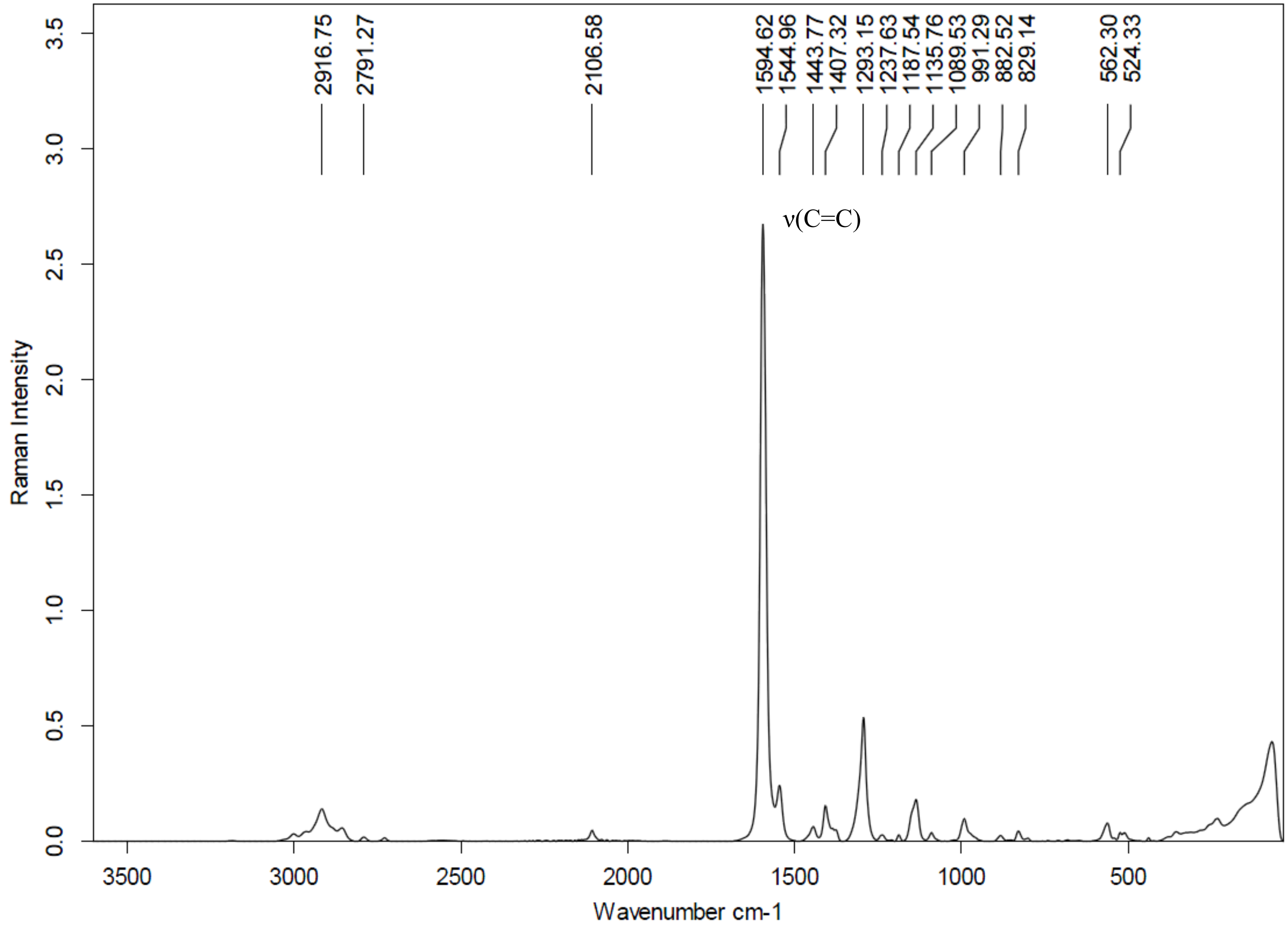

Figure S58. Solid-state Raman spectrum of **Oligomerization product of 1**, **5c**.

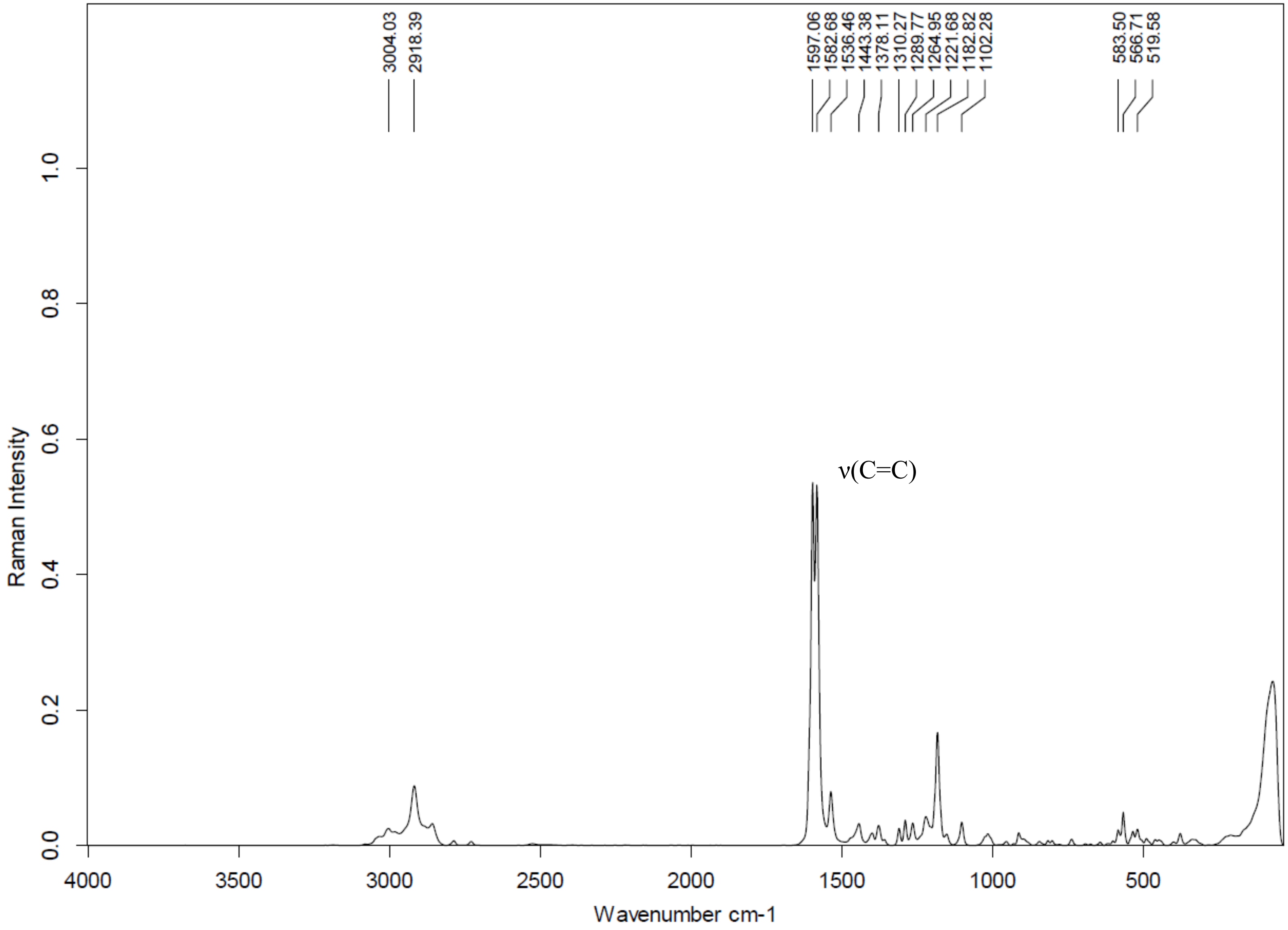

Figure S59. Solid-state Raman spectrum of **$B_2(C_6H_4$-4-$(CHCMeBMes_2))_2(NMe_2)_2$**, **6a**.

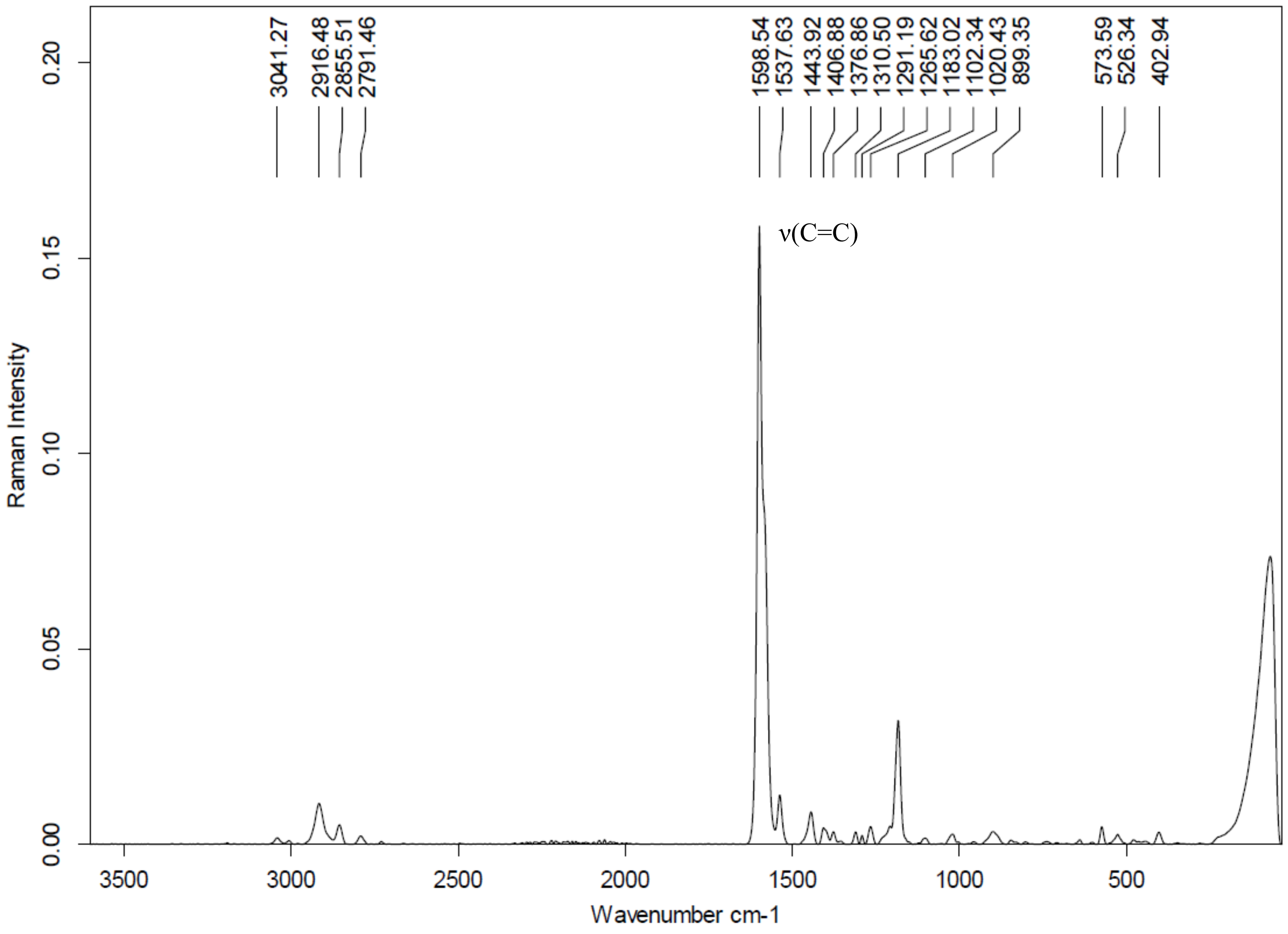

Figure S60. Solid-state Raman spectrum of **Oligomerization product of 2**, **6b**.

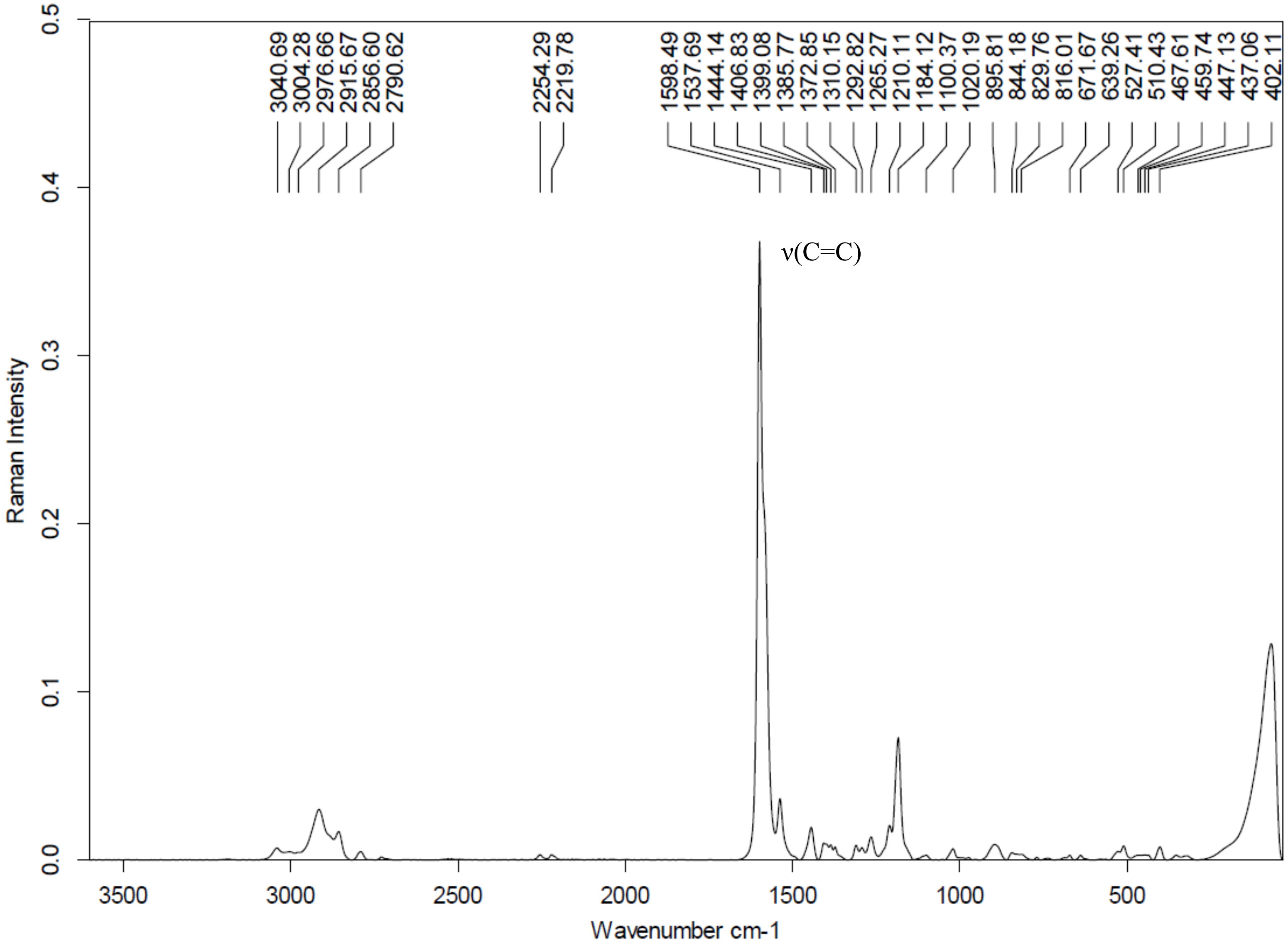

Figure S61. Solid-state Raman spectrum of **Oligomerization product of 2**, **6c**.

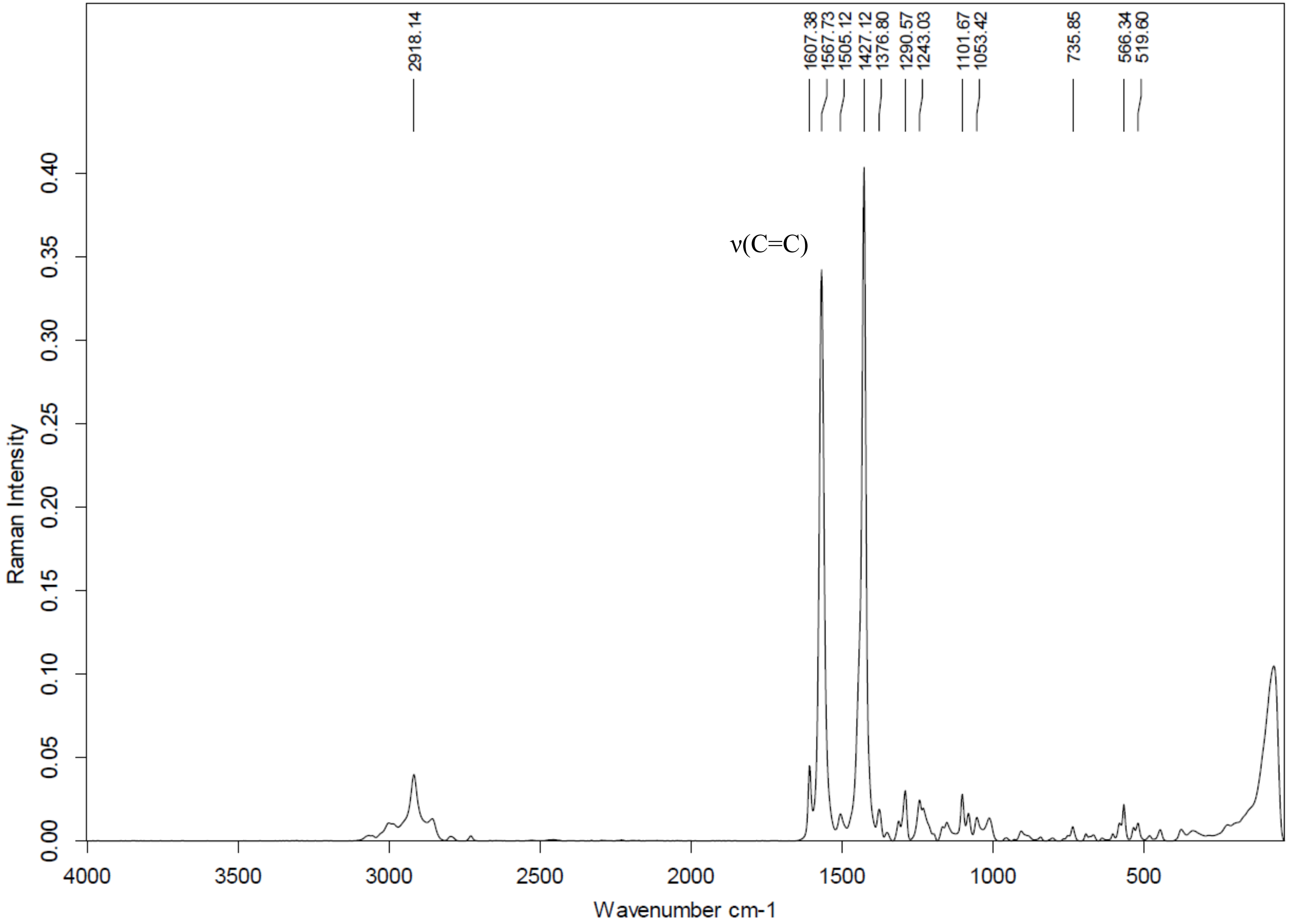

Figure S62. Solid-state Raman spectrum of $\mathbf{B_2(2\text{-}C_4H_2S\text{-}5\text{-}(CHCMeBMes_2))_2(NMe_2)_2}$, **7a**.

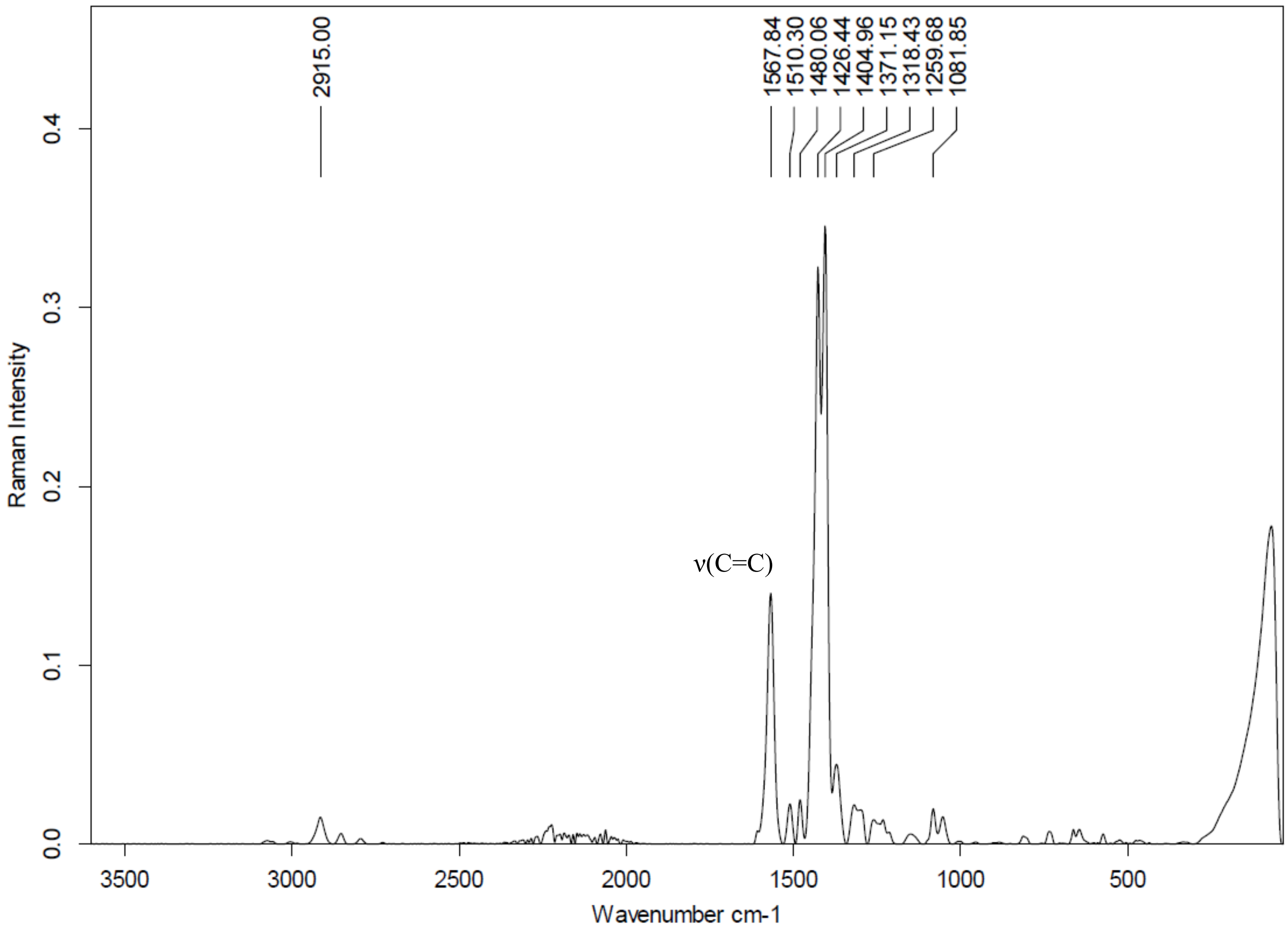

Figure S63. Solid-state Raman spectrum of **Oligomerization product of 3**, **7b**.

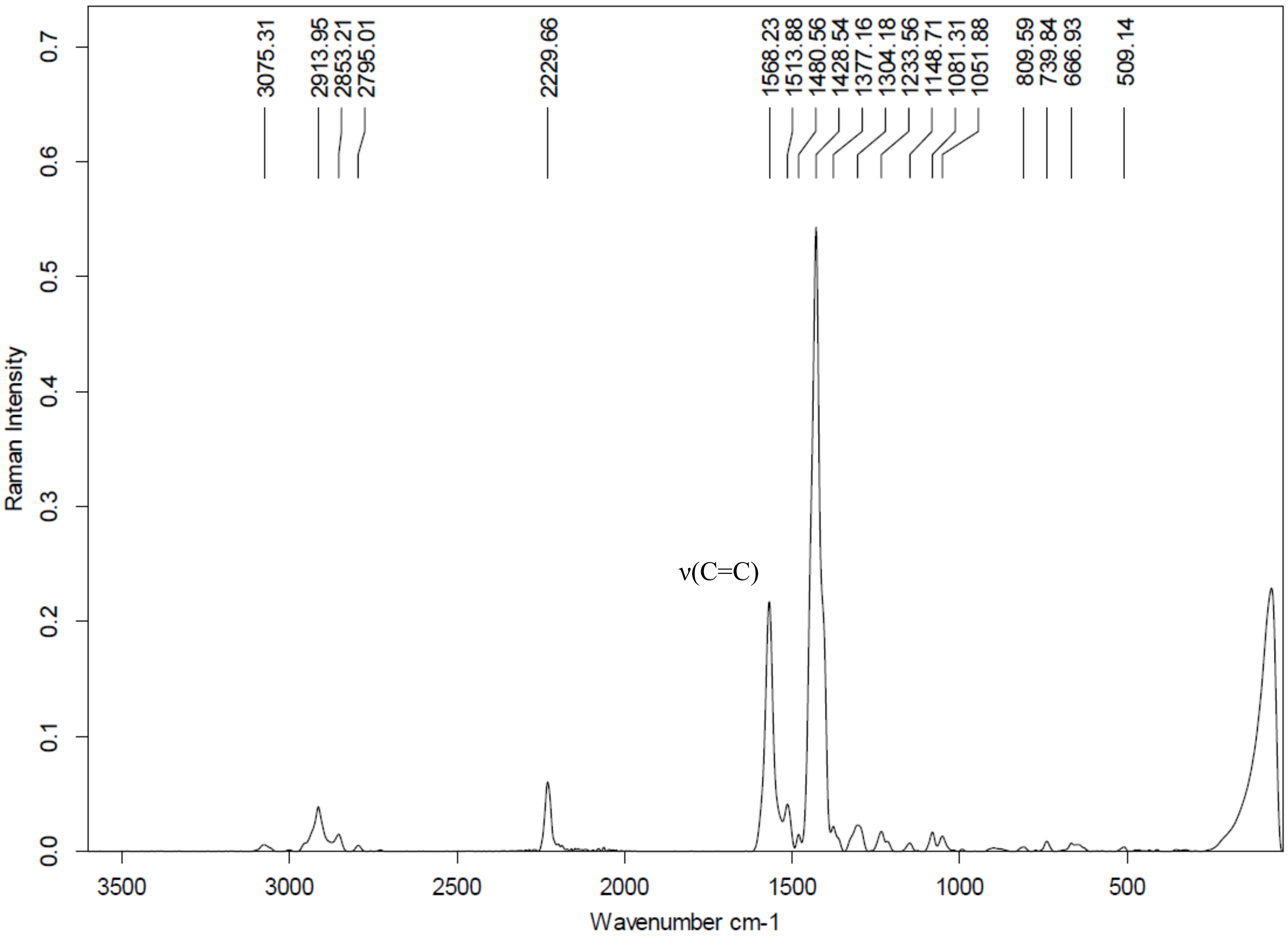

Figure S64. Solid-state Raman spectrum of **Oligomerization product of 3**, **7c**.

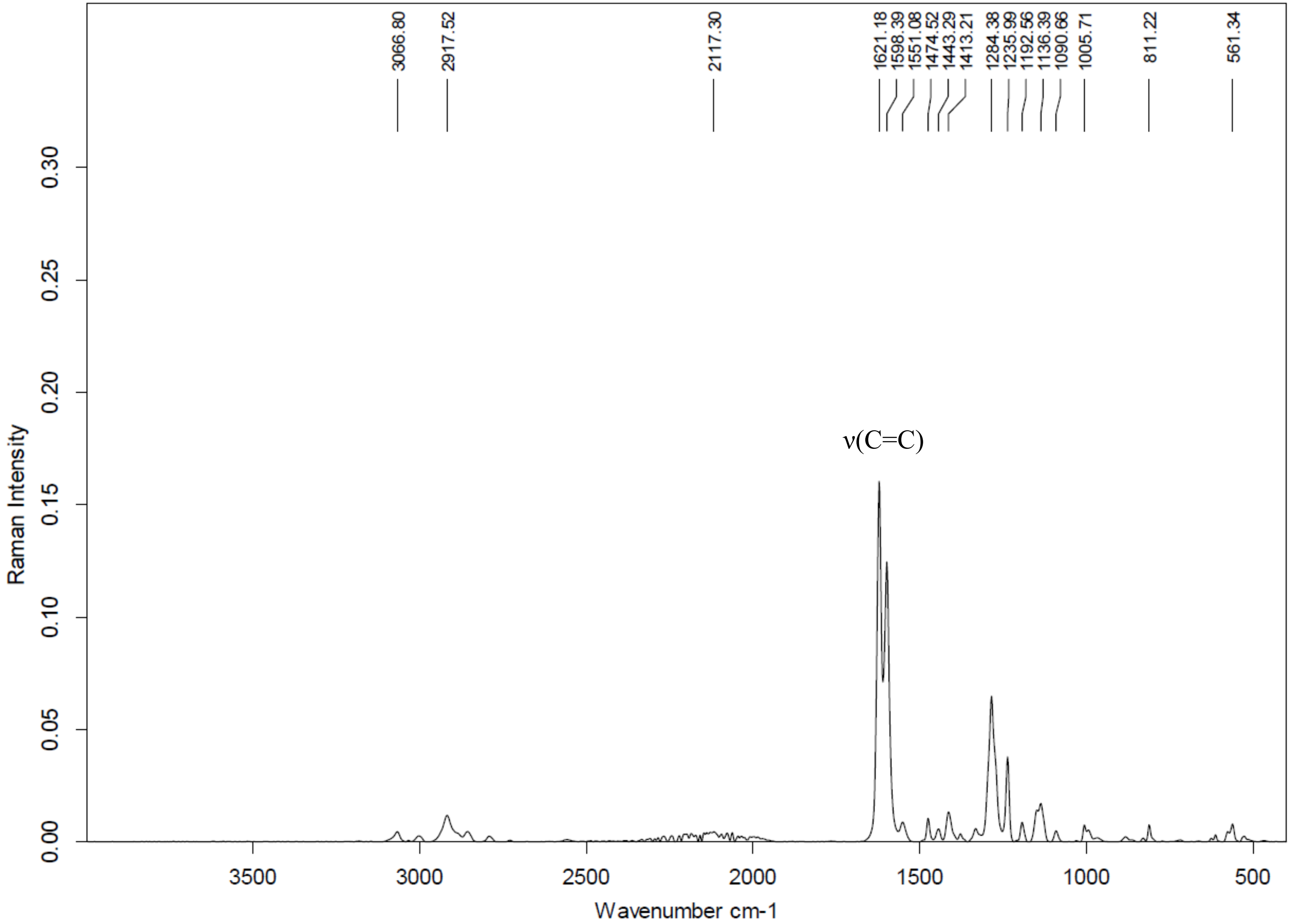

Figure S65. Solid-state Raman spectrum of **$B_2(C_6H_2$-2,6-Me_2-4-(CHCHBCat))$_2(NMe_2)_2$, 10**.

Size exclusion chromatography (SEC)

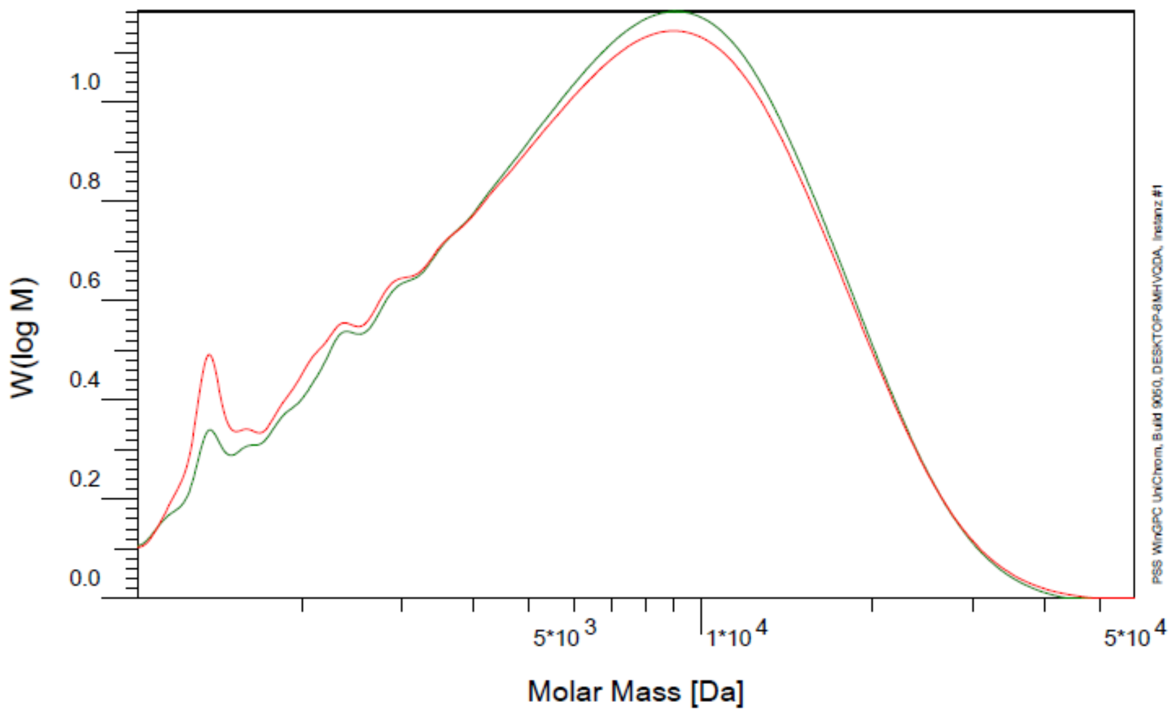

Figure S66. SEC trace of **Oligomerization product of 1**, **5b** (red line: detection by UV signal; green line: detection by RI signal).

Table S1. Data of the SEC analysis of **Oligomerization product of 1**, **5b**.

	**RI**	**UV**
$\boldsymbol{M_n}$	4933 g/mol	4715 g/mol
$\boldsymbol{M_w}$	8615 g/mol	8487 g/mol
$\boldsymbol{M_z}$	12955 g/mol	13089 g/mol
PDI	1.75	1.80

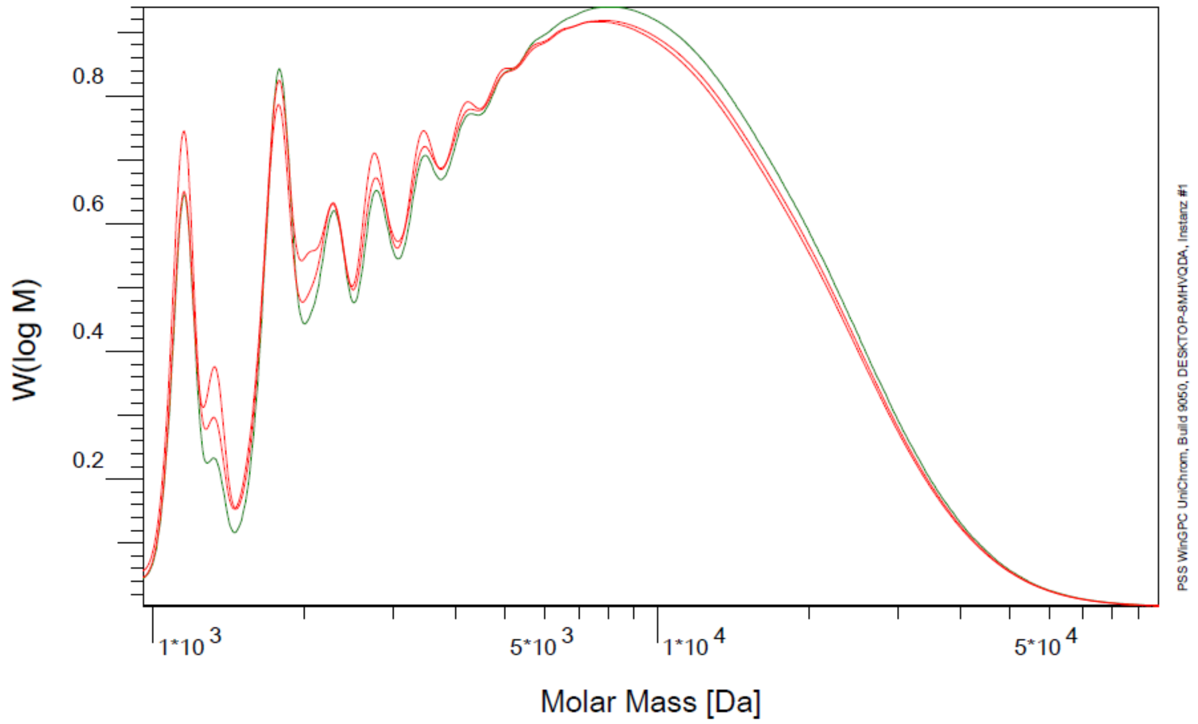

Figure S67. SEC trace of **Oligomerization product of 1**, **5c** (red lines: detection by UV signal; green line: detection by RI signal).

Table S2. SEC analysis data for **Oligomerization product of 1**, **5c**.

	**RI**	**UV**
$\boldsymbol{M_n}$	4502 g/mol	4298 g/mol
$\boldsymbol{M_w}$	9820 g/mol	9467 g/mol
$\boldsymbol{M_z}$	18399 g/mol	18177 g/mol
PDI	2.18	2.20

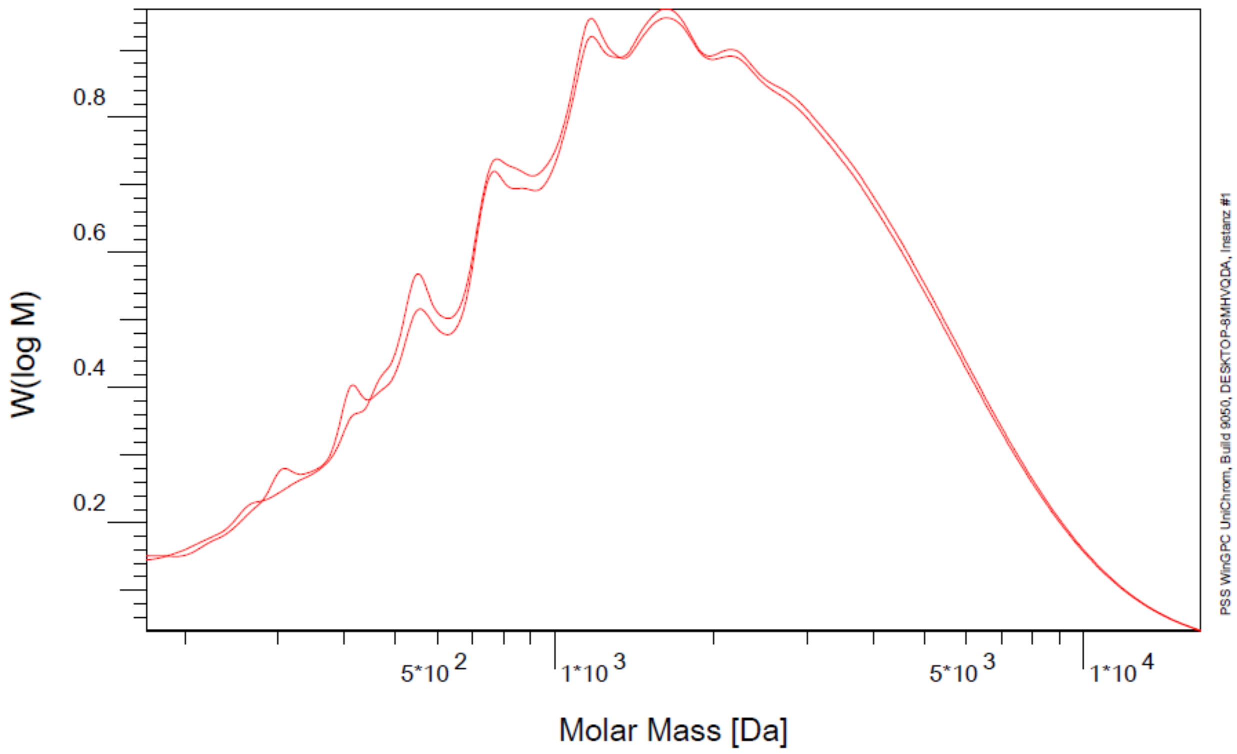

Figure S68. SEC trace of **Oligomerization product of 2**, **6b** (red line 1: detection by UV signal 1; red line 2: detection by UV signal 2).

Table S3. SEC analysis data for **Oligomerization product of 2**, **6b**.

	**UV 1**	**UV 2**
M_n	1045 g/mol	1037 g/mol
M_w	2473 g/mol	2443 g/mol
M_z	4806 g/mol	4780 g/mol
PDI	2.37	2.36

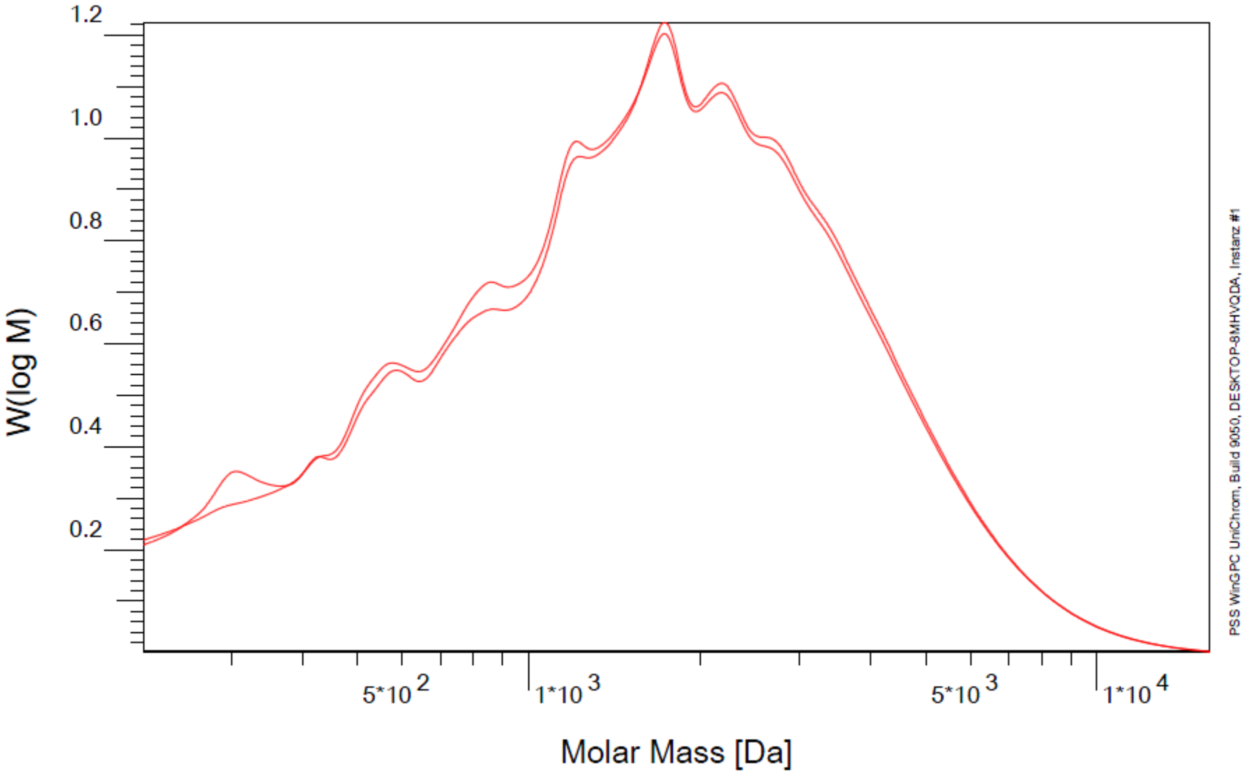

Figure S69. SEC trace of **Oligomerization product of 2**, **6c** (red line 1: detection by UV signal 1; red line 2: detection by UV signal 2).

Table S4. SEC analysis data for **Oligomerization product of 2**, **6c**.

	**UV 1**	**UV 2**
M_n	1047 g/mol	1049 g/mol
M_w	2088 g/mol	2071 g/mol
M_z	3510 g/mol	3499 g/mol
PDI	1.99	1.97

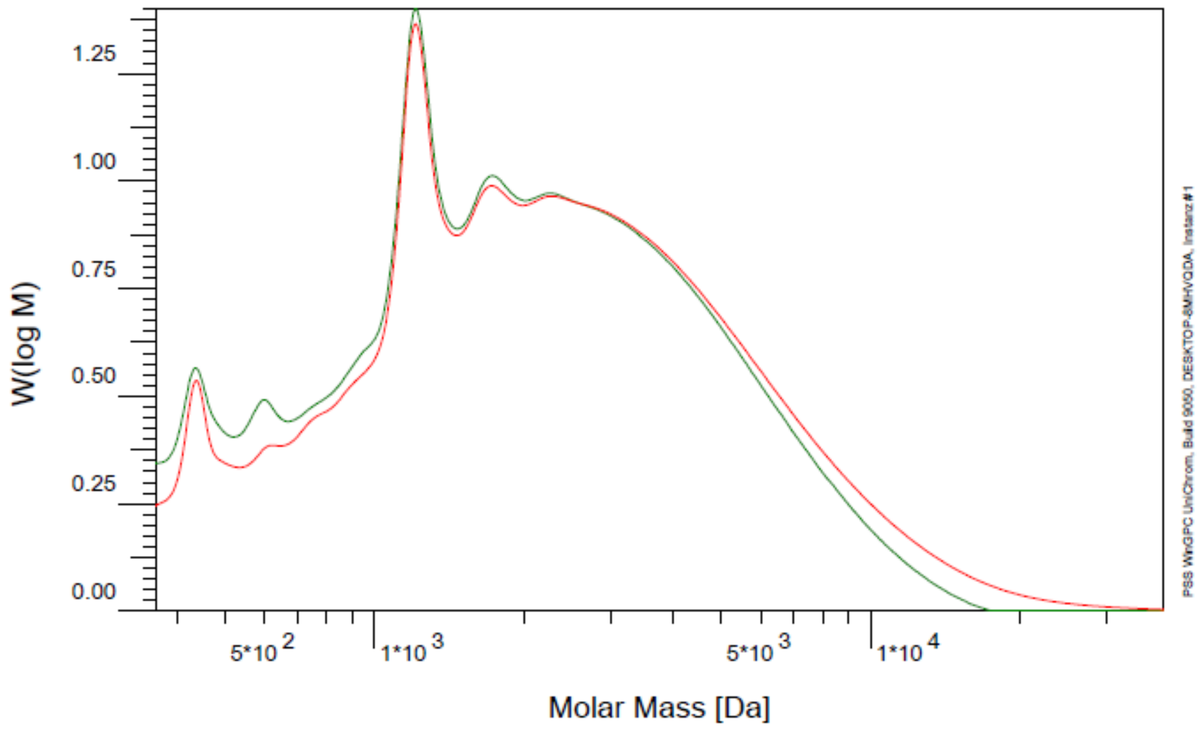

Figure S70. SEC trace of **Oligomerization product of 3**, **7b** (red line: detection by UV signal; green line: detection by RI signal).

Table S5. SEC analysis data for **Oligomerization product of 3**, **7b**.

	**RI**	**UV**
M_n	1413 g/mol	1533 g/mol
M_w	2736 g/mol	3183 g/mol
M_z	4783 g/mol	6482 g/mol
PDI	1.94	2.08

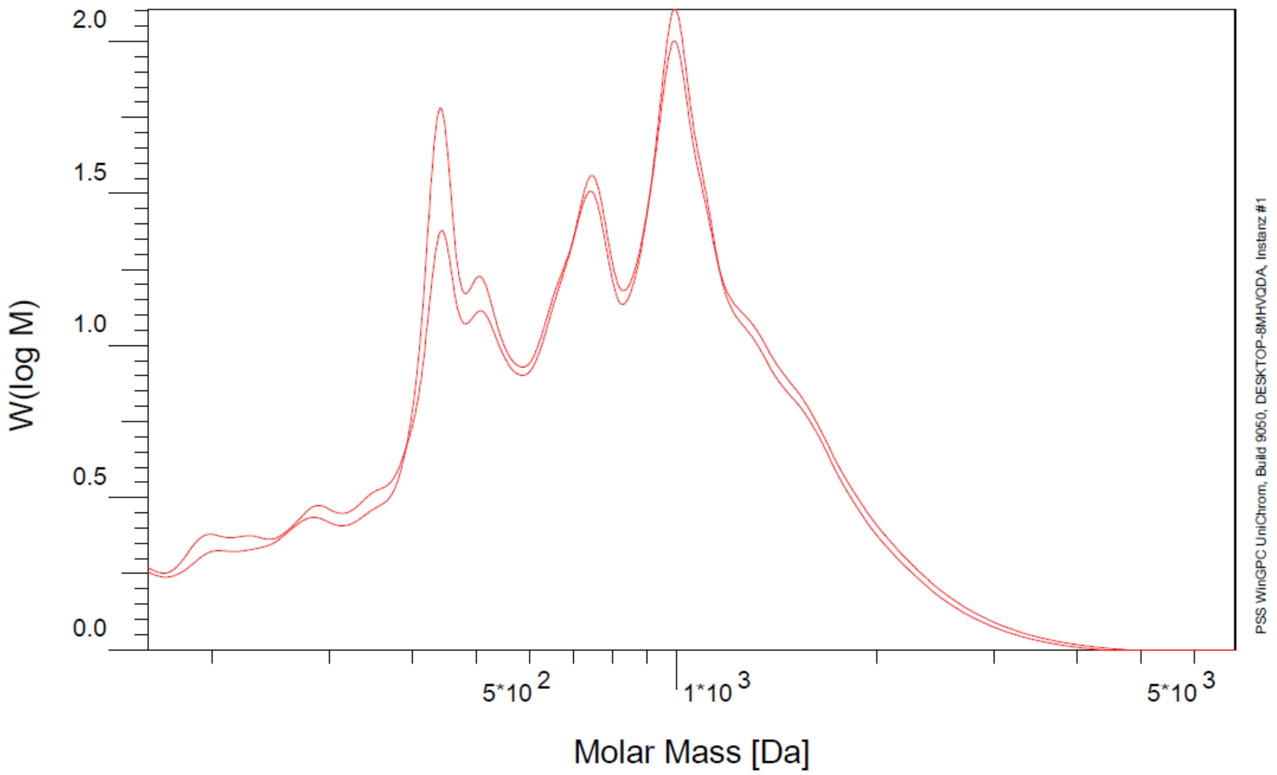

Figure S71. SEC trace of **Oligomerization product of 3**, **7c** (red line 1: detection by UV signal 1; red line 2: detection by UV signal 2).

Table S6. SEC analysis data for **Oligomerization product of 3, 7c**.

	**UV 1**	**UV 2**
M_n	602 g/mol	604 g/mol
M_w	893 g/mol	874 g/mol
M_z	1238 g/mol	1193 g/mol
PDI	1.48	1.45

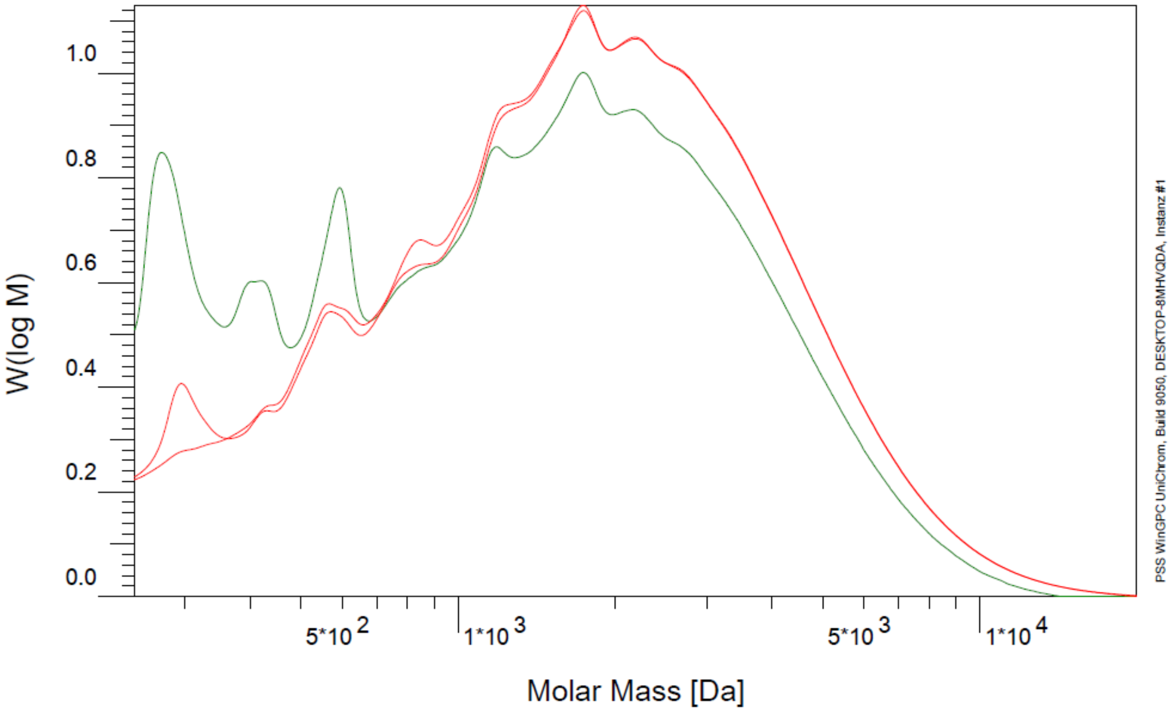

Figure S72. SEC trace of **Oligomerization product of 3**, **6c** under catalytic conditions (red lines: detection by UV signals; green line: detection by RI signal).

Table S7. SEC analysis data for **Oligomerization product of 3, 6c**.

	**RI**	**UV**
M_n	866 g/mol	1141 g/mol
M_w	1914 g/mol	2282 g/mol
M_z	3480 g/mol	3963 g/mol
PDI	2.20	2.00

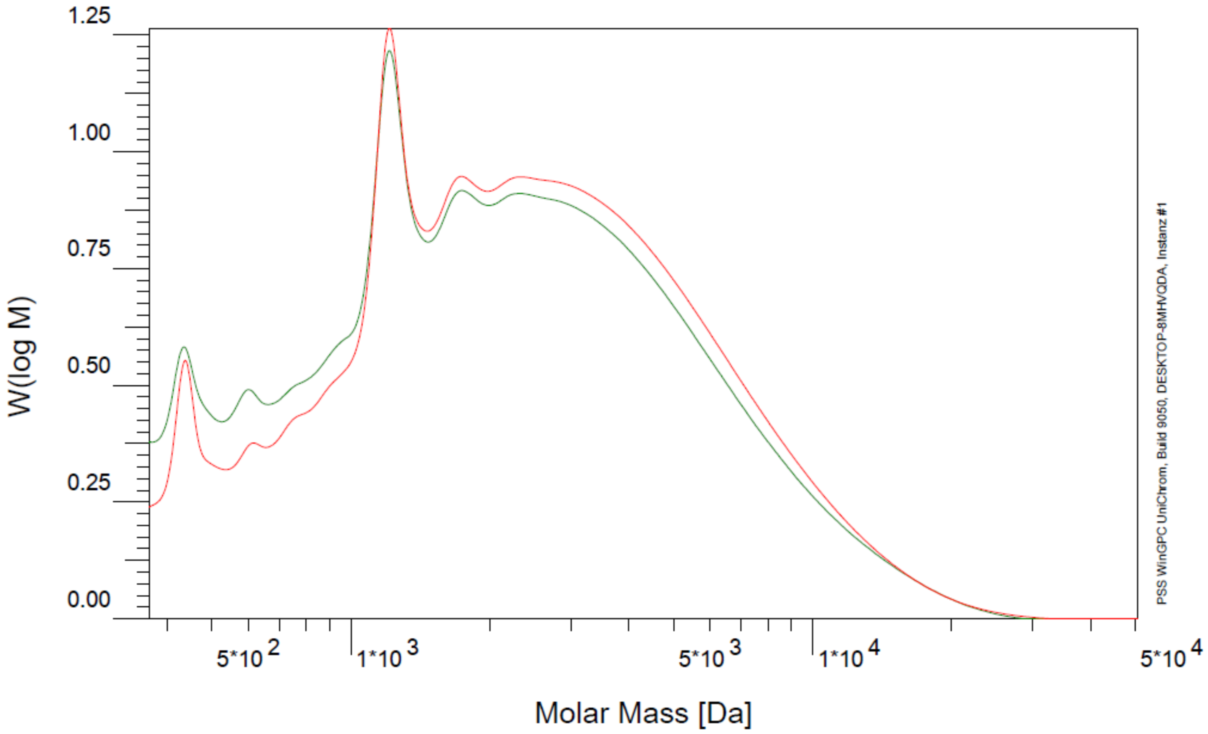

Figure S73. SEC trace of **Oligomerization product of 3**, **7b** under catalytic conditions (red line: detection by UV signal; green line: detection by RI signal).

Table S8. SEC analysis data for **Oligomerization product of 3**, **7b**.

	RI	UV
M_n	1447 g/mol	1587 g/mol
M_w	3143 g/mol	3334 g/mol
M_z	6280 g/mol	6438 g/mol
PDI	2.17	2.10

UV-vis spectra

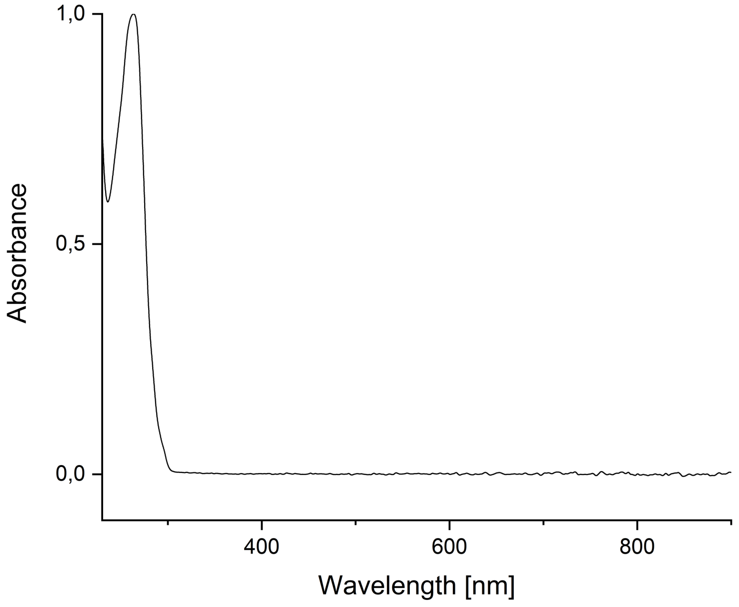

Figure S74. UV-vis absorption spectrum of **1** in dichloromethane at 25 °C. Absorption maximum: 263 nm.

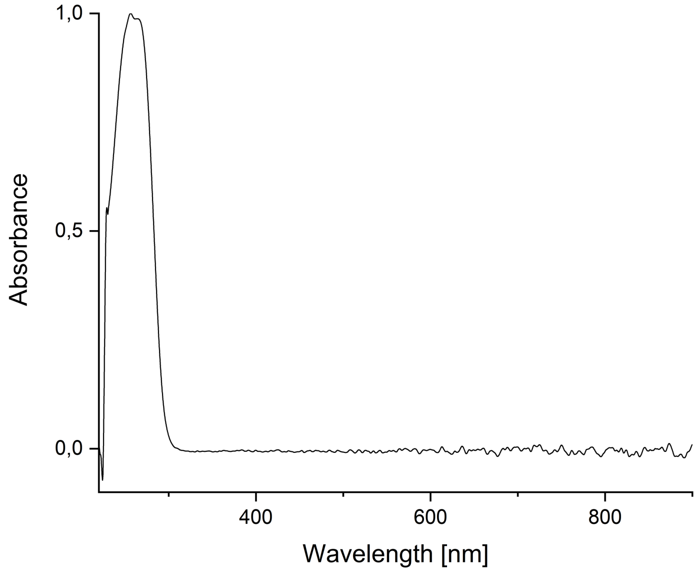

Figure S75. UV-vis absorption spectrum of **4** in dichloromethane at 25 °C. Absorption maximum: 256 nm.

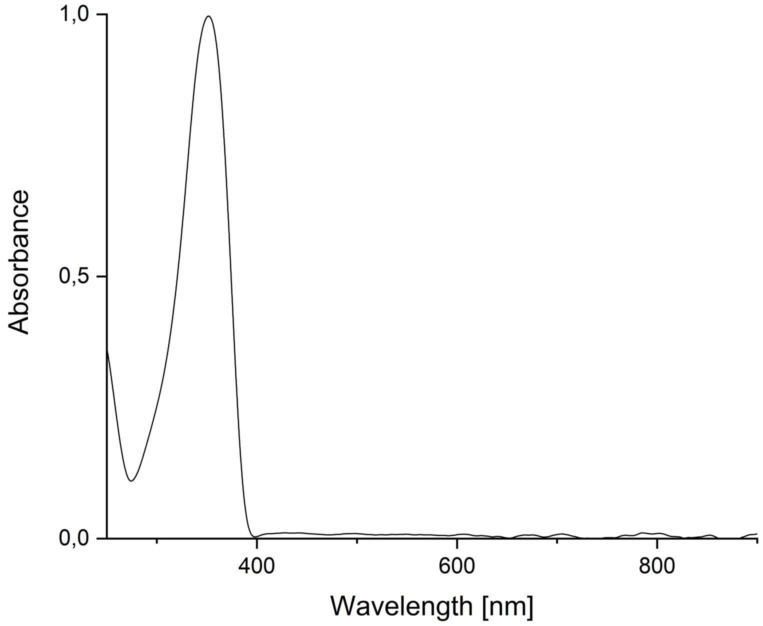

Figure S76. UV-vis absorption spectrum of **5a** in dichloromethane at 25 °C. Absorption maximum: 348 nm.

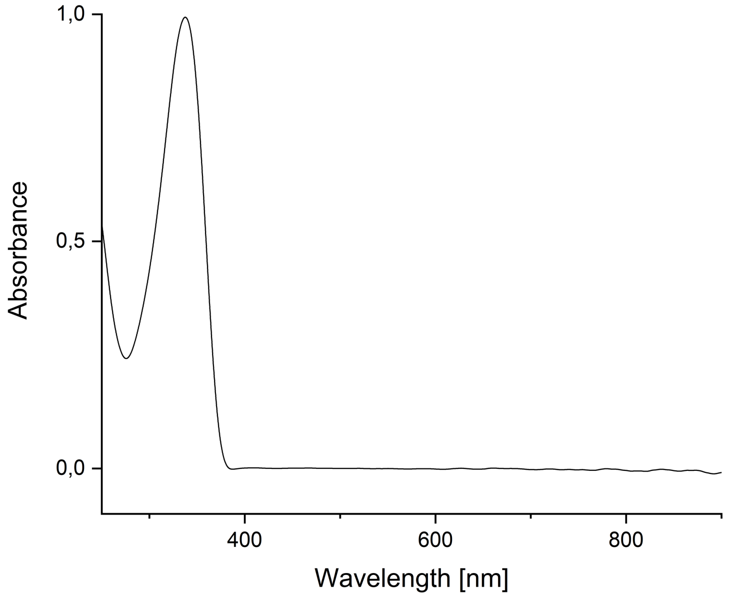

Figure S77. UV-vis absorption spectrum of **6a** in dichloromethane at 25 °C. Absorption maximum: 336 nm.

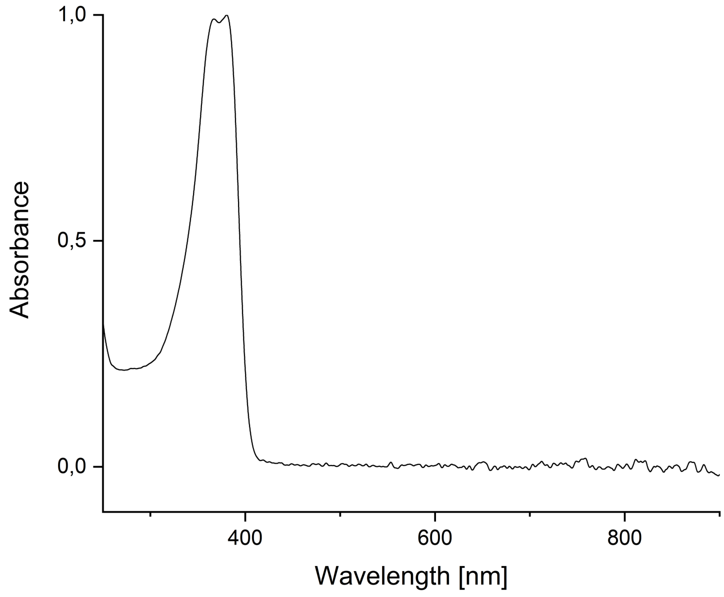

Figure S78. UV-vis absorption spectrum of **7a** in dichloromethane at 25 °C. Absorption maximum: 367 nm.

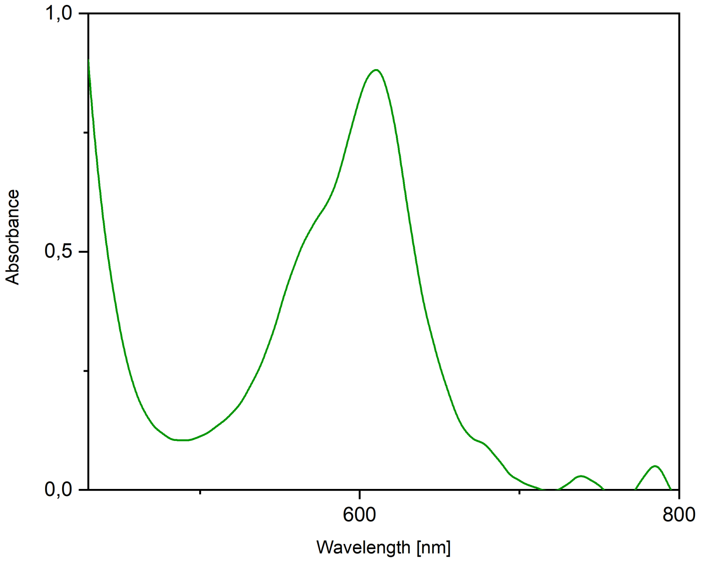

Figure S79. UV-vis absorption spectrum of **7b** in dichloromethane at 25 °C. Absorption maximum: 610 nm.

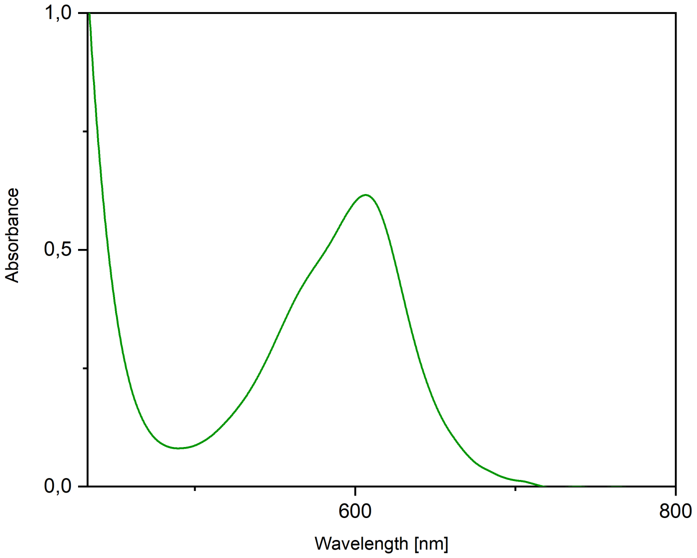

Figure S80. UV-vis absorption spectrum of **7c** in dichloromethane at 25 °C. Absorption maximum: 607 nm.

X-ray crystallographic data

The crystal data of **1**, **2**, **3, 4, 6a** and **9** were collected on a Bruker D8 Quest diffractometer with a CMOS area detector and multi-layer mirror monochromated $Mo_{K\alpha}$ radiation. The structures were solved using the intrinsic phasing method,[8] refined with the ShelXL program[9] and expanded using Fourier techniques. All non-hydrogen atoms were refined anisotropically. Hydrogen atoms were included in structure factor calculations. All hydrogen atoms were assigned to idealized geometric positions.

Special refinement details for 2: The structure was refined using TWIN keyword (matrix: TWIN -1 0 0 0 -1 0 0.209 -0.785 1). The BASF parameter was refined to 0.014%.

Special refinement details for 6a: The atoms of the residue 6 (hexane) and 16 (hexane) were only refined isotropically. The displacement parameters of atoms C1, C2, C3, C4, C5 and C6 of residue 6 and the displacement parameters of atoms C1, C2, C3, C4, C5 and C6 of residue 16 were restrained to the same value with similarity restraint SIMU. The distances between atoms C1 and C2, C2 and C3, C3 and C4, C4 and C5, C5 and C6 of residues 6 and 16 were kept during refinement at the value of 1.55 using the DFIX restraint. The distances between atoms C1 and C3, C2 and C4, C3 and C5, C4 and C6 of residues 6 and 16 were kept during refinement at the value of 2.55 using the DFIX restraint.

Crystal data for **1**: $C_{24}H_{30}B_2N_2$, $M_r = 368.12$, colorless block, 0.194×0.145×0.133 mm^3, monoclinic space group $P2_1/c$, $a = 12.192(4)$ Å, $b = 11.741(3)$ Å, $c = 16.940(3)$ Å, $\beta = 107.424(8)°$, $V = 2313.6(9)$ Å^3, $Z = 4$, $\rho_{calcd} = 1.057$ g·cm^{-3}, $\mu = 0.060$ mm^{-1}, $F(000) = 792$, $T = 100(2)$ K, $R_1 = 0.0577$, $wR^2 = 0.1110$, 4553 independent reflections [2θ≤52.042°] and 261 parameters. CCDC: 2098158.

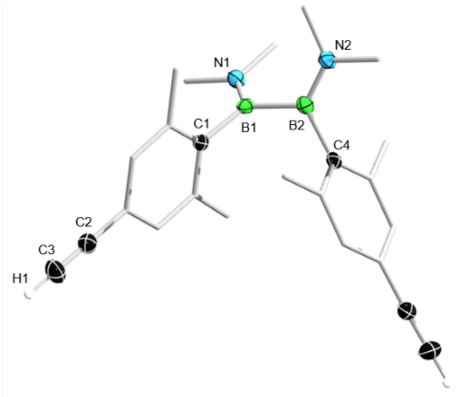

Figure S81. Crystallographically-derived molecular structure of **1**. Thermal ellipsoids drawn at the 50% probability level. Ellipsoids on methyl groups, aryl rings and most hydrogen atoms have been omitted

for clarity. Selected bond lengths (Å) and angles (°): B1–B2 1.716(2), B1–C1 1.593(2), B1–N1 1.394(2), C2–C3 1.186(3); C1-B1-B2-C4 57.4(2).

Crystal data for **2**: $C_{22}H_{26}B_2N_2$, M_r = 340.07, colorless plate, 0.553×0.405×0.32 mm^3, triclinic space group $P\bar{1}$, a = 7.0987(4) Å, b = 9.6310(5) Å, c = 16.0304(8) Å, α = 77.266(2)°, β = 88.183(2)°, γ = 70.675(2)°, V = 1007.76(9) Å^3, Z = 2, ρ_{calcd} = 1.121 g·cm^{-3}, μ = 0.064 mm^{-1}, F(000) = 364, T = 100(2) K, R_1 = 0.0590, wR^2 = 0.1573, 3924 independent reflections [2θ≤52.044°] and 242 parameters. CCDC: 2098159.

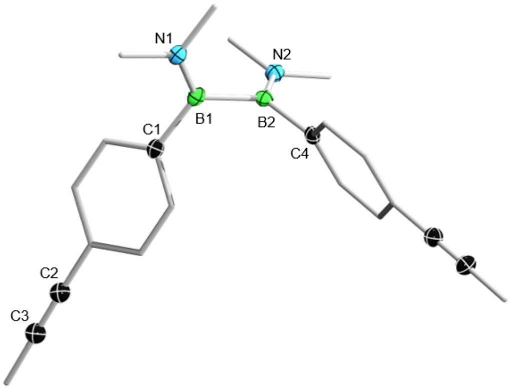

Figure S82. Crystallographically-derived molecular structure of **2**. Thermal ellipsoids drawn at the 50% probability level. Ellipsoids on methyl groups, aryl rings and hydrogen atoms have been omitted for clarity. Selected bond lengths (Å) and angles (°): B1–B2 1.713(3), B1–C1 1.591(3), B1–N1 1.397(3), C2–C3 1.194(3); C1-B1-B2-C4 86.8(2).

Crystal data for **3**: $C_3H_{3.67}B_{0.33}N_{0.33}S_{0.33}$, M_r = 58.69, colorless plate, 0.597×0.326×0.220 mm^3, monoclinic space group $P2_1/c$, a = 17.4114(15) Å, b = 6.7734(6) Å, c = 16.0154(10) Å, β = 90.890(5)°, V = 1888.5(3) Å^3, Z = 24, ρ_{calcd} = 1.238 g·cm^{-3}, μ = 0.283 mm^{-1}, F(000) = 744, T = 100(2) K, R_1 = 0.0392, wR^2 = 0.0849, 3723 independent reflections [2θ≤52.038°] and 223 parameters. CCDC: 2098160.

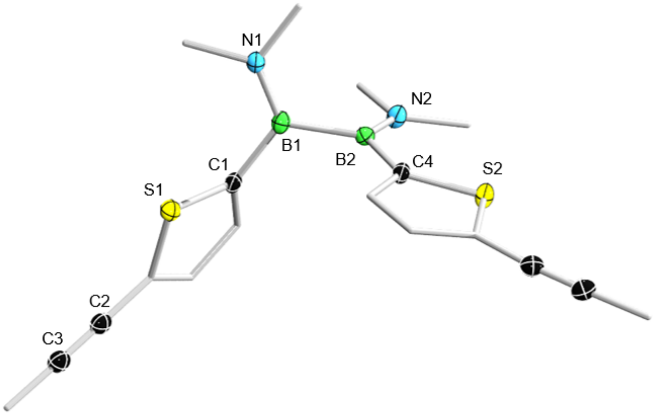

Figure S83. Crystallographically-derived molecular structure of **3**. Thermal ellipsoids drawn at the 50% probability level. Ellipsoids on methyl groups, aryl rings and hydrogen atoms have been omitted for clarity. Selected bond lengths (Å) and angles (°): B1–B2 1.723(2), B1–C1 1.584(2), B1–N1 1.393(2), C2–C3 1.193(2); C1-B1-B2-C4 81.2(2), S1-C1-B1-N1 48.5(2), S2-C4-B2-N2 1.2(2).

Crystal data for **4**: $C_{12}H_{18}B_2N_2S_2$, M_r = 276.02, colorless plate, 0.245×0.234×0.068 mm^3, orthorhombic space group *Pbcn*, a = 9.8783(3) Å, b = 11.2034(4) Å, c = 12.9833(5) Å, V = 1436.87(9) Å^3, Z = 4, ρ_{calcd} = 1.276 g·cm^{-3}, μ = 0.352 mm^{-1}, F(000) = 584, T = 100(2) K, R_1 = 0.0377, wR^2 = 0.0842, 1412 independent reflections [2θ≤52.018°] and 84 parameters. CCDC: 2098161.

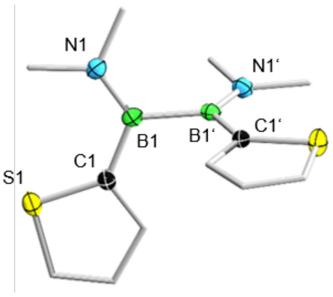

Figure S84. Crystallographically-derived molecular structure of **4**. Thermal ellipsoids drawn at the 50% probability level. Ellipsoids on methyl groups, aryl rings and hydrogen atoms have been omitted for clarity. Selected bond lengths (Å) and angles (°): B1–B1' 1.721(3), B1–C1 1.569(2), B1–N1 1.401(2); C1-B1-B1'-C1' 83.5(2), S1-C1-B1-N1 2.9(2).

Crystal data for **6a**: $C_{61}H_{79}B_4N_2$, M_r = 883.50, colorless block, 0.308×0.178×0.176 mm^3, monoclinic space group *C*2/*c*, a = 28.460(10) Å, b = 8.329(2) Å, c = 46.069(13) Å, β = 90.470(7)°, V = 10920(6) Å^3, Z = 8, ρ_{calcd} = 1.075 g·cm^{-3}, μ = 0.060 mm^{-1}, F(000) = 3832, T = 100(2) K, R_1 = 0.1131, wR^2 = 0.1629, 10762 independent reflections [2θ≤52.042°] and 644 parameters. CCDC: 2098162.

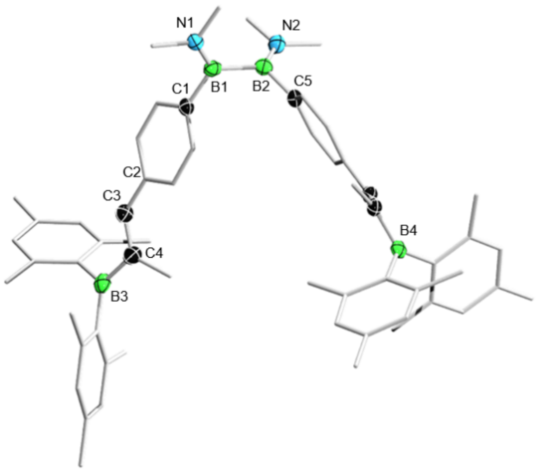

Figure S85. Crystallographically-derived molecular structure of **6a**. Thermal ellipsoids drawn at the 50% probability level. Ellipsoids on methyl groups, aryl rings and hydrogen atoms have been omitted for clarity. Selected bond lengths (Å) and angles (°): B1–B2 1.716(4), B1–C1 1.584(4), B1–N1 1.390(3), B3–C4 1.560(4), C3–C4 1.355(3); C1-B1-B2-C5 79.4(3), C2-C3-C4-B3 170.5(2).

Crystal data for **9**: $C_8H_{10}BNO_2$, M_r = 162.98, colorless block, 0.281×0.208×0.189 mm^3, triclinic space group $P\bar{1}$, a = 5.3873(5) Å, b = 7.3583(7) Å, c = 11.1673(11) Å, α = 100.569(4)°, β = 95.279(4)°, γ = 109.370(4)°, V = 404.93(7) Å^3, Z = 2, ρ_{calcd} = 1.337 g·cm^{-3}, μ = 0.094 mm^{-1}, F(000) = 172, T = 100(2) K, R_1 = 0.0652, wR^2 = 0.1548, 1595 independent reflections [2θ≤52.028°] and 111 parameters. CCDC: 2098163.

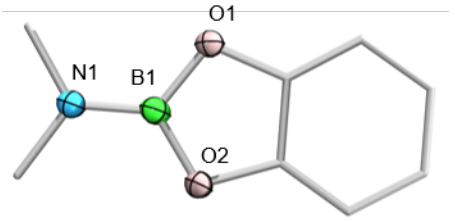

Figure S86. Crystallographically-derived molecular structure of **9**. Thermal ellipsoids drawn at the 50% probability level. Ellipsoids on methyl groups, aryl rings and hydrogen atoms have been omitted for clarity. Selected bond lengths (Å) and angles(°): B1–N1 1.375(3), B1–O1 1.409(3), B1–O2 1.412(3); C1-N1-B1-O2 0.6(3).

Computational details

Geometry optimizations and Hessian calculations were performed for **1**, **2**, **3**, **4**, **5a**, **6a**, and **7a** at the density functional theory level. The PBE0[10,11] functional was employed in conjunction with the def2-SVP[12] basis set. Dispersion corrections were considered using Grimme's D3[13] model with the Becke-Johnson (BJ)[14] damping function. All optimized structures were characterized as minimum energy structures by the analysis of the computed vibrational frequencies, as in all cases only positive eigenvalues were found. The canonical Kohn-Sham molecular orbitals of the systems and their corresponding HOMO-LUMO gaps were obtained at their corresponding optimized structures at the PBE0-D3(BJ)/def2-SVP level. NMR calculations were done for compounds **1-4** at the PBE0/6-311+G** level using the gauge-independent atomic orbital (GIAO)[15-17] method. All calculations were performed using Gaussian 16, revision C.01.[18]

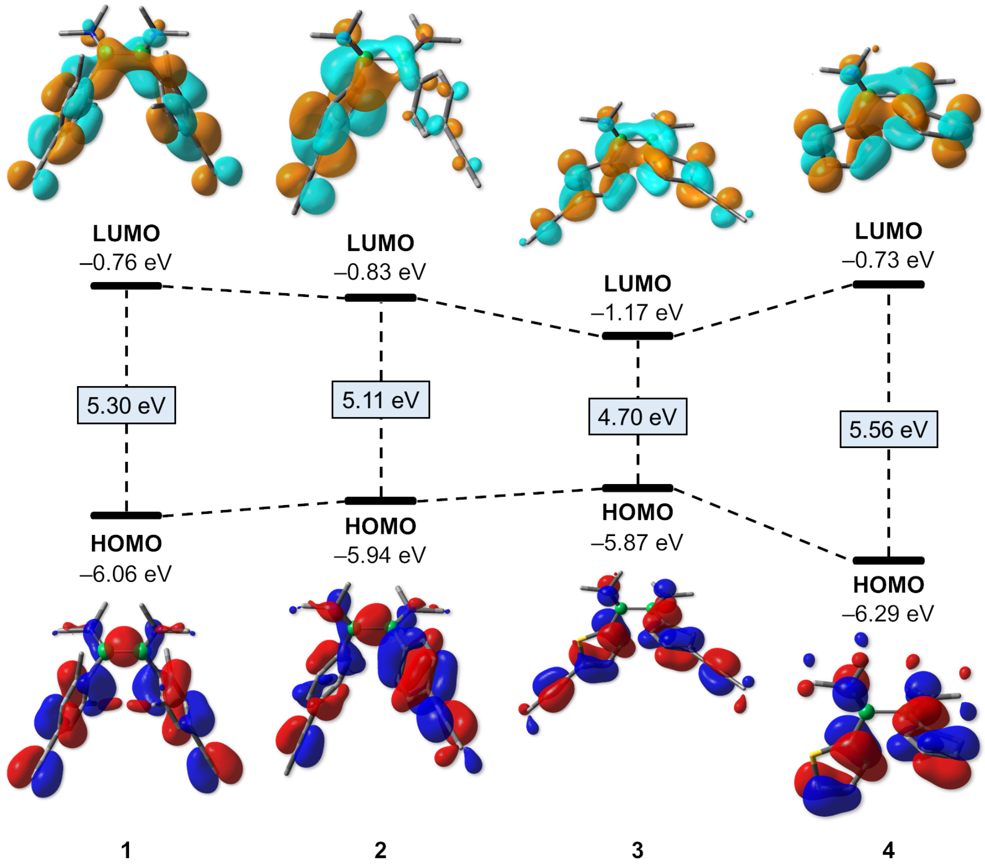

Figure S87. Canonical Kohn-Sham molecular orbitals of **1-4** at the PBE0-D3(BJ)/def2-SVP level of theory. Orbital energies are in eV. Isovalues: 0.03 a.u.

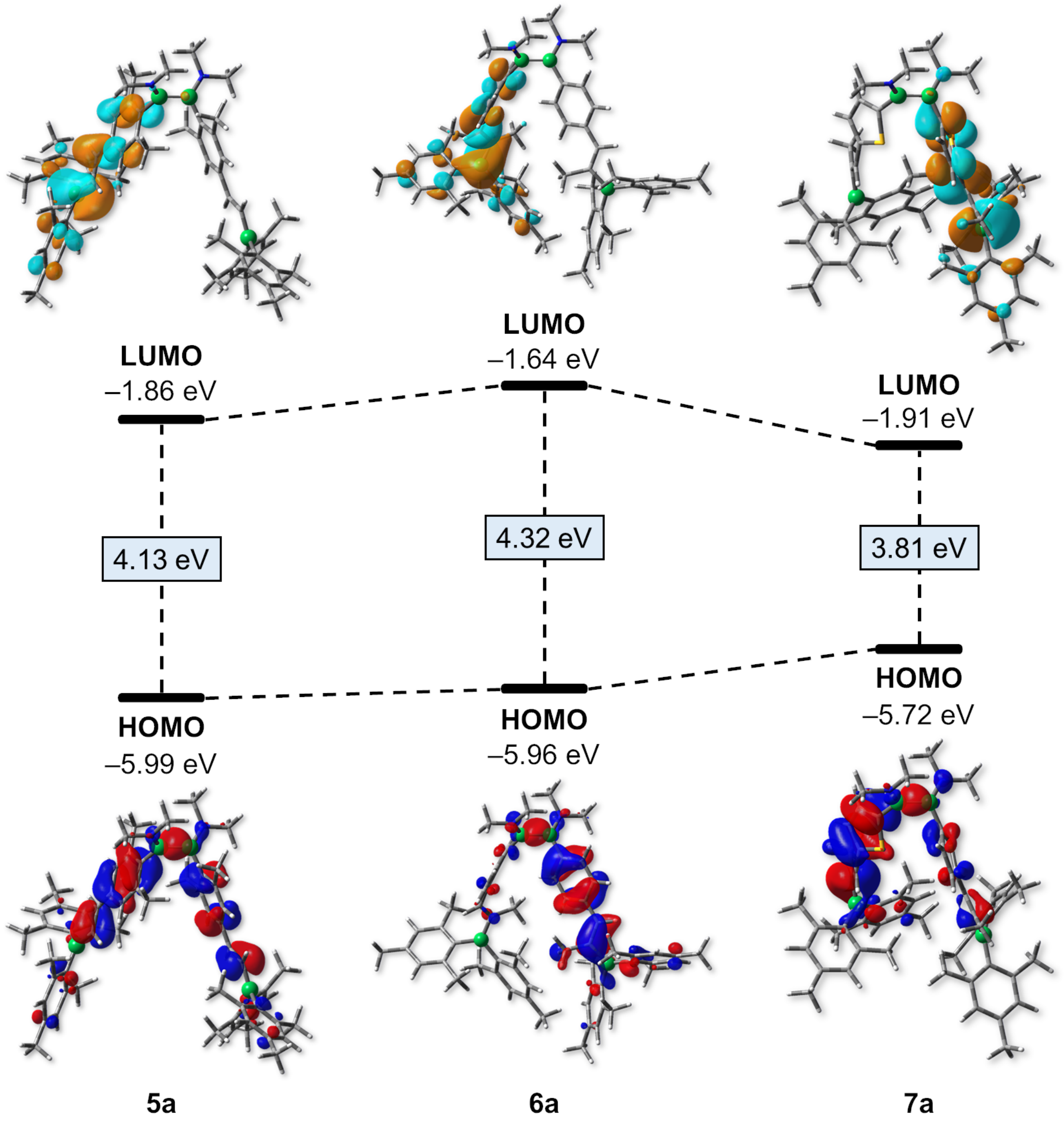

Figure S88. Canonical Kohn-Sham molecular orbitals of **5a**-**7a** at the PBE0-D3(BJ)/def2-SVP level of theory. Orbital energies are in eV. Isovalues: 0.03 a.u.

Table S9. Comparison between the experimental and calculated (PBE0/6-311+G**) ^{11}B NMR chemical shifts of compounds **1-4**.

	Exp.	*Calcd.*
**1**	49.0	50.7
**2**	48.5	49.8
**3**	43.1	42.6
**4**	44.1	43.0

Cartesian coordinates

1

B	-0.696701000	-1.478449000	0.507310000
B	0.697124000	-1.478487000	-0.507297000
N	-0.969794000	-2.458405000	1.478043000
C	-2.117493000	-2.417257000	2.356946000
H	-1.805877000	-2.374827000	3.416384000
H	-2.741115000	-3.321318000	2.235742000
H	-2.732906000	-1.535943000	2.137190000
C	-0.128978000	-3.607467000	1.716012000
H	0.732034000	-3.596815000	1.037142000
H	-0.686856000	-4.549364000	1.562849000
H	0.248484000	-3.617648000	2.754555000
N	0.970359000	-2.458466000	-1.477948000
C	0.129729000	-3.607675000	-1.715845000
H	-0.731439000	-3.596957000	-1.037177000
H	0.687662000	-4.549481000	-1.562322000
H	-0.247471000	-3.618129000	-2.754476000
C	2.118150000	-2.417247000	-2.356725000
H	1.806651000	-2.375076000	-3.416207000
H	2.741952000	-3.321155000	-2.235287000
H	2.733359000	-1.535766000	-2.137050000
C	1.673810000	-0.239271000	-0.351033000
C	2.665987000	-0.237274000	0.652027000
C	3.467700000	0.886846000	0.843397000
H	4.236293000	0.884891000	1.619796000
C	3.299065000	2.033255000	0.053740000
C	2.314524000	2.027025000	-0.944185000
H	2.182892000	2.917045000	-1.563752000
C	1.507488000	0.909077000	-1.155372000
C	2.842985000	-1.440353000	1.535556000
H	3.690144000	-1.317416000	2.224623000
H	3.016151000	-2.354088000	0.944943000
H	1.938781000	-1.616355000	2.141180000
C	4.117705000	3.185685000	0.261457000
C	4.813119000	4.164724000	0.438376000

H	5.427235000	5.031810000	0.594355000
C	0.460272000	0.940575000	-2.233224000
H	-0.550740000	0.887088000	-1.799947000
H	0.563973000	0.085674000	-2.919638000
H	0.522163000	1.862471000	-2.827541000
C	-1.673667000	-0.239446000	0.351028000
C	-1.507595000	0.909019000	1.155239000
C	-2.314988000	2.026717000	0.944050000
H	-2.183551000	2.916830000	1.563526000
C	-3.299629000	2.032574000	-0.053769000
C	-3.467975000	0.886063000	-0.843346000
H	-4.236614000	0.883846000	-1.619699000
C	-2.665921000	-0.237809000	-0.651966000
C	-0.460309000	0.940923000	2.233008000
H	0.550692000	0.887231000	1.799741000
H	-0.564027000	0.086297000	2.919767000
H	-0.522149000	1.863051000	2.826971000
C	-4.118646000	3.184741000	-0.261468000
C	-4.814258000	4.163594000	-0.438633000
H	-5.428389000	5.030539000	-0.595335000
C	-2.842655000	-1.440987000	-1.535408000
H	-3.689895000	-1.318306000	-2.224421000
H	-3.015553000	-2.354719000	-0.944714000
H	-1.938457000	-1.616820000	-2.141095000

2

N	0.554495888	-2.640522055	1.731816835
C	1.757534685	-0.733762169	0.426911891
B	0.593677796	-1.784335867	0.617241641
C	2.458699879	-0.666322307	-0.789728685
H	2.220211070	-1.378993556	-1.584965274
N	-0.704170355	-2.681483438	-1.621958585
B	-0.653583963	-1.758540804	-0.560523016
C	3.447583914	0.283275839	-1.016013211
H	3.987748825	0.305902564	-1.964817552
C	3.754588159	1.239923541	-0.032494948
C	2.072400742	0.232857046	1.398682563

H	1.531865317	0.236023097	2.349724509
C	3.041957535	1.204306409	1.178826260
H	3.261761645	1.950280984	1.945558950
C	5.613105796	3.071419145	-0.456359665
C	4.758701079	2.229410695	-0.261766665
C	6.633705138	4.078438687	-0.687959655
H	7.516296800	3.905753329	-0.051347744
H	6.254486879	5.088456521	-0.465107550
H	6.970152964	4.069935984	-1.736472049
C	1.574173509	-2.733406468	2.751571165
H	1.923119940	-3.776408640	2.857492252
H	1.189667848	-2.416720915	3.738323584
H	2.436316560	-2.106405142	2.496027069
C	-0.547002399	-3.544761569	1.964546904
H	-1.045515171	-3.329150204	2.926830055
H	-0.204153332	-4.595276184	1.999693084
H	-1.291888805	-3.449378608	1.163514982
C	-1.741764028	-0.625436411	-0.416660753
C	-3.111426136	-0.921212197	-0.295300835
H	-3.445601457	-1.962013531	-0.352266646
C	-4.062174700	0.072643798	-0.095211636
H	-5.119392842	-0.183408534	0.002464459
C	-3.676274551	1.422232121	-0.020854456
C	-2.310303333	1.733171587	-0.139325218
H	-1.997001625	2.777659873	-0.080108504
C	-1.368462907	0.727028785	-0.318005985
H	-0.311476344	0.998456306	-0.387603335
C	-4.647400207	2.451311432	0.175034362
C	-5.472536634	3.327944389	0.341380912
C	-6.456669577	4.377498071	0.540413063
H	-6.296109686	4.898734439	1.497804458
H	-7.479014887	3.968264730	0.549262669
H	-6.403959184	5.130949454	-0.261704821
C	-1.634488031	-2.626052004	-2.727013943
H	-2.272326266	-1.737810175	-2.650950006
H	-2.280913141	-3.522307007	-2.755974888
H	-1.092996378	-2.583486061	-3.689190995

C	0.226362970	-3.779780959	-1.736142663
H	0.907241553	-3.790991775	-0.874569123
H	0.829427060	-3.704047842	-2.659661845
H	-0.302560759	-4.749962087	-1.771729388

3

S	3.220793000	0.104928000	0.768727000
N	0.893892000	-2.341084000	1.558049000
B	0.740024000	-1.494341000	0.440659000
C	1.822406000	-0.484418000	-0.073170000
N	-0.893903000	-2.341103000	-1.558033000
S	-3.220793000	0.104929000	-0.768726000
B	-0.740024000	-1.494345000	-0.440657000
C	1.736919000	0.161316000	-1.298524000
H	0.918002000	-0.036008000	-1.993495000
C	2.772958000	1.082838000	-1.559394000
H	2.868213000	1.675717000	-2.469228000
C	3.674803000	1.176961000	-0.513518000
C	4.819929000	1.997131000	-0.406856000
C	-1.736897000	0.161350000	1.298506000
H	-0.917973000	-0.035963000	1.993473000
C	-1.822396000	-0.484405000	0.073164000
C	6.986226000	3.537067000	-0.180280000
H	6.724671000	4.604936000	-0.252027000
H	7.717633000	3.316402000	-0.974180000
H	7.484708000	3.378130000	0.788717000
C	5.808656000	2.697668000	-0.301084000
C	-2.772941000	1.082867000	1.559378000
H	-2.868192000	1.675752000	2.469208000
C	-3.674797000	1.176974000	0.513509000
C	-4.819931000	1.997136000	0.406856000
C	-5.808662000	2.697666000	0.301080000
C	-6.986238000	3.537054000	0.180273000
H	-7.719902000	3.312841000	0.971096000
H	-7.481790000	3.381902000	-0.790837000
H	-6.725328000	4.604718000	0.257177000
C	-0.186913000	-3.183589000	2.017464000

H	-0.473363000	-2.936885000	3.056279000
H	0.102890000	-4.250246000	2.000980000
H	-1.072612000	-3.053003000	1.382641000
C	2.073229000	-2.475714000	2.380259000
H	1.949945000	-1.983501000	3.362535000
H	2.955559000	-2.048702000	1.889171000
H	2.285803000	-3.541967000	2.572519000
C	0.186898000	-3.183619000	-2.017440000
H	1.072581000	-3.053069000	-1.382587000
H	0.473384000	-2.936894000	-3.056241000
H	-0.102929000	-4.250270000	-2.000993000
C	-2.073237000	-2.475727000	-2.380247000
H	-2.955558000	-2.048676000	-1.889179000
H	-2.285839000	-3.541981000	-2.572478000
H	-1.949930000	-1.983546000	-3.362536000

4

S	-3.287845000	-0.924353000	0.377253000
N	-1.077492000	1.538334000	1.435147000
B	-0.788127000	0.688182000	0.347363000
C	-1.799537000	-0.322678000	-0.293055000
N	1.077492000	1.538339000	-1.435142000
S	3.287847000	-0.924351000	-0.377254000
B	0.788128000	0.688183000	-0.347360000
C	-1.573927000	-0.963371000	-1.500663000
H	-0.681319000	-0.762638000	-2.097206000
C	-2.578649000	-1.890021000	-1.878003000
H	-2.562445000	-2.478037000	-2.796766000
C	-3.576909000	-1.977030000	-0.940080000
C	1.573926000	-0.963376000	1.500659000
H	0.681318000	-0.762646000	2.097202000
C	1.799538000	-0.322678000	0.293054000
C	2.578648000	-1.890028000	1.877997000
H	2.562444000	-2.478047000	2.796757000
C	3.576911000	-1.977031000	0.940075000
C	-0.061988000	2.383893000	2.020248000
H	0.097193000	2.140175000	3.086777000

H	-0.349404000	3.450043000	1.966352000
H	0.894309000	2.253742000	1.497670000
C	-2.348584000	1.673233000	2.106463000
H	-2.345548000	1.185759000	3.098898000
H	-3.162661000	1.240963000	1.512899000
H	-2.585588000	2.739742000	2.266633000
C	0.061986000	2.383898000	-2.020240000
H	-0.894305000	2.253759000	-1.497649000
H	-0.097211000	2.140168000	-3.086765000
H	0.349410000	3.450046000	-1.966361000
C	2.348582000	1.673241000	-2.106460000
H	3.162658000	1.240958000	-1.512905000
H	2.585591000	2.739750000	-2.266617000
H	2.345540000	1.185780000	-3.098902000
H	-4.461329000	-2.613553000	-0.957636000
H	4.461331000	-2.613554000	0.957630000

5a

B	-0.431008615	5.266138213	0.375825672
N	-0.488945942	6.318622103	1.305779068
C	-1.359754253	3.994857660	0.560354229
B	0.666559706	5.196361922	-0.952056711
N	0.672227274	6.083148017	-2.041460187
C	-2.593706008	3.913097287	-0.128919872
B	-5.569646162	-1.266635091	0.364933752
C	-3.360768073	2.755848806	-0.057500668
H	-4.308857458	2.711926745	-0.599132550
B	4.984645709	-1.877808588	-0.463423882
C	-2.935108526	1.643466687	0.687018117
C	-1.716186204	1.738479224	1.368874438
H	-1.375103685	0.880735937	1.957117171
C	-0.927638240	2.890451586	1.320879474
C	-3.703374538	0.407006731	0.782316970
H	-3.222356964	-0.385635744	1.369928617
C	-4.908025368	0.126721519	0.229383945
C	-1.368012778	6.322738943	2.454236285
H	-1.983032669	5.414311526	2.464038886

H	-2.036295089	7.202499453	2.437438781
H	-0.791676761	6.366633244	3.396047321
C	0.335062436	7.501585831	1.229746172
H	0.997089092	7.449053929	0.357076320
H	0.961103721	7.610736153	2.133760283
H	-0.282701233	8.414682338	1.149276701
C	1.669772993	3.971401645	-0.912678722
C	2.861966768	4.047691558	-0.165248342
C	3.667627996	2.918318483	-0.026279236
H	4.590050511	2.982485336	0.559137376
C	3.315722649	1.688277247	-0.600062298
C	2.138222321	1.630665884	-1.361004069
H	1.850937152	0.689948568	-1.836842765
C	1.321570278	2.745763588	-1.528100168
C	4.172516750	0.530227676	-0.370543261
H	5.088031925	0.742702031	0.196703262
C	3.967344401	-0.749858497	-0.763629134
C	1.576599263	5.963501200	-3.163297365
H	2.248397675	5.108081504	-3.018315954
H	2.188618294	6.876301527	-3.277829737
H	1.021907484	5.821057109	-4.108475523
C	-0.233454088	7.199858171	-2.169962743
H	-0.904198921	7.247210539	-1.303657893
H	-0.850773186	7.109953867	-3.082086242
H	0.317293531	8.155518627	-2.242075087
C	-5.026976132	-2.247938265	1.478598414
C	-5.082379323	-1.915452723	2.847679003
C	-4.590704881	-2.811396119	3.803676170
H	-4.662335811	-2.547820461	4.863934965
C	-4.016505999	-4.029644788	3.445421754
C	-3.955908388	-4.346562433	2.084829178
H	-3.508679105	-5.297361237	1.777249453
C	-4.456927719	-3.488512906	1.106003745
C	-5.680353853	-0.619333584	3.331508736
H	-6.157282098	-0.750884567	4.314193761
H	-6.431833437	-0.215629599	2.638652530
H	-4.909198548	0.160646365	3.440341790

C	-3.468770086	-4.972006760	4.477572262
H	-3.848937769	-5.994840346	4.329567784
H	-3.734060881	-4.654361893	5.495924628
H	-2.369079749	-5.026898554	4.420500697
C	-4.373272988	-3.899292858	-0.339441406
H	-3.879449954	-3.129389216	-0.953268871
H	-5.374022669	-4.052212800	-0.771944858
H	-3.803734972	-4.832192511	-0.454521615
C	-6.725535065	-1.697143755	-0.617710577
C	-7.885870909	-2.363661540	-0.143063448
C	-8.895131623	-2.731216293	-1.034392955
H	-9.788184484	-3.229140228	-0.643675008
C	-8.794200694	-2.497760193	-2.407234354
C	-7.639721942	-1.871334972	-2.873293865
H	-7.524868201	-1.699637661	-3.948305249
C	-6.620208631	-1.457555443	-2.010826264
C	-8.084195959	-2.695882520	1.311859770
H	-7.378941965	-3.471087269	1.647182792
H	-9.104578285	-3.063957195	1.489049654
H	-7.924530853	-1.825933993	1.964621010
C	-9.899848503	-2.893570440	-3.341043558
H	-10.341709019	-3.860129402	-3.055786593
H	-9.544974620	-2.970168413	-4.378807504
H	-10.713416613	-2.148852757	-3.324629660
C	-5.418825037	-0.795301255	-2.635899861
H	-5.333394186	-1.074580443	-3.696080872
H	-4.477417124	-1.061015810	-2.136863982
H	-5.497572697	0.303211536	-2.596475314
C	4.928672530	-3.234663055	-1.264276993
C	4.758618585	-3.239396271	-2.671569508
C	4.738199270	-4.448905651	-3.372174912
H	4.631728601	-4.427186455	-4.461426900
C	4.851376017	-5.679268775	-2.727206813
C	5.017368972	-5.671033930	-1.340946394
H	5.116027603	-6.623887260	-0.811384412
C	5.075823018	-4.484897033	-0.607591782
C	4.630232427	-1.974339562	-3.481133773

H	4.924629865	-2.153652527	-4.525370901
H	5.245586090	-1.153208853	-3.089918404
H	3.590393978	-1.609743099	-3.496731150
C	4.779178682	-6.970074487	-3.488516901
H	5.484259286	-7.714112992	-3.088422208
H	5.001381797	-6.823686756	-4.555268368
H	3.770300834	-7.410657251	-3.419226739
C	5.290725322	-4.593120237	0.878589367
H	6.280073922	-4.210725014	1.171420647
H	5.220451277	-5.640562052	1.204104989
H	4.551108606	-4.015108336	1.450634688
C	6.088045605	-1.615670239	0.637545918
C	7.457436626	-1.640414292	0.281012814
C	8.432851533	-1.388170648	1.245416639
H	9.486808409	-1.401417431	0.949177098
C	8.102163696	-1.128707110	2.579214711
C	6.751904021	-1.118233010	2.923503700
H	6.469432063	-0.931635454	3.964730519
C	5.744978354	-1.344079467	1.977990742
C	7.883755523	-1.931070304	-1.132822585
H	7.633698900	-2.963242006	-1.422814766
H	8.967076604	-1.793116764	-1.257100910
H	7.377777511	-1.270826048	-1.854871056
C	9.173513147	-0.866282657	3.597523911
H	9.931134045	-1.665721469	3.597549561
H	8.757161144	-0.793476324	4.612215889
H	9.702631763	0.076924787	3.383857622
C	4.312603044	-1.309931702	2.446171507
H	4.232432114	-1.661612678	3.485556319
H	3.649452890	-1.927690682	1.824742688
H	3.904022718	-0.286690934	2.411470513
C	-3.058016223	5.072500949	-0.965262541
H	-3.094415294	6.005342345	-0.380254090
H	-2.367241614	5.249091953	-1.806225670
H	-4.058465601	4.896033021	-1.384454151
C	0.381338594	2.911165488	2.061003735
H	0.713765360	3.937297912	2.273635303

H	0.306506129	2.371154473	3.016589534
H	1.174917164	2.428711775	1.467901990
C	0.069160522	2.637856213	-2.351930156
H	-0.825922816	2.800565311	-1.731579552
H	0.049238228	3.391908313	-3.154276136
H	-0.021639229	1.646201338	-2.816198847
C	3.246789210	5.334203298	0.509479214
H	2.519293098	5.597642973	1.294990282
H	4.237249458	5.265226837	0.980688413
H	3.267250558	6.175086902	-0.201862360
H	-5.393749232	0.906656055	-0.368668062
H	3.059139566	-0.972534151	-1.335884718

6a

B	-1.191569000	5.918087000	0.413040000
N	-1.268712000	6.958528000	1.353957000
C	-2.218905000	4.718898000	0.397437000
B	0.090584000	5.729003000	-0.719596000
N	0.248914000	6.575179000	-1.833662000
C	-2.795285000	4.275995000	-0.805263000
H	-2.689823000	4.887418000	-1.706992000
B	-4.349062000	-1.537626000	-0.449227000
C	-3.446408000	3.050645000	-0.898882000
H	-3.876679000	2.735782000	-1.851406000
B	4.447461000	-1.240358000	0.438894000
C	-3.545675000	2.203882000	0.218280000
C	-3.043677000	2.673184000	1.442550000
H	-3.131899000	2.040194000	2.330150000
C	-2.386311000	3.895524000	1.524472000
H	-1.950696000	4.196496000	2.481702000
C	-4.107175000	0.850598000	0.146275000
H	-4.717866000	0.549683000	1.007153000
C	-3.851848000	-0.096032000	-0.794022000
C	-2.979364000	0.135845000	-1.994989000
H	-3.588507000	0.423478000	-2.869585000
H	-2.455330000	-0.790014000	-2.276165000
H	-2.238534000	0.936518000	-1.843235000

C	-2.346414000	7.136302000	2.300219000
H	-3.137904000	6.398018000	2.125374000
H	-2.785499000	8.145685000	2.204397000
H	-1.991831000	7.032786000	3.342021000
C	-0.247059000	7.971781000	1.473135000
H	0.544436000	7.799430000	0.732172000
H	0.211509000	7.959879000	2.478412000
H	-0.663103000	8.983829000	1.314516000
C	1.051676000	4.489654000	-0.516070000
C	2.437771000	4.626823000	-0.317141000
H	2.887883000	5.624666000	-0.321219000
C	3.261103000	3.522609000	-0.124381000
H	4.337443000	3.662164000	0.013329000
C	2.734156000	2.219364000	-0.097082000
C	1.352582000	2.073907000	-0.305551000
H	0.911702000	1.077069000	-0.343677000
C	0.536799000	3.180620000	-0.502060000
H	-0.530758000	3.018089000	-0.661929000
C	3.630873000	1.078169000	0.086708000
H	4.647250000	1.228689000	-0.299904000
C	3.372340000	-0.130083000	0.655227000
C	2.089237000	-0.472624000	1.359216000
H	1.583781000	0.413304000	1.770357000
H	2.280318000	-1.180320000	2.180368000
H	1.377430000	-0.979715000	0.688525000
C	1.211741000	6.375862000	-2.894101000
H	1.788105000	5.458978000	-2.725236000
H	1.914750000	7.225966000	-2.962142000
H	0.703575000	6.293692000	-3.871705000
C	-0.594039000	7.725124000	-2.064482000
H	-1.322970000	7.831213000	-1.250756000
H	-1.145893000	7.631077000	-3.017474000
H	0.002142000	8.654296000	-2.124099000
C	-5.744994000	-1.731431000	0.253808000
C	-6.909739000	-1.148795000	-0.300323000
C	-8.148971000	-1.348523000	0.312739000
H	-9.042706000	-0.912044000	-0.144314000

C	-8.280133000	-2.088692000	1.488197000
C	-7.126389000	-2.656586000	2.032033000
H	-7.205007000	-3.246834000	2.950454000
C	-5.874464000	-2.506721000	1.431842000
C	-6.873970000	-0.333350000	-1.567585000
H	-7.868245000	-0.298577000	-2.035739000
H	-6.168718000	-0.736513000	-2.308596000
H	-6.558870000	0.701940000	-1.366630000
C	-9.612066000	-2.251079000	2.159855000
H	-9.696511000	-3.228003000	2.658388000
H	-10.440528000	-2.157062000	1.442951000
H	-9.758439000	-1.477865000	2.932951000
C	-4.691143000	-3.181631000	2.071536000
H	-3.852010000	-2.487300000	2.226331000
H	-4.307771000	-3.999923000	1.443267000
H	-4.961820000	-3.600954000	3.050666000
C	-3.420897000	-2.770191000	-0.777808000
C	-3.913929000	-3.864624000	-1.525011000
C	-3.085168000	-4.958267000	-1.788973000
H	-3.480626000	-5.790766000	-2.379447000
C	-1.776987000	-5.027137000	-1.308575000
C	-1.301966000	-3.951384000	-0.555253000
H	-0.286932000	-3.991412000	-0.146875000
C	-2.089513000	-2.825984000	-0.299590000
C	-5.324232000	-3.887455000	-2.050764000
H	-6.056199000	-3.961783000	-1.231940000
H	-5.478940000	-4.741063000	-2.725513000
H	-5.571739000	-2.972572000	-2.610548000
C	-0.901045000	-6.208860000	-1.606020000
H	-1.494876000	-7.096836000	-1.866236000
H	-0.260889000	-6.461163000	-0.748251000
H	-0.232408000	-6.000597000	-2.458667000
C	-1.485257000	-1.714509000	0.516625000
H	-0.586023000	-2.065330000	1.041187000
H	-2.179436000	-1.311993000	1.267650000
H	-1.189235000	-0.862422000	-0.116474000
C	3.970317000	-2.746363000	0.363483000

C	2.990657000	-3.169346000	-0.563546000
C	2.589802000	-4.509748000	-0.597112000
H	1.849873000	-4.819246000	-1.341661000
C	3.109692000	-5.458590000	0.280778000
C	4.075015000	-5.033683000	1.196979000
H	4.502298000	-5.759791000	1.895749000
C	4.520695000	-3.712184000	1.243230000
C	2.392551000	-2.249531000	-1.595395000
H	2.759247000	-2.512373000	-2.601527000
H	2.642138000	-1.194987000	-1.422422000
H	1.295471000	-2.346234000	-1.623663000
C	2.632244000	-6.881416000	0.262427000
H	3.411393000	-7.573407000	0.613992000
H	2.326508000	-7.192385000	-0.747131000
H	1.758654000	-7.012417000	0.923661000
C	5.581241000	-3.349672000	2.247652000
H	6.510160000	-3.027933000	1.753161000
H	5.816427000	-4.204927000	2.896406000
H	5.266995000	-2.518527000	2.897587000
C	5.969666000	-0.855702000	0.293398000
C	6.720604000	-1.285308000	-0.828354000
C	8.066689000	-0.937700000	-0.940329000
H	8.626638000	-1.266917000	-1.821526000
C	8.721311000	-0.191420000	0.043659000
C	7.980051000	0.213925000	1.152112000
H	8.475154000	0.787049000	1.942506000
C	6.621278000	-0.090594000	1.286818000
C	6.091515000	-2.102743000	-1.923812000
H	5.797150000	-3.099470000	-1.561438000
H	6.786323000	-2.235243000	-2.764935000
H	5.179532000	-1.626361000	-2.315296000
C	10.172910000	0.161457000	-0.100471000
H	10.784871000	-0.733011000	-0.296460000
H	10.562446000	0.648352000	0.804656000
H	10.331197000	0.850091000	-0.946546000
C	5.909566000	0.406685000	2.518865000
H	6.618267000	0.543754000	3.348699000

H	5.118647000	-0.276109000	2.858300000
H	5.420676000	1.375958000	2.333064000

7a

S	2.217104514	-2.568238633	-0.549784337
N	5.519396443	-3.373289910	-1.232932729
B	5.060255861	-2.673440991	-0.105552732
C	3.596902361	-2.843143272	0.445216916
N	7.130203152	-1.579880897	1.394894935
S	3.743276762	0.369311751	0.962128965
B	5.955766798	-1.415526355	0.646806821
C	3.190360706	-2.979063750	1.762924673
H	3.901251687	-3.122012898	2.579581997
C	1.793505048	-2.902239226	1.953399653
H	1.314388290	-2.986280013	2.926101003
C	1.096999085	-2.659738327	0.776184690
C	-0.303795749	-2.490569240	0.482203707
C	5.778846757	1.034340524	-0.416824771
H	6.761008515	1.021593131	-0.894727738
C	5.336112305	0.010359710	0.402801140
C	4.831539321	2.070568942	-0.611145006
H	5.013596594	2.957212233	-1.218243001
C	3.655656752	1.863652287	0.088368171
C	2.419366992	2.618417432	0.145548920
C	6.807744656	-3.109532200	-1.830062052
H	6.697904552	-2.773226945	-2.876571060
H	7.438163885	-4.017315561	-1.840847991
H	7.332698012	-2.324666601	-1.270572525
C	4.780881432	-4.398540931	-1.936124614
H	4.535555896	-4.082158814	-2.965801822
H	3.845903842	-4.630777866	-1.413303318
H	5.379593688	-5.323811368	-2.009760104
C	7.745828331	-2.869640939	1.600886794

H	7.163148404	-3.651309482	1.096394325
H	7.803487801	-3.115568567	2.676367823
H	8.777692004	-2.889771476	1.205019211
C	7.831884617	-0.502883868	2.056768549
H	7.296630032	0.443052178	1.909905892
H	8.857891727	-0.393523567	1.661550616
H	7.916634100	-0.697811158	3.140651680
B	0.313338018	3.628172127	-0.620767051
C	1.803611026	3.256893532	-0.886161486
C	2.402375798	3.369616023	-2.255401495
H	3.015313228	4.284469134	-2.336449108
H	1.616387269	3.444655045	-3.021439142
H	3.063099050	2.521139149	-2.498364159
C	-0.740271965	3.308487499	-1.750494696
C	-0.838284000	2.012645184	-2.310552195
C	-1.772825579	1.762971399	-3.319417481
H	-1.840101962	0.752541987	-3.730546313
C	-2.623442372	2.755550557	-3.804886456
C	-2.520935172	4.031344404	-3.245798612
H	-3.174141419	4.830111490	-3.611576287
C	-1.607856678	4.318816442	-2.229282364
C	0.009366513	0.858096428	-1.842659688
H	1.043552463	0.926898953	-2.214772853
H	-0.412063315	-0.093377017	-2.198224348
H	0.081187123	0.803180090	-0.746715094
C	-3.639177987	2.455752568	-4.868846100
H	-4.643523650	2.324062434	-4.431285007
H	-3.393887341	1.531601421	-5.411372263
H	-3.712843609	3.274681900	-5.600277852
C	-1.567766776	5.715322785	-1.668155367
H	-1.890200273	5.734601771	-0.615878173
H	-2.222789846	6.388556263	-2.238831331
H	-0.552549791	6.140969204	-1.691883564

C	-0.100915845	4.252555317	0.763780156
C	-1.170430271	3.720081833	1.523088595
C	-1.504293901	4.290292238	2.753324869
H	-2.324943334	3.855147457	3.332351578
C	-0.832057028	5.405199305	3.257241874
C	0.202462704	5.942758986	2.490360417
H	0.728925685	6.830784240	2.854252496
C	0.586459083	5.381893281	1.270018307
C	-1.965429286	2.537957777	1.043661521
H	-2.509444988	2.766833626	0.115838158
H	-2.697175834	2.225558608	1.800540682
H	-1.324143693	1.670624132	0.823329488
C	-1.196332973	5.990928615	4.589826960
H	-2.271102364	5.883576332	4.798041073
H	-0.939070342	7.058584991	4.646220459
H	-0.654633861	5.480234258	5.403955580
C	1.715171384	6.036877658	0.516084306
H	1.887991138	7.058309713	0.884412724
H	1.517806882	6.099038257	-0.564058539
H	2.652505517	5.471895059	0.631180334
H	1.874627968	2.558633108	1.095879706
H	-0.510189609	-2.111053938	-0.524826102
B	-2.794582046	-2.494301718	0.625395627
C	-1.393351928	-2.771568958	1.255396303
C	-1.258827102	-3.342296226	2.639253172
H	-0.598726666	-4.226357984	2.641349026
H	-2.235422059	-3.640479488	3.044006493
H	-0.820045038	-2.617958148	3.346004489
C	-4.079128761	-2.430062622	1.543297265
C	-4.141020427	-1.566830740	2.659046888
C	-5.294691961	-1.519088787	3.451041399
H	-5.325187282	-0.828113197	4.299944960
C	-6.401468664	-2.320198291	3.188768719

C	-6.335663992	-3.175889307	2.084213100
H	-7.193402209	-3.816559122	1.855557663
C	-5.214633703	-3.234126628	1.258124953
C	-3.020078844	-0.631769107	3.028450540
H	-3.394196084	0.399761916	3.119253239
H	-2.205619870	-0.626859226	2.293037311
H	-2.587310224	-0.899920729	4.005654193
C	-7.626083669	-2.279610979	4.055166334
H	-8.533242543	-2.098815148	3.457215744
H	-7.557305549	-1.489173619	4.815804193
H	-7.773089902	-3.238256269	4.578998819
C	-5.249768543	-4.170235917	0.079995533
H	-5.261856613	-3.617499622	-0.871570889
H	-6.143841737	-4.808461129	0.115629574
H	-4.370464464	-4.830491741	0.048029460
C	-2.941803057	-2.303576321	-0.937903257
C	-3.574129980	-1.143185045	-1.447491444
C	-3.752923574	-0.990292530	-2.822410594
H	-4.242481407	-0.084632321	-3.191670669
C	-3.323237881	-1.955657123	-3.735732986
C	-2.707543163	-3.098917566	-3.228396353
H	-2.379460460	-3.879523954	-3.922460417
C	-2.508405911	-3.288028253	-1.856499985
C	-4.064367949	-0.048927573	-0.539593696
H	-4.999959887	-0.336101066	-0.034516551
H	-4.245433755	0.875653227	-1.105844436
H	-3.336807182	0.184804853	0.249334663
C	-3.494232148	-1.748206793	-5.212241600
H	-2.679592200	-1.124474322	-5.619231129
H	-4.439301037	-1.233092308	-5.440253752
H	-3.479963423	-2.701018349	-5.760726616
C	-1.855167453	-4.576670767	-1.423907686
H	-2.141980464	-5.399089213	-2.096126605

H	-2.119271584	-4.865095922	-0.398248708
H	-0.755752749	-4.498954103	-1.451806652

References

[1] H. Nöth, H. Schick, W. Meister, *J. Organomet. Chem.* **1964**, *1*, 401–410.

[2] M. Schwarz, M. Garnica, F. Fasano, N. Demitri, D. Bonifazi, W. Auwärter, *Chem. Eur. J.* **2018**, *24*, 9565–9571.

[3] F. A. Cruz, V. M. Dong, *J. Am. Chem. Soc.* **2017**, *139*, 1029–1032.

[4] K. Smith, A. Pelter, Z. Jin, *Angew. Chem.* **1994**, *106*, 913–914.

[5] A. Pelter, S. Singaram, H. Brown, *Tetrahedron Lett.* **1983**, *24*, 1433–1436.

[6] R. H. Cragg, T. J. Miller, *J. Organomet. Chem.* **1982**, *235*, 143–150.

[7] R. Goetze, H. Nöth, H. Pommerening, D. Sedlak, B. Wrackmeyer, *Chem. Ber.* **1981**, *114*, 1884–1893.

[8] G. M. Sheldrick, *Acta crystallographica. Section A, Foundations and advances* **2015**, *71*, 3–8.

[9] G. M. Sheldrick, *Acta crystallographica. Section A, Foundations of crystallography* **2008**, *64*, 112–122.

[10] M. Ernzerhof, G. E. Scuseria, *J. Chem. Phys.* **1999**, *110*, 5029–5036.

[11] C. Adamo, V. Barone, *J. Chem. Phys.* **1999**, *110*, 6158–6170.

[12] F. Weigend, R. Ahlrichs, *Phys. Chem. Chem. Phys.* **2005**, *7*, 3297–3305.

[13] S. Grimme, J. Antony, S. Ehrlich, H. A. Krieg, *J. Chem. Phys.* **2010**, *132*, 154104.

[14] S. Grimme, S. Ehrlich, L. Goerigk, *J. Comput. Chem.* **2011**, *32*, 1456–1465.

[15] R. Ditchfield, *Mol. Phys.* **1974**, *27*, 789–807.

[16] K. Wolinski, J. F. Hinton, P. Pulay, *J. Am. Chem. Soc.* **1990**, *112*, 8251–8260.

[17] J. R. Cheeseman, G. W. Trucks, T. A. Keith, M. J. Frisch, *J. Chem. Phys.* **1996**, *104*, 5497–5509.

[18] M. J. Frisch, G. W. Trucks, H. B. Schlegel, G. E. Scuseria, M. A. Robb, J. R. Cheeseman, G. Scalmani, V. Barone, B. Mennucci, G. A. Petersson, H. Nakatsuji, M. Caricato, X. Li, H. P. Hratchian, A. F. Izmaylov, J. Bloino, G. Zheng, J. L. Sonnenberg, M. Hada, M.; Ehara, K. Toyota, R. Fukuda, J. Hasegawa, M. Ishida, T. Nakajima, Y. Honda, O. Kitao, H. Nakai, T. Vreven, J. A. Montgomery Jr., J. E. Peralta, F. Ogliaro, M. Bearpark, J. J. Heyd, E. Brothers, K. N. Kudin, V. N. Staroverov, R. Kobayashi, J. Normand, K. Raghavachari, A. Rendell, J. C Burant, S. S. Iyengar, J. Tomasi, M. Cossi, N. Rega, J. M. Millam, M. Klene, J. E. Knox, J. B. Cross, V. Bakken, C. Adamo, J. Jaramillo, R. Gomperts, R. E. Stratmann, O. Yazyev, A. J. Austin, R. Cammi, C. Pomelli, J. W. Ochterski, R. L. Martin, K. Morokuma, V.G. Zakrzewski, G. A. Voth, P. Salvador, J. J. Dannenberg, S. Dapprich, A. D. Daniels, Ö. Farkas, J. B. Foresman, J. V. Ortiz, J. Cioslowski, D. J. Fox, *Gaussian 16, Revision C.01*; Gaussian, Inc.: Wallingford CT, **2016**.
